# Proteomic analysis of the human retina reveals region-specific susceptibilities to metabolic- and oxidative stress-related diseases

**DOI:** 10.1371/journal.pone.0193250

**Published:** 2018-02-21

**Authors:** Gabriel Velez, Daniel A. Machlab, Peter H. Tang, Yang Sun, Stephen H. Tsang, Alexander G. Bassuk, Vinit B. Mahajan

**Affiliations:** 1 Omics Laboratory, Stanford University, Palo Alto, California, United States of America; 2 Department of Ophthalmology, Byers Eye Institute, Stanford University, Palo Alto, California, United States of America; 3 Medical Scientist Training Program, University of Iowa, Iowa City, Iowa, United States of America; 4 Palo Alto Veterans Administration, Palo Alto, California, United States of America; 5 Jonas Children’s Vision Care, and Bernard & Shirlee Brown Glaucoma Laboratory, Columbia Stem Cell Initiative, Departments of Ophthalmology, Pathology & Cell Biology, Institute of Human Nutrition, Columbia University, New York, New York, United States of America; 6 Department of Pathology & Cell Biology, College of Physicians & Surgeons, Columbia University, New York, New York, United States of America; 7 Department of Pediatrics, University of Iowa, Iowa City, Iowa, United States of America; University of Florida, UNITED STATES

## Abstract

Differences in regional protein expression within the human retina may explain molecular predisposition of specific regions to ophthalmic diseases like age-related macular degeneration, cystoid macular edema, retinitis pigmentosa, and diabetic retinopathy. To quantify protein levels in the human retina and identify patterns of differentially-expressed proteins, we collected foveomacular, juxta-macular, and peripheral retina punch biopsies from healthy donor eyes and analyzed protein content by liquid chromatography-tandem mass spectrometry (LC-MS/MS). Protein expression was analyzed with 1-way ANOVA, gene ontology, pathway representation, and network analysis. We identified a mean of 1,974 proteins in the foveomacular retina, 1,999 in the juxta-macular retina, and 1,779 in the peripheral retina. Six hundred ninety-seven differentially-expressed proteins included those unique to and abundant in each anatomic region. Proteins with higher expression in each region include: heat-shock protein 90-alpha (HSP90AA1), and pyruvate kinase (PKM) in the foveomacular retina; vimentin (VIM) and fructose-bisphosphate aldolase C (ALDOC); and guanine nucleotide-binding protein subunit beta-1 (GNB1) and guanine nucleotide-binding protein subunit alpha-1 (GNAT1) in the peripheral retina. Pathway analysis identified downstream mediators of the integrin signaling pathway to be highly represented in the foveomacular region (P = 6.48 e–06). Metabolic pathways were differentially expressed among all retinal regions. Gene ontology analysis showed that proteins related to antioxidant activity were higher in the juxta-macular and the peripheral retina, but present in lower amounts in the foveomacular retina. Our proteomic analysis suggests that certain retinal regions are susceptible to different forms of metabolic and oxidative stress. The findings give mechanistic insight into retina function, reveal important molecular processes, and prioritize new pathways for therapeutic targeting.

## Introduction

**Fig 1:**
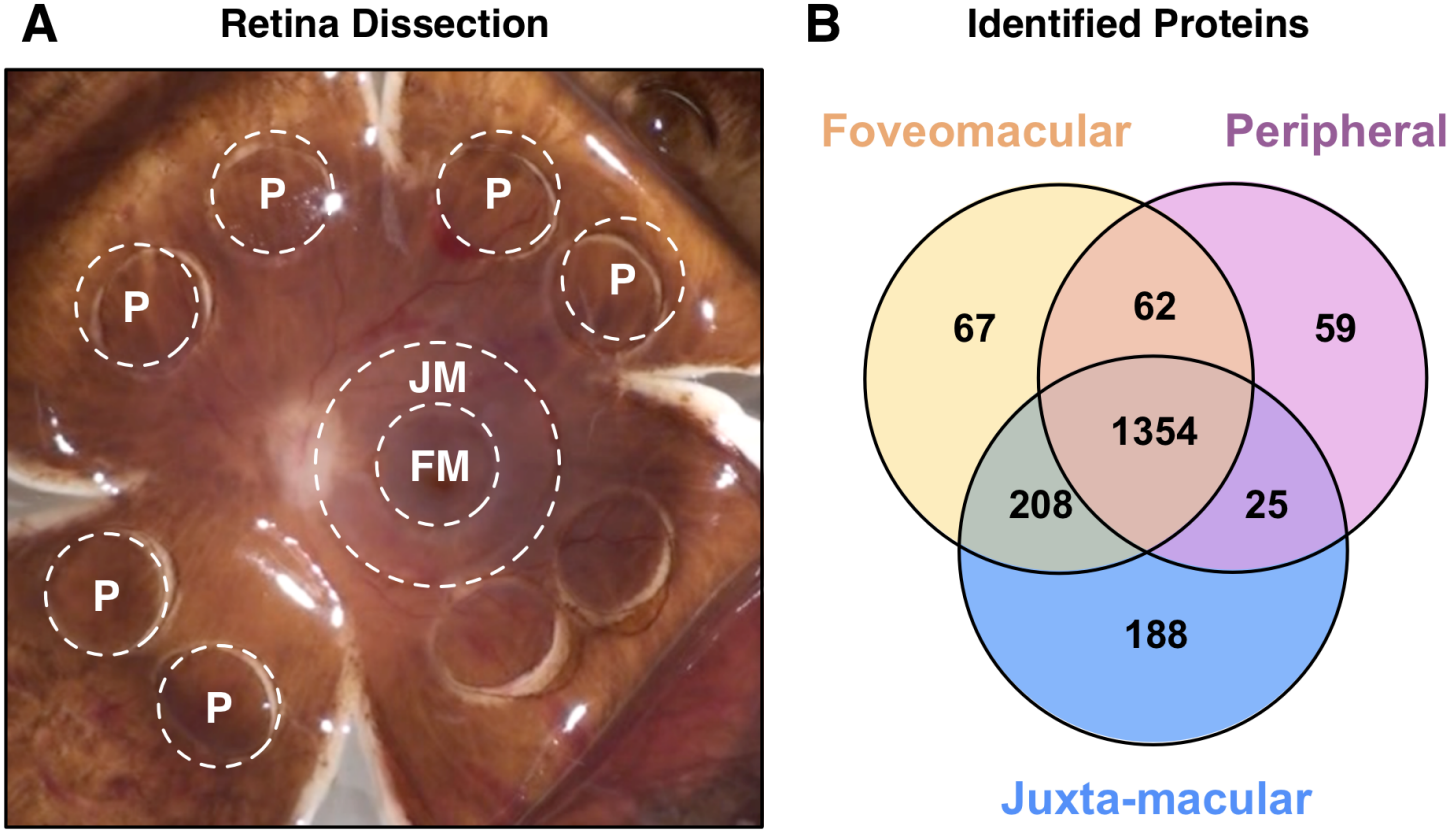
Global analysis of retinal regions. **(A)** Punch biopsy dissection of human retinal regions for proteomic analysis: foveomacular (FM), juxta-macular (JM), and peripheral retina (P). **(B)** Proteins were identified using LC-MS/MS with spectral counts of ≤ 2 were used for further bioinformatics analysis. Venn diagram shows that 1354 proteins are shared among all three regions.

The human retina can be divided into three regions: fovea, macula, and periphery (Fig 1A). They are unique in functionality and susceptibility to diseases such as age-related macular degeneration (AMD), retinitis pigmentosa (RP), proliferative diabetic retinopathy (PDR), and cystoid macular edema (CME). To develop targeted therapies, it is important to understand the molecular basis of their pathophysiology.

Together, regional diseases are responsible for a significant proportion of blindness worldwide. AMD is the leading cause of irreversible central vision loss in elderly patients within the western hemisphere. As the name suggests, the macula is preferentially affected while the periphery remains functional well into advanced disease. RP is the most common form of inherited retinal degeneration and can manifest throughout the lifetime of a patient.[1] Typically, it initiates within the periphery, and the macula and fovea are affected only later. PDR is the advanced stage of diabetic retinopathy, the most common cause of overall vision loss worldwide.[2] It is characterized by profound retinal microvascular disruption and ischemia, leading to uncontrolled neovascularization that can progress to vitreous hemorrhage, retinal detachments, and glaucoma. Finally, CME is a foveal finding that manifests in response to insult from AMD, PDR, RP, and uveitis, thus it is a common finding among numerous diseases.[3]

Oxidative stress is a well-established component of the diseases mentioned above. Elevated levels of reactive oxygen species (ROS) can cause damage to DNA, proteins, and lipids, leading to apoptosis and genetic dysregulation.[4] The retina is the most metabolically-active tissue in the body, making it particularly susceptible to oxidative stress.[5] As one ages, the inherent cellular defenses against ROS decline. While this phenomenon occurs throughout the retina, many diseases preferentially manifest in specific regions. What factors predispose these retinal areas to be susceptible to AMD, PDR, RP, and CME?

Proteomic analysis may provide an answer. Studies have shown that protein expression correlates better with disease progression and severity than gene expression.[6–9] Previous gene-transcriptome analyses have been conducted in human retina[10]; however, retinal proteome patterns may be decoupled from gene expression in normal eyes and in disease states. Thus, focusing on gene expression alone may miss key factors.

By uncovering molecular pathways that are regionally distributed, a proteomics-based approach may reveal mechanisms behind regional susceptibilities to oxidative stress. Previous analyses of the RPE-choroid complex and vitreous have provided insight into their molecular composition,[4, 5, 11] however, anatomic regions of the retina were not differentiated.[12] We have developed a reliable and reproducible dissection protocol that makes use of readily-available punch biopsy tools.[13] Our focus on analyzing the proteomic profile of different retinal regions provides further insight into their molecular uniqueness and to understanding the pathophysiology of numerous regional retinal diseases.

## Methods

### Study approval

The study was approved by the University of Iowa’s Institutional Review Board. Human donor tissue was obtained from the Iowa Lions Eye Bank, Iowa City, IA. Full written consent for research was obtained from the donors or the donor’s next of kin in all cases, and all experiments were performed in accordance with the Declaration of Helsinki.

### Human retina sample collection

Human donor tissue was obtained from the Iowa Lions Eye Bank within 4 hours following death. Three eyes were used in this study. They were obtained from an 84-year-old male, an 83-year-old female, and a 71-year-old female. None of the eyes showed signs of retinal disease. Eyes were flowered into four quadrants as previously described. Using a 4-mm biopsy punch, the foveomacular region and peripheral retina were collected and separated from the underlying RPE-choroid. Using an 8-mm biopsy punch, the juxta-macular retina was collected circumferentially around the foveomacular punch and separated from the RPE-choroid. Dissected tissues were flash frozen in liquid nitrogen and stored at -80°C until further processing.

### Protein extraction and digestion

The received tissue samples (100 μg of total protein per sample) were diluted in 2% SDS, 100 mM Tris-HCl, pH 7.6, 100 mM DTT to approximately 0.5 mL volume and heated at 95 C for 10 min. Each sample was then briefly vortexed and sonicated for 10 s using a probe-tip sonicator (Omni International). The samples were then returned to incubate at 95 C for an additional 10 min. Samples were then transferred to a 30 k Amicon MWCO device (Millipore) and centrifuged at 16.1 k RCF for 30 min. Then 400 μL of 8 M urea, 100 mM Tris-HCl, pH 7.6 was added to each device and centrifuged as before and the filtrate discarded. This step was repeated. Then 400 μL of 8M urea, 100 mM Tris-HCl, pH 7.6, 15 mM iodoacetamide was added to each device and incubated in the dark for 30 min. The samples were then centrifuged as before and the filtrate discarded. Then 400 μL of 8 M urea, 100 mM Tris-HCl, pH 7.6 was added to each device and centrifuged as before and the filtrate discarded. This step was repeated. Then 400 μL of 2 M urea, 100 mM Tris-HCl, pH 7.6 was added to each device along with 2.5 μg trypsin (1:40 enzyme-to-substrate ratio). The devices incubated overnight on a heat block at 37 C. The devices were then centrifuged and the filtrate collected. Then 400 μL 0.5 M NaCl was added to each device and centrifuged as before. The filtrate was added to the previously collected filtrate.

### Peptide desalting and fractionation

Digested peptides were desalted using C18 stop-and-go extraction (STAGE) tips. Briefly, for each sample a C18 STAGE tip was activated with methanol, then conditioned with 75% acetonitrile, 0.5% acetic acid followed by 0.5% acetic acid. Samples were loaded onto the tips and desalted with 0.5% acetic acid. Peptides were eluted with 75% acetonitrile, 0.5% acetic acid and lyophilized in a SpeedVac (Thermo Savant) to dryness, approximately 2 h. Peptides were fractionated using SAX STAGE tips. Briefly, for each sample a SAX STAGE tip was activated with methanol, then conditioned with Britton-Robinson buffer (BRB), pH 3.0 followed by BRB, pH 11.5. Peptides were loaded onto the tips and the flow-through collected followed by and five additional fractions by subsequent application of BRB at pH 8.0, 6.0, 5.0, 4.0 and 3.0. Each fraction was desalted using a C18 STAGE tip and lyophilzed as described above.

### Liquid chromatography-tandem mass spectrometry

Each SAX fraction was analyzed by LC-MS/MS. LC was performed on an Agilent 1100 Nano-flow system. Mobile phase A was 94.5% MilliQ water, 5% acetonitrile, 0.5% acetic acid. Mobile phase B was 80% acetonitrile, 19.5% MilliQ water, 0.5% acetic acid. The 150 min LC gradient ran from 5% A to 35% B over 105 min, with the remaining time used for sample loading and column regeneration. Samples were loaded to a 2 cm x 100 um I.D. trap column positioned on an actuated valve (Rheodyne). The column was 13 cm x 100 μm I.D. fused silica with a pulled tip emitter. Both trap and analytical columns were packed with 3.5 um C18 (Zorbax SB, Agilent). The LC was interfaced to a dual pressure linear ion trap mass spectrometer (LTQ Velos, Thermo Fisher) via nano- electrospray ionization. An electrospray voltage of 1.5 kV was applied to a pre-column tee. The mass spectrometer was programmed to acquire, by data-dependent acquisition, tandem mass spectra from the top 15 ions in the full scan from 400–1400 m/z. Dynamic exclusion was set to 30 s.

### Data processing and library searching

Mass spectrometer .RAW data files were converted to .MGF format and then to .CMN format using msconvert and common. Detailed search parameters are printed in the output data files. Peak list data were searched using three algorithms: NCBI’s OMSSA[14] and The GPM’s X!Tandem,[15] and X!Hunter.[16] The Ensembl human protein sequence library (version 37, reviewed) was used in a target-decoy format. For X!Hunter the latest library build available from TheGPM.org at the time of searching was used. CMN files were searched using X!Tandem using both the native and k-score scoring algorithms. MGF files were searched using OMSSA with precursor mass tolerance settings of +/- 20 ppm, product ions mass tolerance of +/- 1.5 Da, and fragment settings of +/- 0.5 Da [17]. XML output files were parsed using MassSieve (NIH). A minimum peptide length of 6 amino acids was used for protein matches. Proteins were required to have 2 or more unique peptides with E-value scores of 0.01 or less. Relative quantitation was performed by spectral counting. Data were normalized based on total spectral counts (hits) per sample. Proteins with a probability of less than 63% were excluded, giving a 5% false discovery rate at the protein level. The mass spectrometry proteomics data have been deposited to the ProteomeXchange Consortium via the PRIDE partner repository with the dataset identifier PXD008462 [18–20] so that other researchers may analyze the data with their desired statistical criteria and cutoffs. A full list of identified proteins, their corresponding peptides, and spectral counts is outlined in the online supplement (S1 Table).

### Statistical and bioinformatic analysis

Results were also saved in Excel as .txt format and were uploaded into the Partek Genomics Suite 6.5 software package. The data was normalized to log base 2, and compared using 1-way ANOVA analysis. Comparative analysis was repeated with foveomacular region, macula, and peripheral retina tissues. All proteins with non-significant (p>0.05) changes were eliminated from the table. The significant values were mapped using the ‘cluster based on significant genes’ visualization function with the standardization option chosen. Proteins with that were consistently identified among all three eyes in each region (and spectral count > 2) were used for further analysis. PANTHER Classification System[21] was utilized to determine the most significant cellular pathways affected by the proteins present in the different retinal regions. Gene ontology analysis also was performed in PANTHER. Pie charts were created for the visualization of GO distributions within the list of proteins under the Batch ID search menu. Pie charts were created for each GO term category including biological process, molecular function, and cellular component. Protein networks and interactomes were constructed using STRING[22] and visualized in Cytoscape as previously described.[11, 13, 23]

### Analysis of retinal transcriptome data

Serial read archive files of GSE40524[24] were downloaded, converted into FASTQ using SRA toolkit. FASTQ formatted raw reads were aligned using the usegalaxy web platform and TopHat.[25–27] The aligned bam files were further processed according the previously established workflow for Cufflinks and associated suite with bias corrections.[27] All sample assemblies were merged via Cuffmerge. Expression data is reported in fragments per kilobase of exon per million fragments mapped (FPKM).

## Results

### Mass spectrometry overview

Foveomacular, juxta-macular, and peripheral retina samples underwent trypsinization and multidimensional liquid chromatography before analysis by tandem mass spectrometry. In the periphery, we identified 1,779 ±51 individual proteins (134,889 ±89 spectra with 7,458 ±192 unique peptides). In the juxta-macular retina, we identified 1,999 ±46 individual proteins (135,183 ±148 spectra with 8,745 ±732 unique peptides), and in the foveomacular retina, we identified 1,974 ±92 individual proteins (135,233 ±76 spectra with 9,083 ±312 unique peptides; S1 Table). The mean total spectra for the 3 samples showed excellent correlation with an SD of 0.08% of mean total. This indicated the protein load for each sample was consistent. The most abundant proteins identified were alpha-enolase (ENO1), gamma-enolase (ENO2), 11 chains of tubulin (TUBA and TUBB), pyruvate kinase (PKM1 and 2), creatine kinase b-type, vimentin, glyceraldehyde-3-phosphate dehydrogenase, histone H2B type 1-D, and histone cluster (H2BG). The most abundant proteins were similar among all three regions. Of these, 66% were confirmed to be present in a previously-published human retina transcriptome dataset (GSE40524; S2 Table).[24]

Each retinal region has different physical properties and functions that suggest that they may contain distinct proteins. To identify those unique to each region, we performed a comparative analysis (Fig 1B). There were 63 ±31 unique proteins in the foveomacular retina, 43 ±7 in the peripheral retina, and 128 ±63 in the juxta-macular retina. We next curated this list to determine which proteins were consistently present in all three eyes. Of those detected in the foveomacular retina, only one was found to be consistent in all three samples: nuclear coactivator receptor 5 (NCOA5). Similarly, only one was found to be consistent in all three peripheral retina samples: ankyrin repeat domain-containing protein 24 (ANKRD24). Finally, of those detected in the juxta-macular retina, 12 were found to be consistent in all three samples: growth hormone inducible transmembrane protein (GHITM), CCR4-NOT transcription complex subunit 1 (CNOT1), membrane protein palmitoylated 6 (MPP6), kelch repeat and BTB (POZ) domain containing 10 (KBTBD10), methylmalonyl CoA mutase (MUT), F-box protein 7 (FBXO7), propionyl CoA carboxylase (PCCA), O-linked N-acetylglucoamine transferase (OGT), FK506 binding protein 8 (FKBP8), atlastin GTPase 1 (ATL1), cytochrome P450 2A13 (CPA2D), and signal recognition particle 72kDa (SRP72). A total of 1,354 proteins were expressed in all three regions.

### Regional protein expression

**Fig 2:**
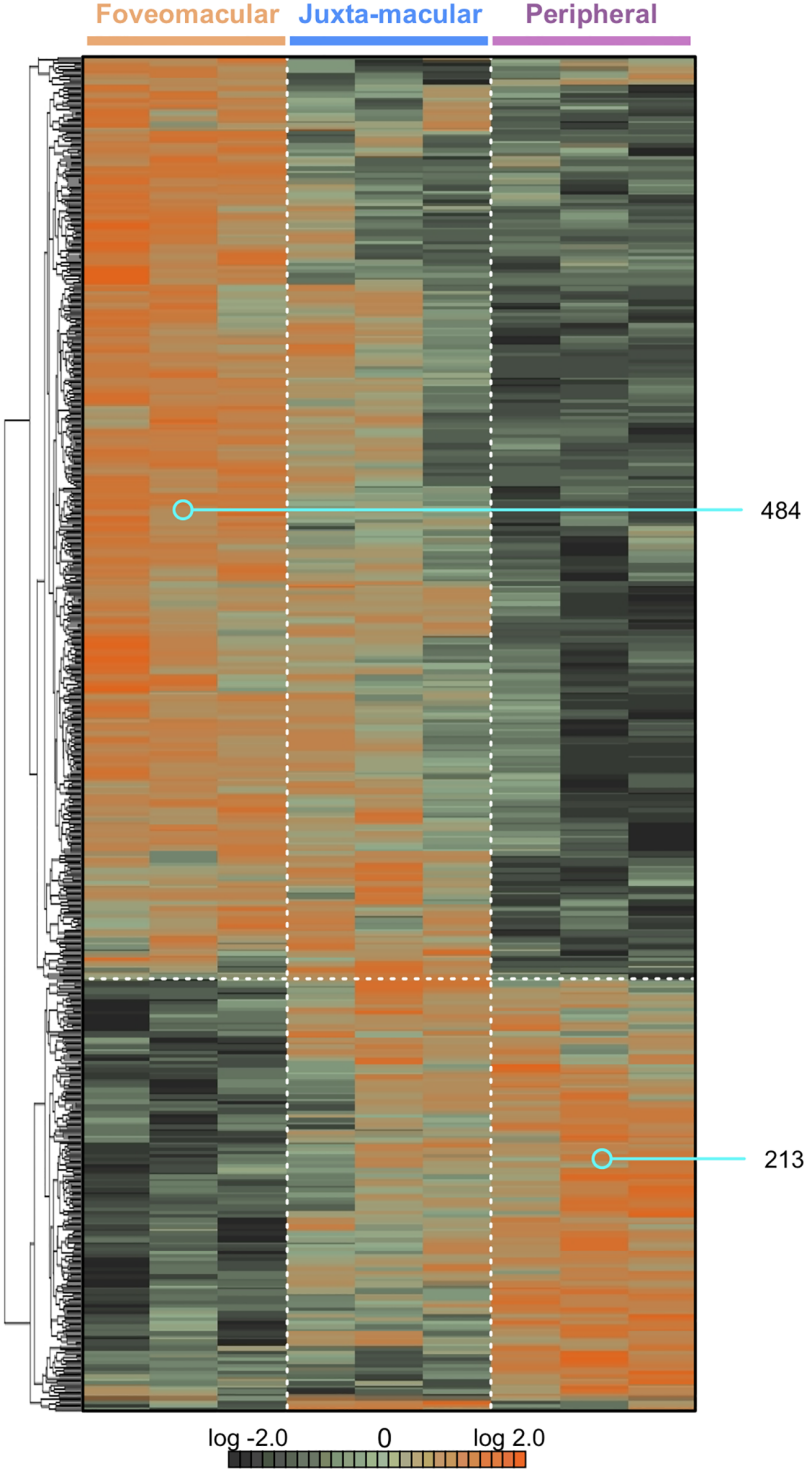
Hierarchical clustering of differentially-expressed proteins in retinal regions. Protein spectral counts were analyzed with 1-way ANOVA and hierarchical heatmap clustering. Results are represented as a heatmap and display protein expression levels on a logarithmic scale. Orange indicates high expression while dark green/black indicates low or no expression. A total of 697 proteins were differentially-expressed among the three groups (p < 0.05). Of these proteins, 484 were highly-expressed in the foveomacular retina. A total of 213 proteins were significantly elevated in the periphery. There was a blend of protein expression in the juxta-macular retina.

Protein spectral counts were analyzed with 1-way ANOVA and hierarchical clustering (Fig 2; p<0.05). A total of 697 proteins were differentially-expressed among the 3 retinal regions. There were 484 proteins highly expressed in the foveomacular region and 213 proteins expressed in the periphery. There was a blend of protein expression observed in the juxta-macular retina (Fig 2). This expression overlap is likely due to the size of punch biopsies. We performed further comparative analysis of these differentially-expressed proteins to identify differences among regions: There were 406 proteins elevated in the foveomacular retina (S3 Table), 314 in the juxta-macular retina (S4 Table), and 188 in the periphery (S5 Table). We next performed 2–way comparisons between each retinal region to determine additional differences (S1, S2 and S3 Figs). Next, we compared the identified proteins in each region to those identified in our previously-published proteomics dataset of the human RPE-choroid [13]. On average, there was 39.8% overlap in protein expression between the retina and RPE-choroid at each anatomic region (S4 Fig). Interestingly, there were fewer unique proteins in the retina compared to the RPE-choroid (11.7% vs. 48.5% average, respectively).

### Gene ontology analysis

**Fig 3:**
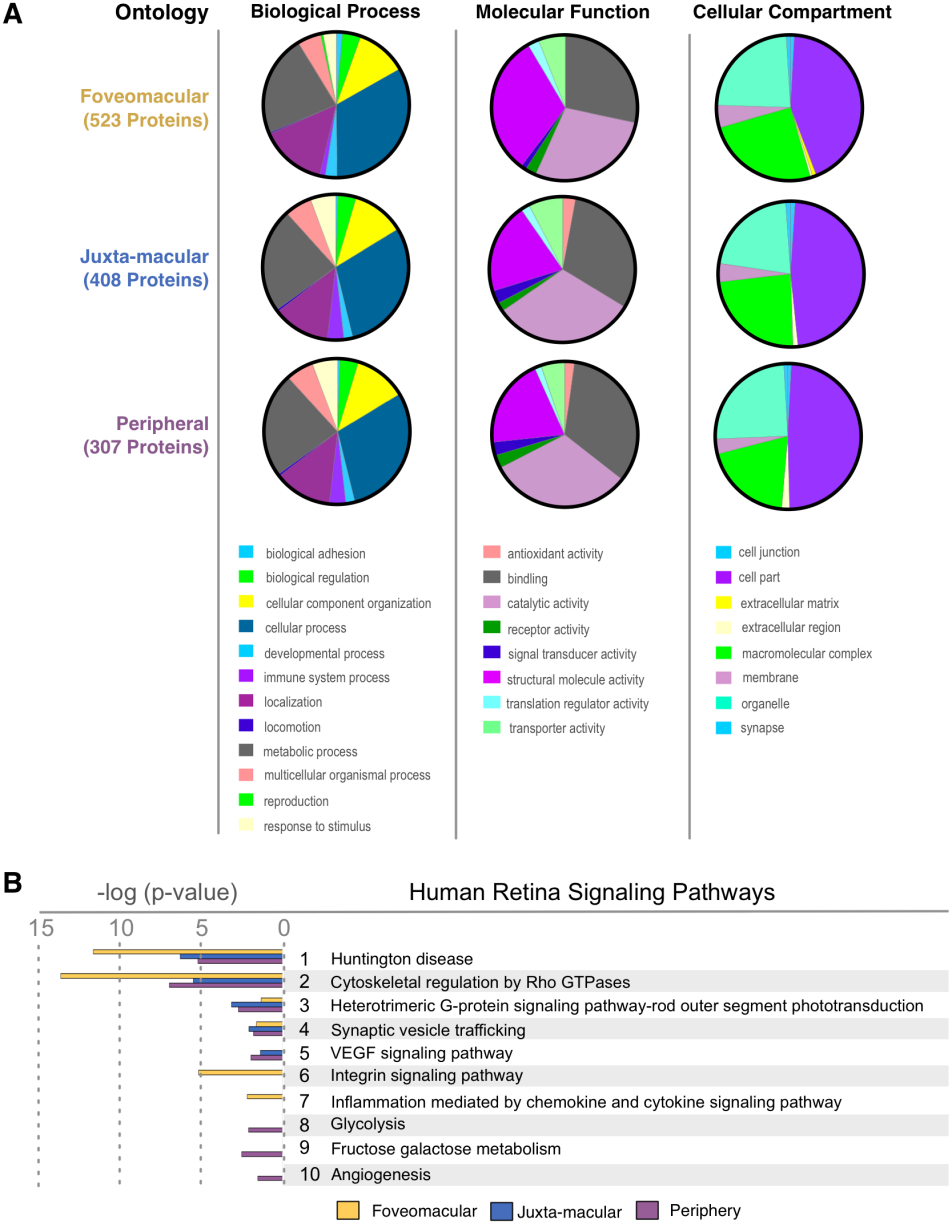
Gene ontology distributions of retina regions highlight tissue differences. **(A)** Differentially-expressed proteins from the foveomacular, juxta-macular, and peripheral retina. Gene ontology analysis categorized each protein group by biological process, molecular function, and cellular compartment. **(B)** Top pathways represented in the three retina regions. Pathways are ranked by their log (p-value), obtained from the right-tailed Fisher Exact Test.

To obtain a global view of the three distinct retina regions, a gene ontology analysis was performed. When comparing the three total protein profiles, the gene ontology summaries were similar. The highest represented categories were cellular process, binding, catalytic activity, and intracellular regions (S5 Fig). After identification of differentially expressed proteins within the regions (Fig 2), differences in gene ontology (GO) categorization emerged. The foveomacular retina had more proteins in the structural molecule activity category than the other two regions. The juxta-macular and peripheral retina had proteins with antioxidant activity, whereas the foveomacular retina did not. The foveomacular region was the only region with proteins in the extracellular matrix category. In the biological process category, only the foveomacular region showed reproduction category proteins. In the cellular component category, every tissue had similar representation, except there was more macromolecular complex category proteins in the foveomacular region (Fig 3A). Together, this indicated that each retinal region expresses distinct functional categories of proteins.

### Molecular pathways

Molecular pathway analysis identifies groups of functionally-linked proteins. Analyzing all regions together, the top pathways were cytoskeletal regulation by Rho GTPase, Huntington and Parkinson disease (known synaptic signaling pathways), and rod outer segment (OS) phototransduction S6 Fig).[28, 29] Next, from the list of differentially-expressed proteins (Fig 2), highly represented pathways were determined. The top foveomacular pathways included cytoskeletal regulation by Rho GTPase, Huntington disease, and integrin signaling (S6 Fig; S6 Table). The most represented juxta-macular pathways were Huntington disease, cytoskeletal regulation by Rho GTPase, Parkinson disease, and heterotrimeric G-protein signaling pathway-rod OS phototransduction (S6 Fig; S7 Table). The peripheral pathways were cytoskeletal regulation, Huntington disease, Parkinson disease and apoptosis signaling (S6 and S8 Tables). Notably, VEGF signaling mediators were present in the juxta-macular and peripheral retina, but absent in the foveomacular region (Fig 3B).

### Proteomic analysis highlights regional expression of retinal degeneration biomarkers

Proteins associated with inherited retinal degenerations were detected in our dataset (Table 1). The proteins with the greatest peripheral retina expression were S-arrestin (SAG), guanine nucleotide-binding protein subunit alpha-1 (GNAT1), retinaldehyde-binding protein 1 (RLBP1), and cyclic nucleotide gated channel beta 1 (CNGB1). Mutations in the *SAG* gene are known to cause Oguchi disease (OMIM: 258100), a rare autosomal recessive form of congenital stationary night blindness, while *GNAT1* mutations cause autosomal dominant congenital stationary night blindness (OMIM: 139330).[30] *RLBP1* and *CNGB1* gene mutations cause autosomal recessive retinitis pigmentosa (arRP).[31, 32] Patients affected by arRP first lose peripheral vision followed by central vision with progression.[33] We detected higher levels of phosphodiesterase 6C (PDE6C) and retinoschisin 1 (RS1) in the foveomacular retina compared to the juxta-macular and peripheral retina (Table 1). Mutations in the *PDE6C* gene cause recessive cone dystrophy (OMIM: 613093), which is characterized by early-onset decreases in visual acuity, impaired color vision, central vision loss, and photophobia.[34] Mutations in the *RS1* gene cause retinoschisis (OMIM: 300839).[35] Levels of rhodopsin (RHO) and retinal G-protein coupled receptor (RGR) were highest in the juxta-macular retina (Table 1). *RHO* and *RGR* mutations both cause autosomal dominant and autosomal recessive RP, respectively (OMIM: 180380 and 600342, respectively).[36, 37]

**Table 1:** Differentially-expressed proteins related to retinal diseases. Our proteomics dataset was interrogated for the presence of retinal disease biomarkers. Spectral count levels are organized by retinal region.

Protein	Associated Diseases	Protein Level (Average Spectra Count ± SD)		
		Foveomacular	Juxta-macular	Peripheral
SAG	Recessive Oguchi disease; recessive retinitis pigmentosa	418 ± 9	537 ± 25	672 ± 98
RLBP1	Recessive retinitis pigmentosa; recessive Bothnia dystrophy; recessive retinitis punctata albescens; recessive Newfoundland rod-cone dystrophy	329 ± 39	393 ± 6	469 ± 62
GNAT1	Dominant congenital stationary night blindness, Nougaret type; recessive congenital stationary night blindness	290 ± 41	447 ± 36	494 ± 59
RHO	Dominant retinitis pigmentosa; dominant congenital stationary night blindness; recessive retinitis pigmentosa	236 ± 43	363 ± 51	358 ± 41
PDE6C	Recessive cone dystrophy, early onset; recessive complete and incomplete achromatopsia	14 ± 7	8 ± 5	1 ± 2
CNGB1	Recessive retinitis pigmentosa	18 ± 5	38 ± 3	44 ± 6
BSG	Impaired retinal function in a mouse model	93 ± 9	87 ± 7	74 ± 6
RGR	Recessive retinitis pigmentosa; dominant choroidal sclerosis; protein	7 ± 5	8 ± 2	3 ± 5
RS1	Retinoschisis	55 ± 10	45 ± 14	35 ± 3
VWF	Age-related macular degeneration	6 ± 2	5 ± 1	0 ± 0

### Cell adhesion protein distributions highlight regional homeostasis of vascular permeability

Cell-adhesion molecules are involved in endothelial cell integrity and vascular homeostasis, particularly at the blood-retina barrier.[38] Endothelial cells are anchored to the basement membrane, which is composed of proteins such as laminin, heparin sulfate proteoglycan (HSPG), and type IV collagen.[39] Disruption of endothelial cell integrity is related to numerous pathological processes, including CME. We interrogated our dataset for proteins and pathways involved in cell-cell and cell-extracellular matrix adhesions. Interestingly, the integrin signaling pathway was highly-represented in the foveomacular retina, but lower in the juxta-macular retina and peripheral retina (P = 6.48 e-06; Fig 3B). These proteins were downstream effectors of integrin activation including actin (ACTA, ACTB, ACTG, ACTS, and ACTBL), ADP-ribosylation factor (ARF1 and ARF6), laminin (LAMB2 and LAMC1), and type IV collagen (CO4A1). In addition to mediating cell-ECM adhesions, integrin signaling can activate numerous pathways involved in cell growth, division, differentiation, and apoptosis. Notably, laminin levels (particularly LAMB1) were highest in the foveomacular and juxta-macular retina compared to the periphery (P = 0.0483). Laminins are one of the major components of the endothelial basement membrane and help to regulate vascular permeability.[40] Thus, their high levels in the juxta-macular and foveomacular retina may indicate a role in CME development. Additionally, we detected von willebrand factor (VWF) levels in the juxta-macular and foveomacular retina, but not in the periphery (Table 1). Elevated VWF levels are a marker of endothelial injury and are associated with AMD.[41]

### Energy metabolism varies throughout the retina

Metabolic dysregulation drives the pathogenesis of DR. Hyperglycemia in diabetes stimulates the production of mitochondrial ROS, which decrease GAPDH activity and lower NADPH levels in the retina.[4] This activates pathways that lead to endothelial cell damage, causing microvascular complications characteristic of DR. We interrogated our dataset for metabolic pathways: anandamine degradation was highly represented in the foveomacular region (P = 6.64 e–03); the pentose phosphate pathway was highly represented in the juxta-macular retina (P = 3.74 e–02); glycolysis (P = 8.09 e–03) and fructose galactose metabolism (P = 3.01 e–03) were highly represented in the periphery (S7 Fig). These results were consistent with the ALDOA network being the largest unique subnetwork in the peripheral retina (S7 Fig), suggesting that the foveomacular and juxta-macular regions favor anabolic pathways while the peripheral retina favors anaerobic metabolism.

### Antioxidant protein distributions highlight foveomacular susceptibility to oxidative stress

**Fig 4:**
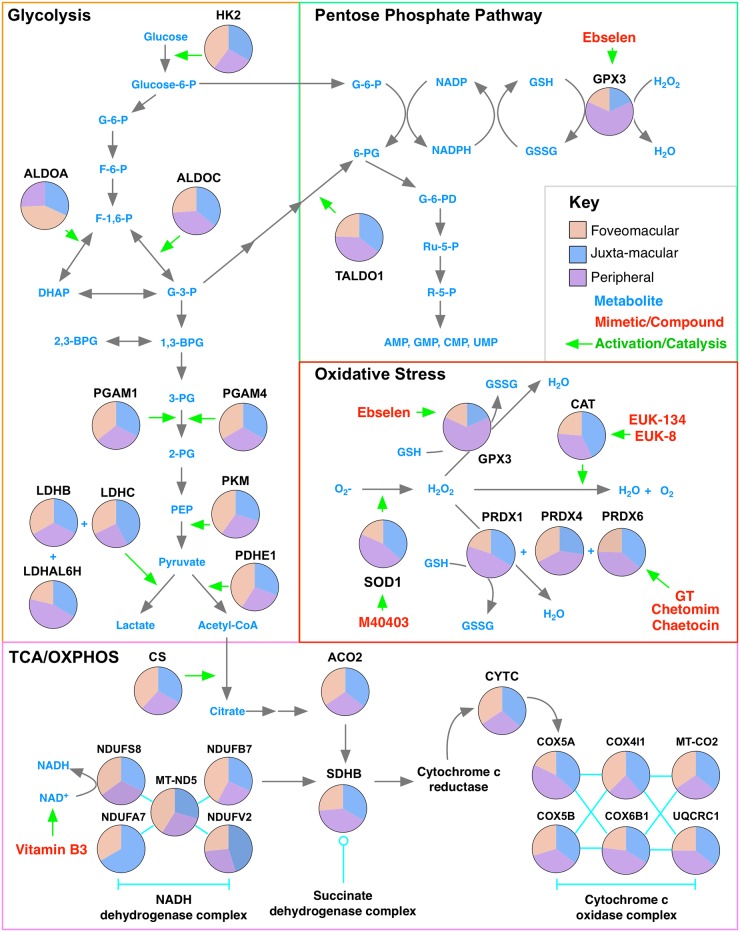
Differential expression of metabolic and antioxidant stress proteins highlights drug repositioning opportunities for retinal disease. Pathway diagram of metabolic and antioxidant proteins with high representation in the human retina. Each pie chart represents the relative protein representation in the foveomacular (orange), juxta-macular (blue), and peripheral retina (purple). Proteins are organized by their molecular pathway and respective metabolites. Compounds and mimetic drugs targeting these specific proteins and metabolites (light blue) are listed in the metabolic map (red). HK2 indicates hexokinase-2; ALDOA, aldolase A; ALDOC, aldolase C; PGAM1, phosphoglycerate mutase 1; PGAM4, phosphoglycerate mutase 4; LDHB, lactate dehydrogenase B; LDHC, lactate dehydrogenase C; LDHAL6H, lactate dehydrogenase A-like 6H; PKM, pyruvate kinase; PDHE1, pyruvate dehydrogenase E1 component subunit alpha; TALDO1, transaldolase; GPX3, glutathione peroxidase 3; CAT, catalase; SOD1, superoxide dismutase 1; PRDX1, peroxiredoxin 1; PRDX4, peroxiredoxin 4; PRDX6, peroxiredoxin 6; CS, citrate synthase; ACO2, aconitate hydratase 2; CYTC, cytochrome c; NDUFS8, NADH dehydrogenase [ubiquinone] iron-sulfur protein 8; NDUFB7, NADH dehydrogenase [ubiquinone] 1 beta subcomplex subunit 7; NDUFV2, NADH dehydrogenase [ubiquinone] flavoprotein 2; NDUFA7, NADH dehydrogenase [ubiquinone] 1 alpha subcomplex subunit 7; MT-ND5, NADH-ubiquinone oxidoreductase chain 5; COX5A, cytochrome c oxidase subunit 5A; COX5B, cytochrome c oxidase subunit 5B; COX4I1, cytochrome c oxidase subunit 4 isoform 1; COX6B1, cytochrome c oxidase subunit 6B1; MT-CO2, cytochrome c oxidase subunit 2; UQCRC1, Cytochrome b-c1 complex subunit 1.

Oxidative stress causes rod and cone dysfunction in diseases like PDR, CME, AMD, and RP.[4] Antioxidant proteins were among the most highly-represented proteins in our dataset. Several were found in the retina, yet our gene ontology analysis indicated that the foveomacular region had lower levels of antioxidant proteins compared to the juxta-macular and peripheral retina (Fig 3). This was further suggested by the finding that the foveomacular retina contained fewer ‘oxidoreductases’ (S8 Fig). The juxta-macular and peripheral retina had higher levels of several antioxidant proteins compared to the foveomacular retina: superoxide dismutase (SOD1), cytochrome oxidase (COX2, COX5A, and CXB1), peroxiredoxin (PRDX1 and PRDX4), Flavin reductase (BLVRB), and aflatoxin B1 aldehyde reductase member 2 (ARK72; S8 Fig). AMD pathogenesis is highly dependent on oxidative stress, and *SOD1* deficiency has been shown to cause dry AMD in a mouse model.[42] Notably, our previous proteomic analysis of human RPE tissue identified high levels of SOD1 as well as elevated glutathione peroxidase (GPX1 and GPX4), PRDX1, PRDX2, PRDX3, and vitronectin (VTN) in the foveomacular retina of the RPE compared to other regions.[13] This difference in the levels of these proteins between the retina and RPE-choroid complex at the foveomacular region may be explained by differences in the molecular function of these tissues. Since the retina and RPE-choroid are comprised of different cell types, it is possible that they have developed unique antioxidant defense systems. Cultured RPE cells have been shown to be tolerate exposure to various oxidants (e.g. H_2_O_2_ and paraquat) and increased light exposure. It is possible that the antioxidant defense systems of the RPE may aid in protecting the retina from the constant barrage of reactive oxygen species, which may be more concentrated at the fovea [43]. Cones are under a considerable amount of oxidative stress and become vulnerable when rods (>90% of population) die.[44] Previous studies have shown cone death during RP begins after most rods have died.[13] This, along with the current findings, suggests that the foveomacular retina is more susceptible to oxidative stress than the juxta-macular and peripheral retina and may be dependent on the antioxidant activity of rods and the RPE.[44] Details of the identified metabolic and antioxidant proteins are highlighted in Fig 4. A protein interaction network was created (S9 Fig).

## Discussion

The human retina is composed of distinct regions that are each susceptible to diseases such as AMD, PDR, RP, and CME (S6 Fig). Proteomic analysis using mass spectrometry is an unbiased method for analyzing global and regional protein expression. Our data demonstrates significant molecular differences between retinal regions that may provide insight into the underlying mechanisms for various diseases. The identified differences in regional protein expression between the retina and RPE-choroid is not surprising as they carry out distinct physiological roles (S4 Fig) [13]. We anticipate that further comparison between these proteomic profiles may elucidate new differences between the molecular functions and pathological susceptibilities of these two tissues.

Our study confirmed the unique molecular functions of various retinal regions. For example, the rod OS phototransduction pathway was highly-represented in the juxta-macular and peripheral retina and had lower representation in the foveomacular region (Fig 3B). We identified elevated levels of cone opsins (e.g green-cone opsin; S3 Table) in the fovea compared to the juxta-macular and peripheral retina. These findings gave us confidence in our approach. Further interrogation of our pathway analysis also provided insight into regional disease processes. We identified high levels of downstream integrin signaling mediators in the foveomacular region. Integrin signaling mediates adhesion to extracellular matrices (ECM) like vitreous. The high-representation of integrin signaling in the foveomacular region may explain its susceptibility to pathologies like vitreomacular traction.[45] The VEGF signaling pathway was also highly-represented in the juxta-macular and peripheral retina, but absent in the foveomacular retina.

The variation of metabolic proteins found within different regions of the retina confirms that each has unique metabolic requirements and functions. Photoreceptors are metabolically robust and require high levels of glucose and oxygen. The high rate of aerobic ATP synthesis produces a significant amount of ROS which can be harmful to the retina.[44] Our analysis showed that the pentose phosphate pathway was highly represented in the juxta-macular retina, suggesting this region favors anabolic metabolism and may be the major source of NADPH (S5 Fig). The large representation of glycolytic pathways and enzymes in the periphery suggest that it favors anaerobic metabolism.

Antioxidant proteins were among the most highly-represented. Previous studies have shown that oxidative stress causes photoreceptor dysfunction.[4] We identified antioxidant proteins that were elevated in both the healthy juxta-macular and peripheral retina such as PRDX1, PRDX4, SOD1, and COX5A (S6 Fig). In diabetes, hyperglycemia stimulates the production of mitochondrial ROS, leading to oxidative stress. This causes a decrease in glyceraldehyde 3-phosphate dehydrogenase (GAPDH) activity, which lowers NADPH levels. Since antioxidant proteins require NADPH (produced by the pentose phosphate pathway) to remove ROS from the retina, hyperglycemia-induced depletion of NADPH leads to chronic oxidative damage.[4, 44] Elevated ROS can also activate inflammatory pathways that cause endothelial cell death and breakdown of the blood-retinal barrier, a potential mechanism for CME.[4] Oxidative stress is also implicated in AMD. The aging retina experiences concurrent increase in ROS levels and decrease in antioxidant capacity, leading to photoreceptor damage and RPE degeneration.[46] Similarly, cellular NAD^+^ levels decrease with age, leaving neurons vulnerable to oxidative damage. Vitamin B3 levels have been shown to be beneficial in replenishing NAD^+^ levels and preventing glaucoma.[47, 48]

Given the differential expression of metabolic and antioxidant proteins in various retinal regions, we considered drugs and mimetic compounds that could preferentially target these pathways (Fig 4; S9 Fig). Such an approach is beneficial in treating or preventing retinal diseases that result from chronic oxidative stress and metabolic dysregulation. For example, the foveomacular region contains the lowest amounts of SOD1 compared to the juxta-macular and peripheral retina, making it highly susceptible to oxidative damage in diseases like DR and RP. M40403, a manganese superoxide dismutase mimetic compound, was effective in preventing oxidative damage in mice following total body irradiation[49] and improved endoneurial blood flow in a diabetic mouse model.[50] Similarly, glutathione peroxidase (GPX3) levels were lowest in the foveomacular region (Fig 4; S8 Fig). Ebselen, a seleno-organic compound and glutathione peroxidase mimetic, reduced ischemic damage in astrocytes.[51] Furthermore, epipolythiodioxopiperazine (ETP) metabolites (e.g. GT, chetomim, and chaetocin) recovered peroxiredoxin (PRDX) activity and reduce oxidative damage and vascular injury.[52] Finally, increased catalase activity can reduce oxidative stress in retinal cells of diabetic mice.[53] Several catalase mimetics (e.g. EUK-8 and EUK-134) were neuroprotective in several animal models of neurodegenerative disease.[54, 55] These drugs may be repurposed for retinopathies where oxidative stress causes damage to retinal glia and vasculature.[56]

Our current study has several limitations. We analyzed retina proteomes from immediate postmortem eyes of elderly patients. Advanced age likely influenced the protein content.[57] Collecting young post-mortem retinas is often not approved by institutional review boards. Thus, our results may not extend to younger patients. Future proteomic studies of samples from younger, non-diseased retinas may further elucidate the age-related changes in the retinal proteome. Furthermore, considerable overlap in retinal protein expression between foveomacular and juxta-macular retina as well as between juxta-macular and peripheral retina (Fig 2) were likely due to the size of punch biopsies.[13] The 4-mm foveal punch dissection also contained the parafovea and perifovea (which overlap substantially with the macula). Similarly, the 8-mm macula punch biopsy also contained portions of the near-peripheral retina outside the anatomic macula.[13] Nevertheless, our analysis could identify distinct and differentially-expressed protein pathways among these regions.

## Conclusions

Proteomic analysis is a powerful tool for studying retinal molecular functions. Unlike gene expression, proteomic analysis can identify dynamic changes in regional protein expression in both healthy and diseased tissue.[6–8] Our dataset points to differential expression of antioxidant and metabolic proteins in the three retinal regions, a finding that has implications for diseases that manifest in unique patterns or localize to specific regions, suggesting that prophylactic targeting of ROS and metabolic reprogramming is essential to treat a variety of retinal diseases. Further interrogation of our dataset should generate additional hypotheses for future validation studies and provide an exciting opportunity to treat the most devastating blinding diseases.

## Supporting information

S1 Results Supplemental results and references. (DOCX)

S1 Fig Differentially-expressed proteins between foveomacular and juxta-macular region. **(A)** Protein spectral counts were analyzed with 1-way ANOVA and heatmap clustering. A total of 268 proteins were differentially-expressed among the two groups (p < 0.05). Of these proteins, 121 were expressed in the foveomacular retina. A total of 147 proteins were significantly elevated in the juxta-macular retina. **(B)** Gene ontology analysis categorized each protein group by biological process, molecular function, and cellular compartment.
(TIFF)

S2 Fig Differentially-expressed proteins between the foveomacular and peripheral retina. Protein spectral counts were analyzed with 1-way ANOVA and heatmap clustering. A total of 358 proteins were differentially-expressed among the two groups (p < 0.05). Of these proteins, 319 were expressed in the foveomacular retina. A total of 38 proteins were significantly elevated in the periphery. **(B)** Gene ontology analysis categorized each protein group by biological process, molecular function, and cellular compartment.
(TIFF)

S3 Fig Differentially-expressed proteins between the peripheral and juxta-macular retina. Protein spectral counts were analyzed with 1-way ANOVA and heatmap clustering. A total of 268 proteins were differentially-expressed among the two groups (p < 0.05). Of these proteins, 195 were expressed in the juxta-macular retina. A total of 73 proteins were significantly elevated in the periphery. **(B)** Gene ontology analysis categorized each protein group by biological process, molecular function, and cellular compartment.
(TIFF)

S4 Fig Regional comparison of protein expression between the retina and RPE-choroid. **(A)** Comparison of proteins identified (spectral count ≥ 2) the foveomacular retina and RPE-choroid. **(B)** Comparison of proteins identified (spectral count ≥ 2) the juxta-macular retina and RPE-choroid. **(C)** Comparison of proteins identified (spectral count ≥ 2) the peripheral retina and RPE-choroid.
(TIFF)

S5 Fig Gene ontology (GO) distributions of human retina proteins show tissue similarity. Identified proteins from the foveomacular, juxta-macular, and peripheral retina. Gene ontology analysis categorized each protein group by biological process, molecular function, and cellular compartment.
(TIFF)

S6 Fig Proteome pathway analysis of human retina regions. **(A)** Top ten pathways represented in all three retina regions. Pathways are ranked by their log (p-value), obtained from the right-tailed Fisher Exact Test, and by their ratio of enrichment, which is equal to the number of observed proteins divided by the number of expected proteins from each pathway that is represented. Top ten pathways based on uniquely expressed proteins are listed for each region: **(B)** foveomacular, **(C)** juxta-macular, and **(D)** peripheral retina.
(TIFF)

S7 Fig Network analysis reveals the largest unique protein networks for retina regions. **(A**) The 406 differentially-expressed proteins from the fovea formed a network with 281 nodes and 1,727 edges. **(B)** The largest hub in the foveomacular network was the EPB41L1 network. **(C)** The 314 differentially-expressed proteins from the juxta-macular retina formed a network with 219 nodes and 811 edges. **(D)** The largest hub in the juxta-macular network was the DYNC1H1 network. **(E)** The 188 differentially-expressed proteins from the juxta-macular retina formed a network with 86 nodes and 171 edges. **(F)** The largest hub in the peripheral network was the ALDOA network.
(TIFF)

S8 Fig Antioxidant proteins are more abundant in the juxta-macular and peripheral retina. **(A)** Gene ontology analysis of differentially-expressed proteins identified the ‘antioxidant activity’ in the juxta-macular and peripheral retina, but not in the foveomacular region. Further gene ontology analysis using protein class categorization revealed the juxta-macular and peripheral retina to have a higher percentage of differentially-expressed oxidoreductase proteins, further suggesting lower antioxidant activity in the foveomacular region. **(B)** Deoxygenase levels in the foveomacular, juxta-macular, and peripheral retina. **(C)** Oxidase levels in the foveomacular, juxta-macular, and peripheral retina. **(D)** Peroxidase levels in the foveomacular, juxta-macular, and peripheral retina. **(E)** Reductase levels in the foveomacular, juxta-macular, and peripheral retina.
(TIFF)

S9 Fig Interaction network of retinal metabolic and antioxidant proteins. Network analysis was performed on the metabolic and antioxidant proteins identified in all three regions using STRING. Only protein-protein interactions verified by experimental data or databases were selected. Nodes are colored by their respective metabolic pathway.
(TIFF)

S10 Fig The SF3B1 network is be the largest subnetwork in all retina regions. Network analysis was performed on the 1,354 shared proteins among all three regions using STRING. Only protein-protein interactions verified by experimental data or databases were selected. The resulting complex network contained 1,157 nodes (proteins) and 16,627 edges (interactions). CytoCluster software was used to generate and rank subnetworks for visualization. The largest subnetwork was the SF3B1 network with 74 nodes and 2,313 edges. This group of proteins is involved in RNA splicing and translation.
(TIFF)

S1 Table Mass spectrometry overview. A full table listing the identified proteins, spectral counts, and peptide sequences is included as a separate attachment. Raw data available in the PRIDE database (PXD008462).
(XLSX)

S2 Table Comparison to previously-published human retina transcriptome. Abundant proteins in our proteomics dataset were verified through comparison to a previously-published transcriptome dataset of the human retina (GSE40524). Expression data is reported in fragments per kilobase of exon per million fragments mapped (FPKM). A total of 66% identified proteins were also present in the RNA-seq dataset.
(DOCX)

S3 Table Differentially-expressed proteins in the foveomacular region. (DOCX)

S4 Table Differentially-expressed proteins in the juxta-macular retina. (DOCX)

S5 Table Differentially-expressed proteins in the peripheral retina. (DOCX)

S6 Table Pathways identified in the foveomacular region. (DOCX)

S7 Table Pathways identified in the juxta-macular retina. (DOCX)

S8 Table Pathways identified in the peripheral retina. (DOCX)
