# Supplementary material for: Proteomic analysis of the human retina reveals region-specific susceptibilities to metabolic- and oxidative stress-related diseases
Source: PLoS One. 2018 Feb 21;13(2):e0193250. doi: 10.1371/journal.pone.0193250 (PMC5821407; doi:10.1371/journal.pone.0193250)
Supplement: S1 Results — (DOCX) [file pone.0193250.s001.docx]

**Supplementary Online Content**

**Proteomic analysis of the human retina highlights region-specific susceptibilities to metabolic- and oxidative stress-related diseases**

Velez G, Machlab DA, Tang PH, Sun Y, Tsang SH, Bassuk AG, Mahajan VB

**Supplemental Results**

*Protein classification*

We identified abundant differentially-expressed proteins in each region. In the foveomacular region, the highest differentially-expressed proteins were heat-shock protein 90-alpha (HSP90AA1), tubulin (TUBA1, TUB4A, and TUBB), pyruvate kinase (PKM), actin (ACTA2, ACTB, and ACTG1), and ATP synthase (ATP5A1). In the juxta-macular retina, the highest differentially-expressed proteins were vimentin (VIM), fructose-bisphosphate aldolase C (ALDOC), histone H2A (HIST2H2AA3), and hemoglobin subunit beta (HBB). In the peripheral retina, the highest differentially-expressed proteins heat-shock protein 90-beta (HSP90AB1), heat shock cognate 71 (HSPA8), spectrin alpha chain (SPTAN1), guanine nucleotide-binding protein subunit beta-1 (GNB1), and guanine nucleotide-binding protein subunit alpha-1 (GNAT1).

*Protein interaction networks*

When the entire proteome was analyzed for interaction networks, the foveomacular region had 1,492 nodes (proteins) and 38,060 edges (interactions), the peripheral retina had 1,332 nodes and 33,055 edges, and the juxta-macular retina had 1,574 nodes and 41,787 edges. Interactions represent established binding, signaling, structural, activation, or inhibition activity between two proteins. The whole proteome networks for these regions were very complex. We therefore chose to analyze subnetworks of proteins for visualization purposes using CytoCluster.[1] We analyzed the 1,354 commonly identified proteins for interaction networks. This resulted in a complex network with 1,157 nodes and 16,627 edges. The largest subnetwork, the splicing factor 3B subunit 1 (SF3B1) network, contained 74 nodes and 2,313 edges (Supplemental Figure 10). SF3B1 and other components of this network form spliceosome complexes. Components of spliceosome complexes have been implicated in the development of retinal degeneration with or without additional developmental abnormalities.[2] We next analyzed differentially-expressed proteins to identify the networks unique to each anatomic region. The largest network hub unique to the foveomacular region is the band 4.1-like protein 1 (EPB41L1) network (Supplemental Figure 6A-B). This protein is involved in mediating interactions between the cytoskeleton and the plasma membrane.[3] These interactions have been suggested to promote stability and plasticity to the neuronal membrane.[3] The largest network hub unique to the juxta-macular retina is the cytoplasmic dynein 1 heavy chain 1 (DYNCH1) network (Supplemental Figure 6C). The largest network hub unique to the peripheral retina is the aldolase A (ALDOA) network (Supplemental Figure 6D-E). This glycolytic enzyme catalyzes the conversion of fructose-1,6-bisphosphate to glyceraldehyde 3-phosphate and dihydroxyacetone phosphate.[4] This result is consistent with the finding that glycolysis-related proteins were highly-represented in the peripheral retina (*P* < 8.09 e–03; Figure 3B and Supplemental Table 8).

**Supplemental References**

1. Shannon P, Markiel A, Ozier O, Baliga NS, Wang JT, Ramage D, et al. Cytoscape: a software environment for integrated models of biomolecular interaction networks. Genome Res. 2003;13(11):2498-504. Epub 2003/11/05. doi: 10.1101/gr.1239303. PubMed PMID: 14597658; PubMed Central PMCID: PMCPMC403769.

2. Xu M, Xie YA, Abouzeid H, Gordon CT, Fiorentino A, Sun Z, et al. Mutations in the Spliceosome Component CWC27 Cause Retinal Degeneration with or without Additional Developmental Anomalies. Am J Hum Genet. 2017;100(4):592-604. Epub 2017/03/14. doi: 10.1016/j.ajhg.2017.02.008. PubMed PMID: 28285769; PubMed Central PMCID: PMCPMC5384039.

3. Hamdan FF, Gauthier J, Araki Y, Lin DT, Yoshizawa Y, Higashi K, et al. Excess of de novo deleterious mutations in genes associated with glutamatergic systems in nonsyndromic intellectual disability. Am J Hum Genet. 2011;88(3):306-16. Epub 2011/03/08. doi: 10.1016/j.ajhg.2011.02.001. PubMed PMID: 21376300; PubMed Central PMCID: PMCPMC3059427.

4. Dalby A, Dauter Z, Littlechild JA. Crystal structure of human muscle aldolase complexed with fructose 1,6-bisphosphate: mechanistic implications. Protein Sci. 1999;8(2):291-7. Epub 1999/02/27. doi: 10.1110/ps.8.2.291. PubMed PMID: 10048322; PubMed Central PMCID: PMCPMC2144250.
