# Supplementary figures and images for: Proteomic analysis of the human retina reveals region-specific susceptibilities to metabolic- and oxidative stress-related diseases
Source: PLoS One. 2018 Feb 21;13(2):e0193250. doi: 10.1371/journal.pone.0193250 (PMC5821407; doi:10.1371/journal.pone.0193250)

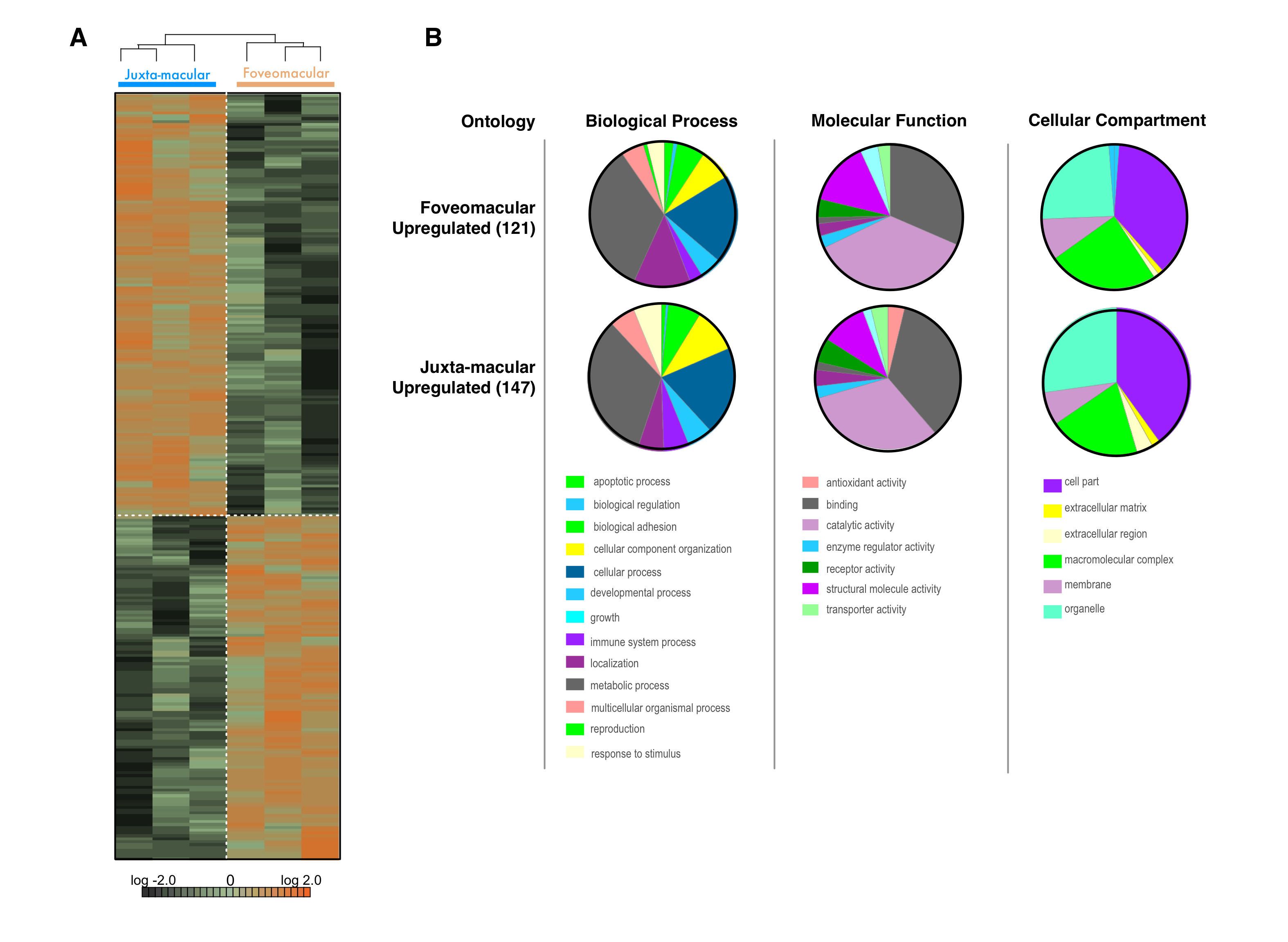

Supplement: S1 Fig — (A) Protein spectral counts were analyzed with 1-way ANOVA and heatmap clustering. A total of 268 proteins were differentially-expressed among the two groups (p < 0.05). Of these proteins, 121 were expressed in the foveomacular retina. A total of 147 proteins were significantly elevated in the juxta-macular retina. (B) Gene ontology analysis categorized each protein group by biological process, molecular function, and cellular compartment. (TIFF) [file pone.0193250.s002.tiff]

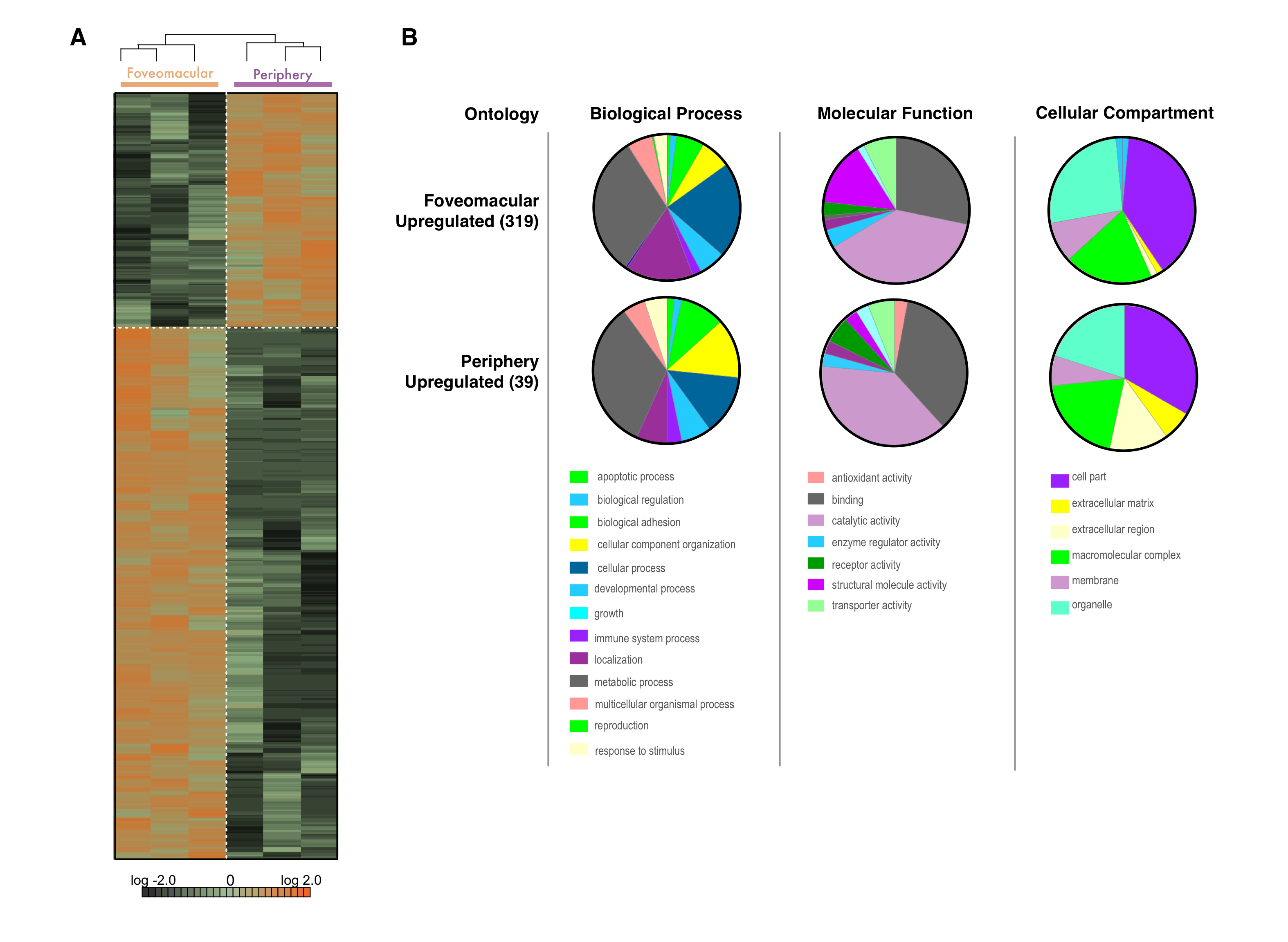

Supplement: S2 Fig — Protein spectral counts were analyzed with 1-way ANOVA and heatmap clustering. A total of 358 proteins were differentially-expressed among the two groups (p < 0.05). Of these proteins, 319 were expressed in the foveomacular retina. A total of 38 proteins were significantly elevated in the periphery. (B) Gene ontology analysis categorized each protein group by biological process, molecular function, and cellular compartment. (TIFF) [file pone.0193250.s003.tiff]

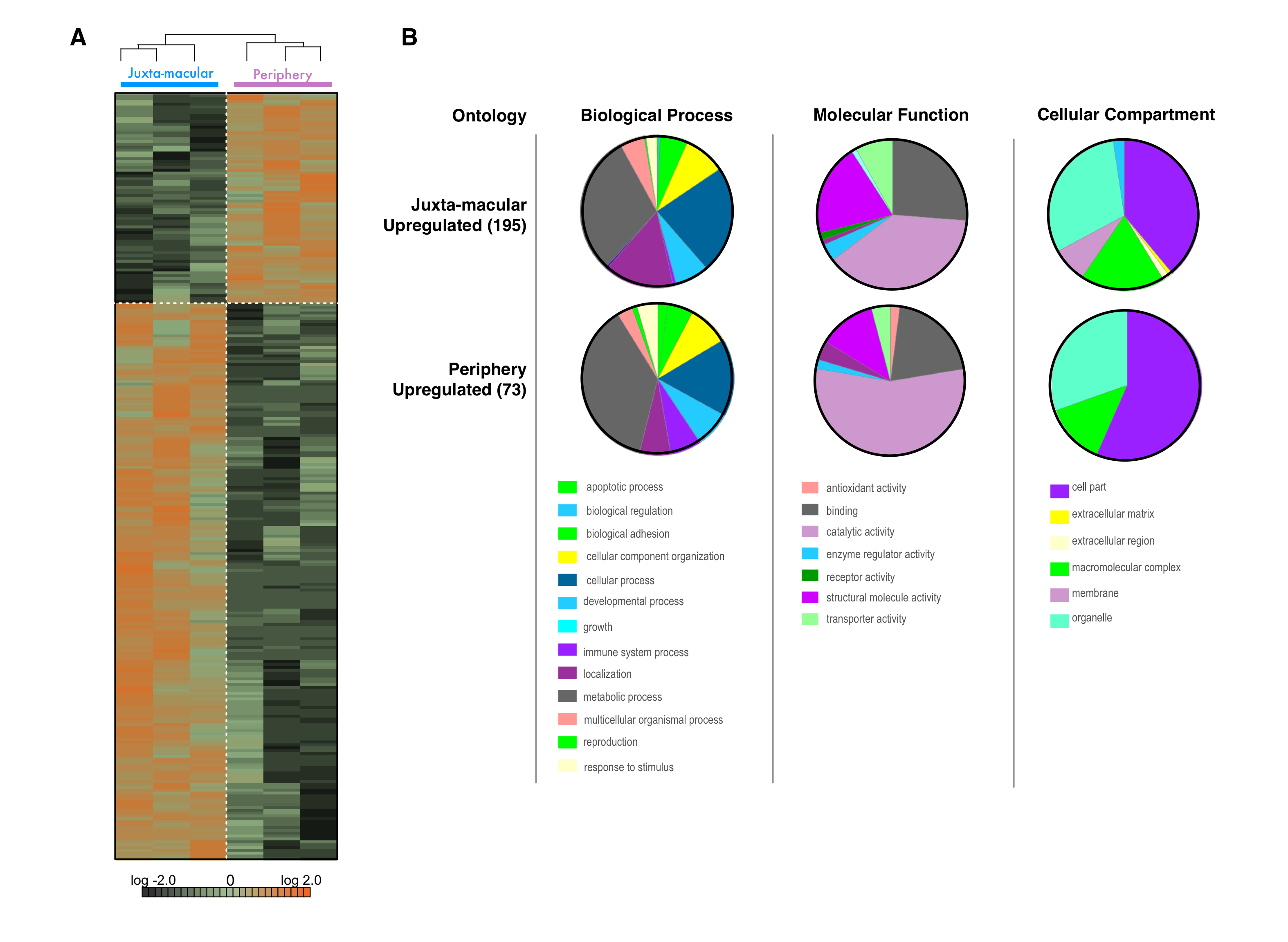

Supplement: S3 Fig — Protein spectral counts were analyzed with 1-way ANOVA and heatmap clustering. A total of 268 proteins were differentially-expressed among the two groups (p < 0.05). Of these proteins, 195 were expressed in the juxta-macular retina. A total of 73 proteins were significantly elevated in the periphery. (B) Gene ontology analysis categorized each protein group by biological process, molecular function, and cellular compartment. (TIFF) [file pone.0193250.s004.tiff]

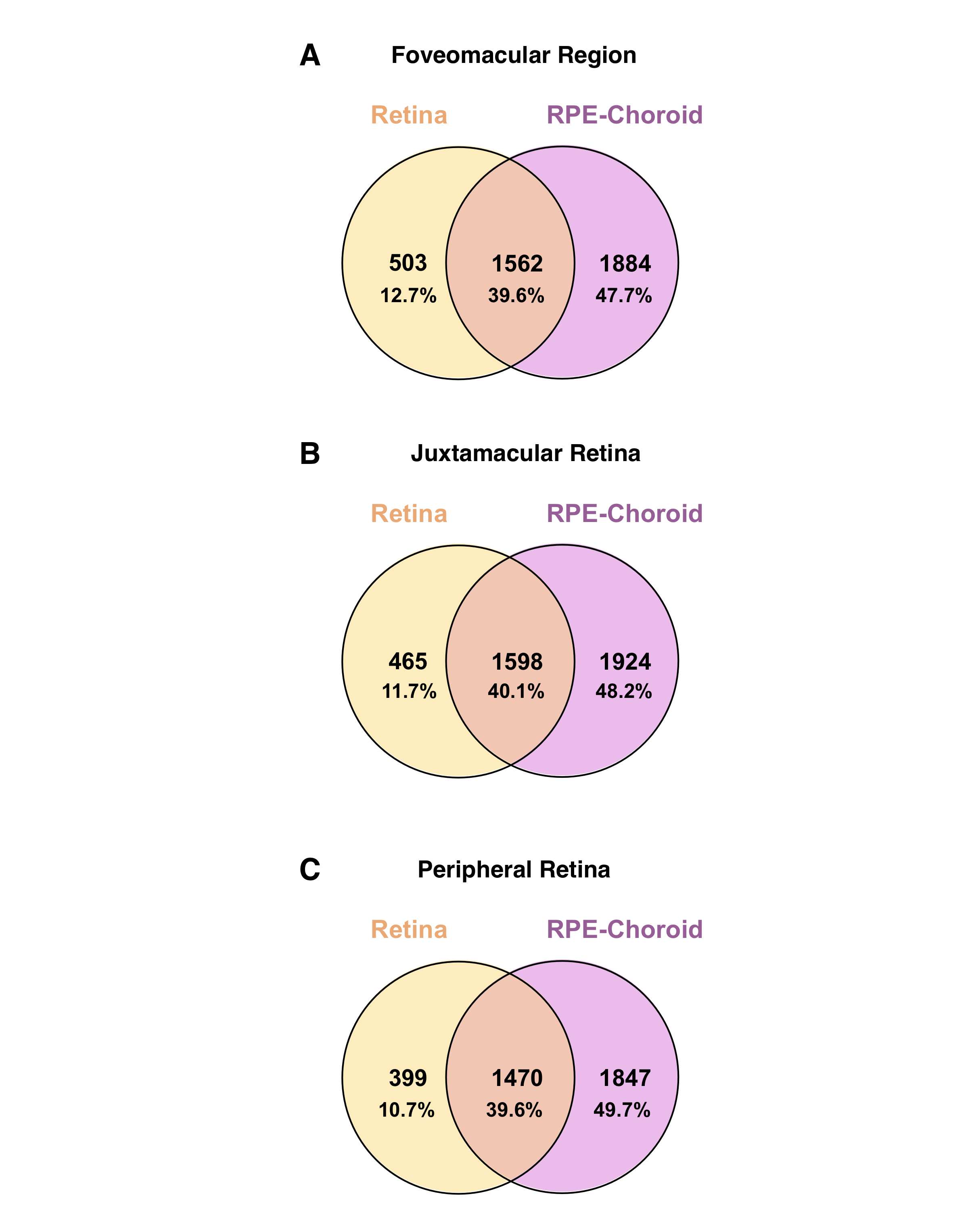

Supplement: S4 Fig — (A) Comparison of proteins identified (spectral count ≥ 2) the foveomacular retina and RPE-choroid. (B) Comparison of proteins identified (spectral count ≥ 2) the juxta-macular retina and RPE-choroid. (C) Comparison of proteins identified (spectral count ≥ 2) the peripheral retina and RPE-choroid. (TIFF) [file pone.0193250.s005.tiff]

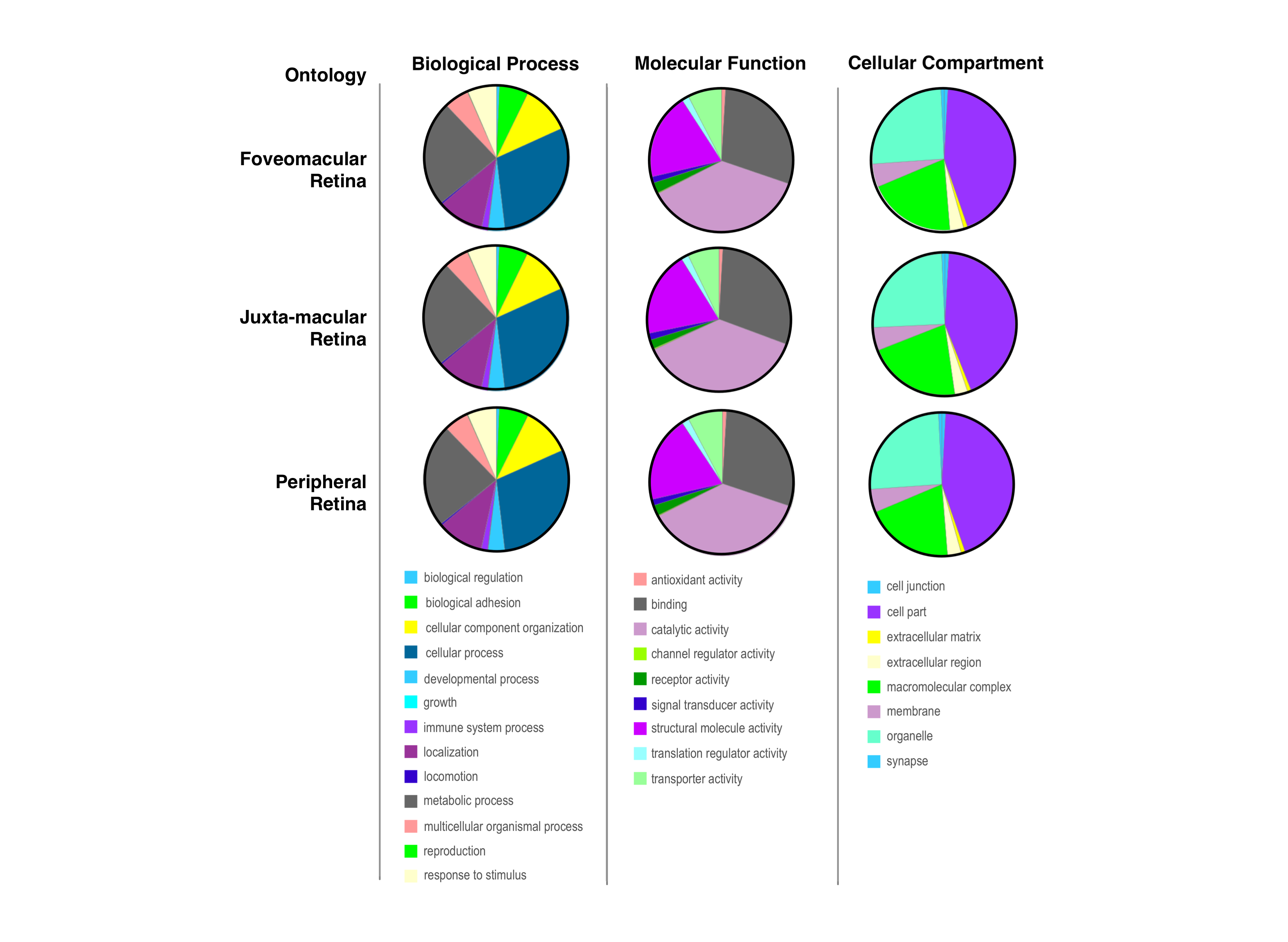

Supplement: S5 Fig — Identified proteins from the foveomacular, juxta-macular, and peripheral retina. Gene ontology analysis categorized each protein group by biological process, molecular function, and cellular compartment. (TIFF) [file pone.0193250.s006.tiff]

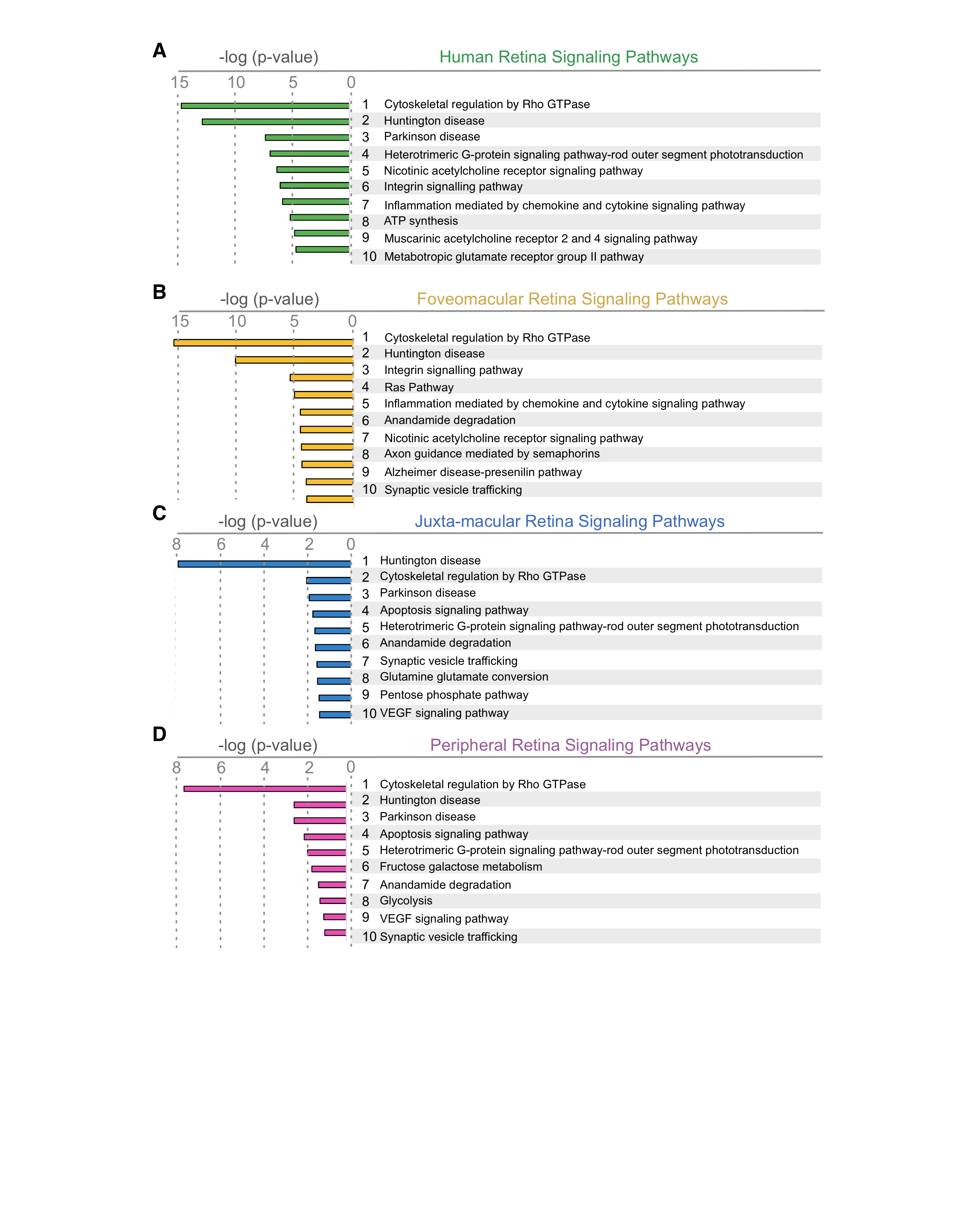

Supplement: S6 Fig — (A) Top ten pathways represented in all three retina regions. Pathways are ranked by their log (p-value), obtained from the right-tailed Fisher Exact Test, and by their ratio of enrichment, which is equal to the number of observed proteins divided by the number of expected proteins from each pathway that is represented. Top ten pathways based on uniquely expressed proteins are listed for each region: (B) foveomacular, (C) juxta-macular, and (D) peripheral retina. (TIFF) [file pone.0193250.s007.tiff]

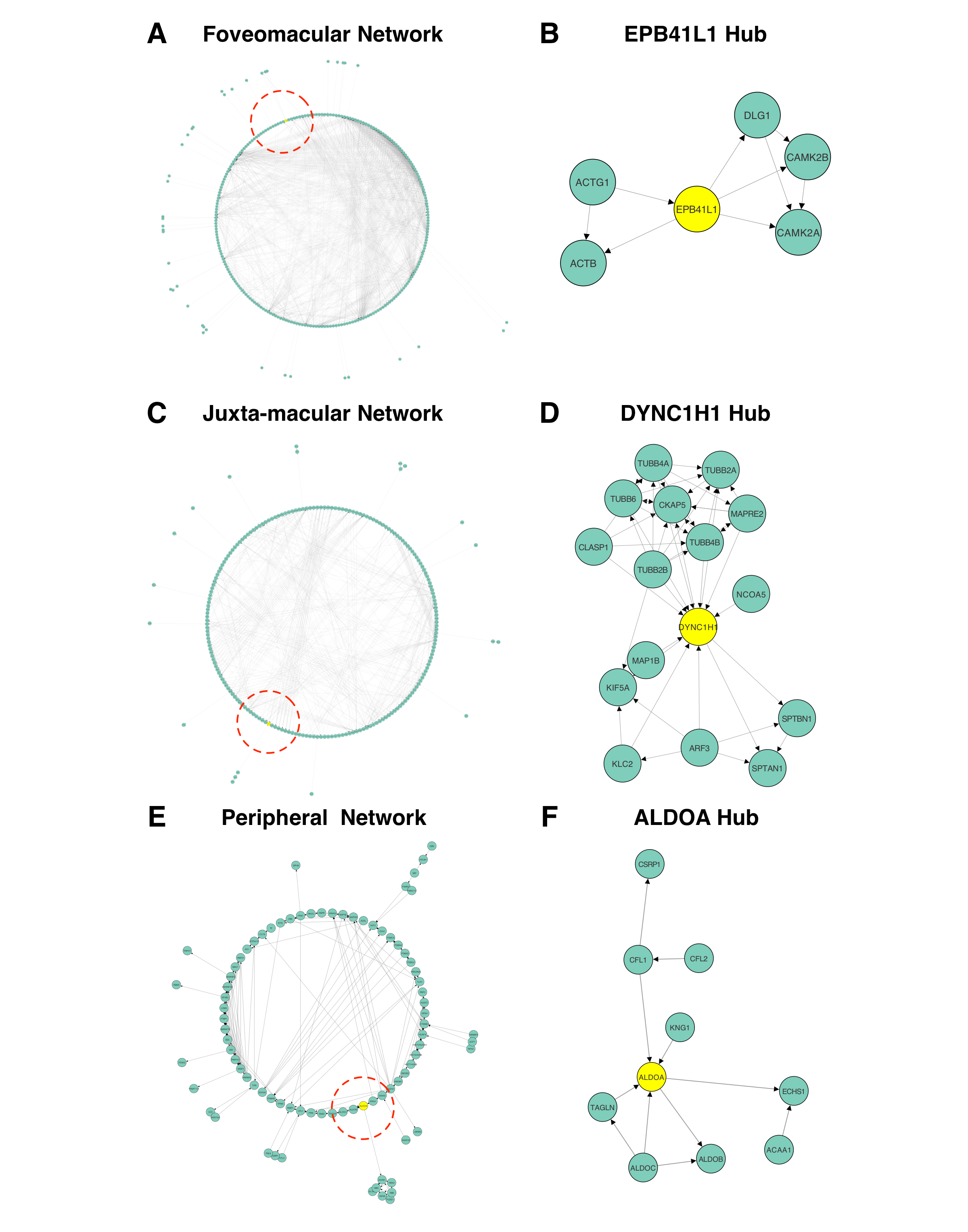

Supplement: S7 Fig — (A) The 406 differentially-expressed proteins from the fovea formed a network with 281 nodes and 1,727 edges. (B) The largest hub in the foveomacular network was the EPB41L1 network. (C) The 314 differentially-expressed proteins from the juxta-macular retina formed a network with 219 nodes and 811 edges. (D) The largest hub in the juxta-macular network was the DYNC1H1 network. (E) The 188 differentially-expressed proteins from the juxta-macular retina formed a network with 86 nodes and 171 edges. (F) The largest hub in the peripheral network was the ALDOA network. (TIFF) [file pone.0193250.s008.tiff]

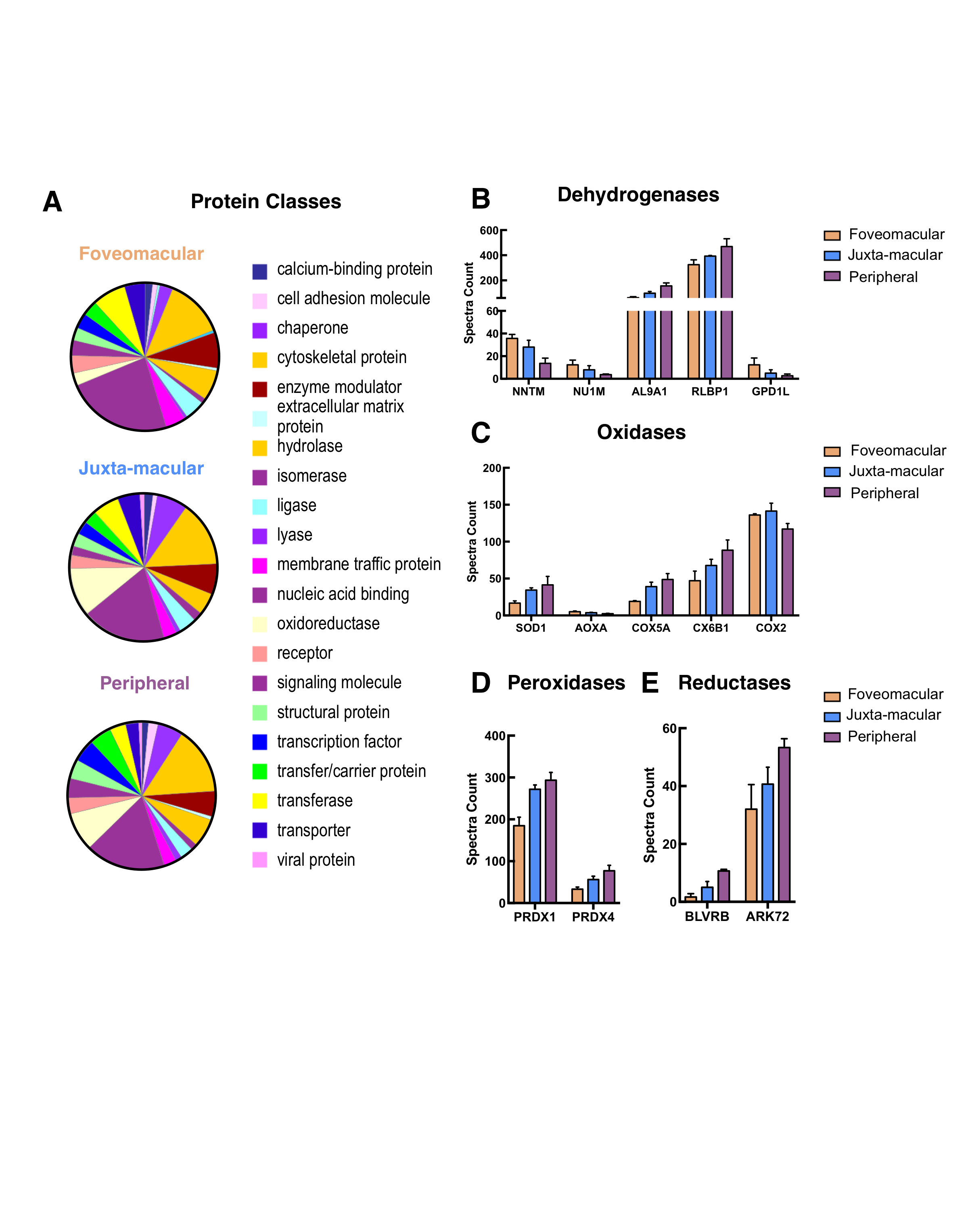

Supplement: S8 Fig — (A) Gene ontology analysis of differentially-expressed proteins identified the ‘antioxidant activity’ in the juxta-macular and peripheral retina, but not in the foveomacular region. Further gene ontology analysis using protein class categorization revealed the juxta-macular and peripheral retina to have a higher percentage of differentially-expressed oxidoreductase proteins, further suggesting lower antioxidant activity in the foveomacular region. (B) Deoxygenase levels in the foveomacular, juxta-macular, and peripheral retina. (C) Oxidase levels in the foveomacular, juxta-macular, and peripheral retina. (D) Peroxidase levels in the foveomacular, juxta-macular, and peripheral retina. (E) Reductase levels in the foveomacular, juxta-macular, and peripheral retina. (TIFF) [file pone.0193250.s009.tiff]

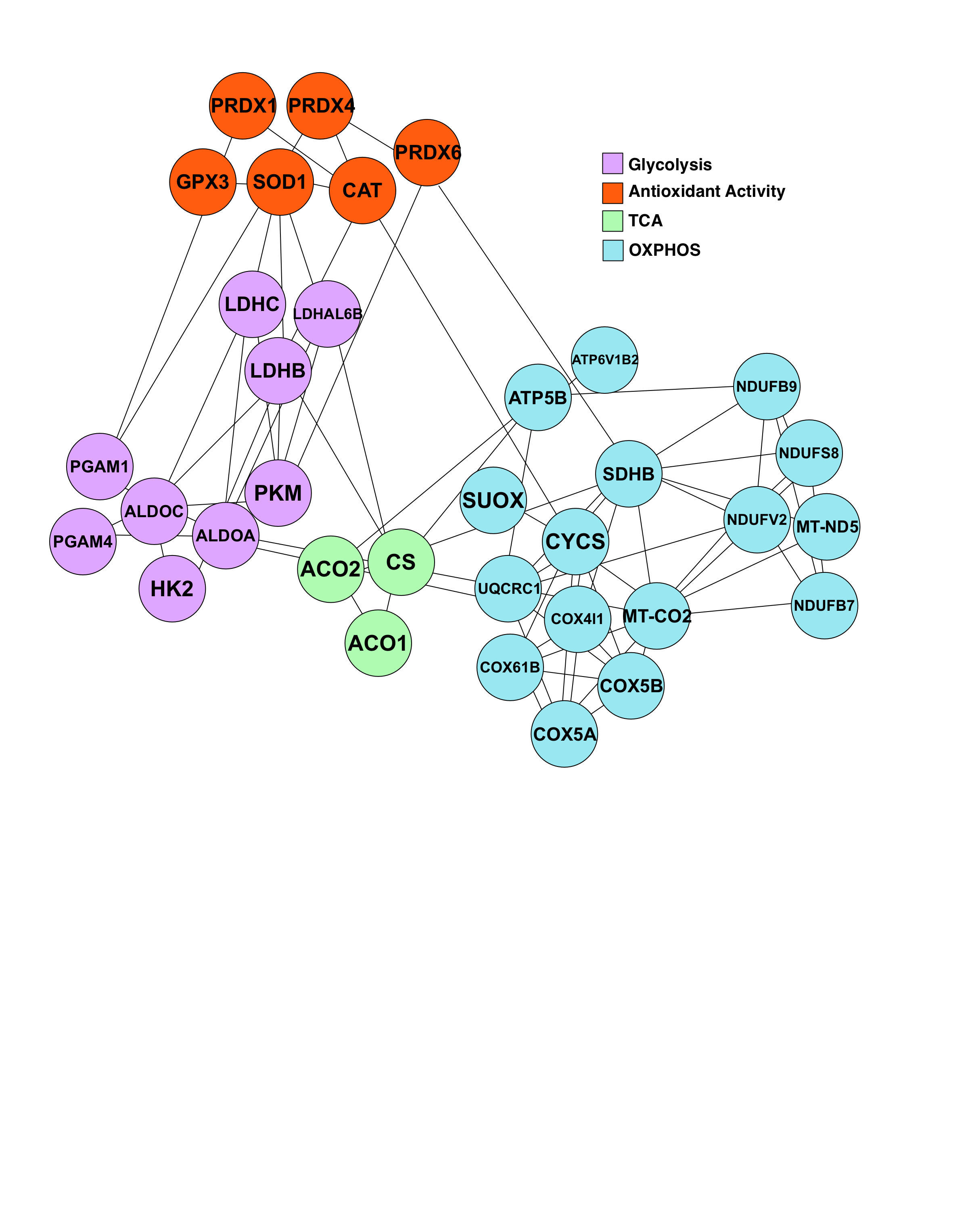

Supplement: S9 Fig — Network analysis was performed on the metabolic and antioxidant proteins identified in all three regions using STRING. Only protein-protein interactions verified by experimental data or databases were selected. Nodes are colored by their respective metabolic pathway. (TIFF) [file pone.0193250.s010.tiff]

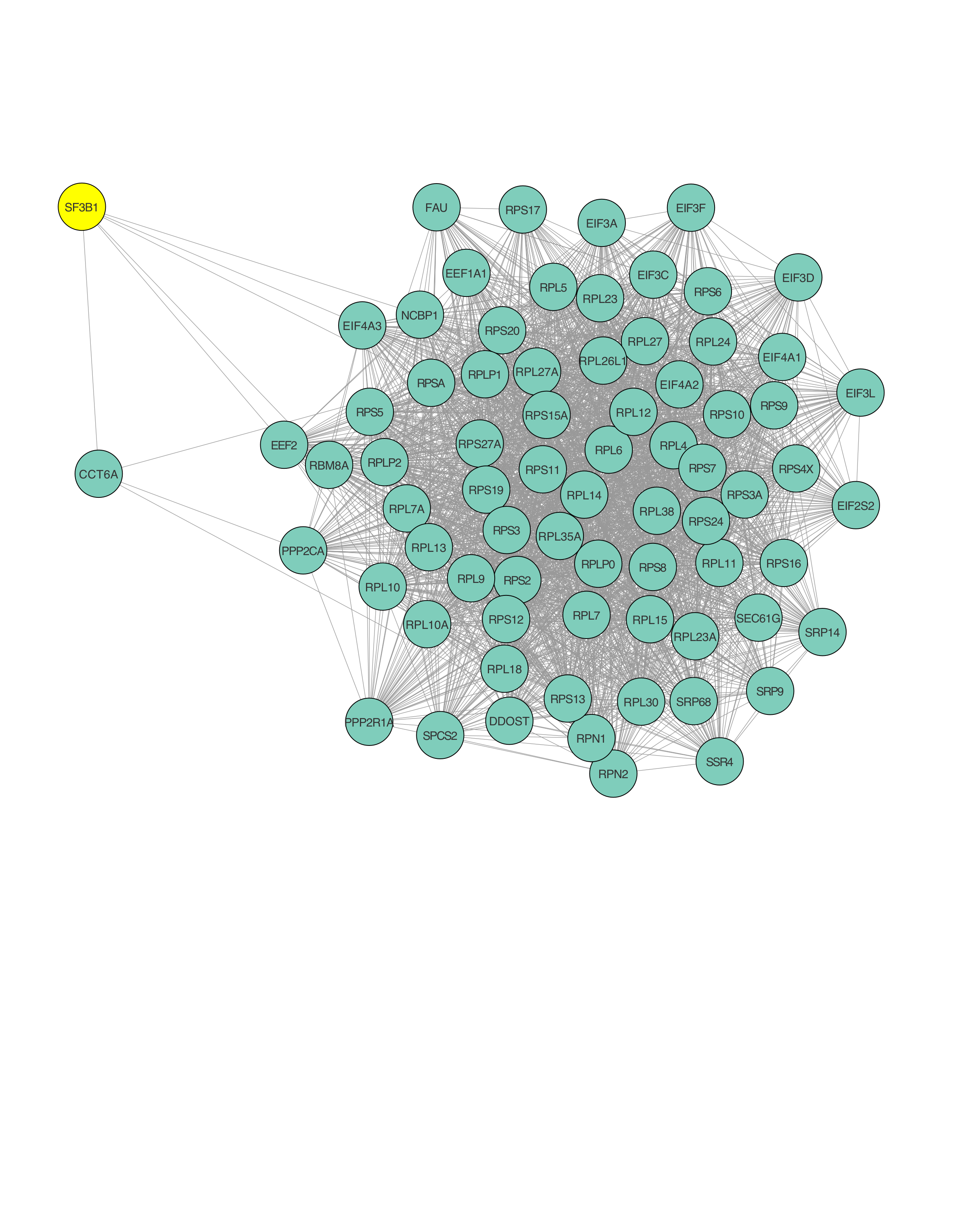

Supplement: S10 Fig — Network analysis was performed on the 1,354 shared proteins among all three regions using STRING. Only protein-protein interactions verified by experimental data or databases were selected. The resulting complex network contained 1,157 nodes (proteins) and 16,627 edges (interactions). CytoCluster software was used to generate and rank subnetworks for visualization. The largest subnetwork was the SF3B1 network with 74 nodes and 2,313 edges. This group of proteins is involved in RNA splicing and translation. (TIFF) [file pone.0193250.s011.tiff]
