# Supplementary material for: Proteomic analysis of the human retina reveals region-specific susceptibilities to metabolic- and oxidative stress-related diseases
Source: PLoS One. 2018 Feb 21;13(2):e0193250. doi: 10.1371/journal.pone.0193250 (PMC5821407; doi:10.1371/journal.pone.0193250)
Supplement: S2 Table — Abundant proteins in our proteomics dataset were verified through comparison to a previously-published transcriptome dataset of the human retina (GSE40524). Expression data is reported in fragments per kilobase of exon per million fragments mapped (FPKM). A total of 66% identified proteins were also present in the RNA-seq dataset. (DOCX) [file pone.0193250.s013.docx]

**Supplemental Table 2. Comparison to previously-published human retina transcriptome:** Abundant proteins in our proteomics dataset were verified through comparison to a previously-published transcriptome dataset of the human retina (GSE40524). Expression data is reported in fragments per kilobase of exon per million fragments mapped (FPKM). A total of 66% identified proteins were also present in the RNA-seq dataset.

| **Protein** | | **Proteome** | | | **Transcriptome** |
| --- | --- | --- | --- | --- | --- |
|  |  | **Mean Spectral Count ± SD** | | | **Mean FPKM ± SD** |
| **Number** | **Name** | **Foveomacular** | **Juxta-macular** | **Periphery** | **Whole Retina** |
| 1 | PKM | 3439 ± 166 | 2542 ± 78 | 2598 ± 279 | 275.7 ± 83.5 |
| 2 | ENO1 | 3062 ± 634 | 2998 ± 331 | 4688 ± 1190 | 235.9 ± 119.3 |
| 3 | ENO2 | 2066 ±183 | 2070 ± 108 | 2282 ± 505 | 212.3 ± 254.3 |
| 4 | CKB | 1975 ± 90 | 1841 ± 111 | 2033 ± 109 | 114.7 ± 109.5 |
| 5 | H2BG | 1307 ± 54 | 1659 ± 215 | 1723 ± 133 | 1.9 ± 2.4 |
| 6 | H2B1D | 1272 ± 49 | 1612 ± 219 | 1650 ± 127 | 7.1 ± 3.1 |
| 7 | VIM | 3657 ± 396 | 3301 ± 297 | 2831 ± 146 | 0 ± 0 |
| 8 | TUBA | 3930 ± 376 | 3612 ± 325 | 3181 ± 85 | 0 ± 0 |
| 9 | TUBB | 3553 ± 161 | 3537 ± 206 | 3201 ± 117 | 0 ± 0 |
