# Supplementary material for: Proteomic analysis of the human retina reveals region-specific susceptibilities to metabolic- and oxidative stress-related diseases
Source: PLoS One. 2018 Feb 21;13(2):e0193250. doi: 10.1371/journal.pone.0193250 (PMC5821407; doi:10.1371/journal.pone.0193250)
Supplement: S3 Table — (DOCX) [file pone.0193250.s014.docx]

**Supplemental Table 3. Differentially-expressed proteins in the foveomacular retina.**

| **UniProt ID** | **Entry name** | **Protein names** |
| --- | --- | --- |
| Q9BPU6 | DPYL5_HUMAN | Dihydropyrimidinase-related protein 5 (DRP-5) (CRMP3-associated molecule) (CRAM) (Collapsin response mediator protein 5) (CRMP-5) (UNC33-like phosphoprotein 6) (ULIP-6) |
| Q13618 | CUL3_HUMAN | Cullin-3 (CUL-3) |
| P18669 | PGAM1_HUMAN | Phosphoglycerate mutase 1 (EC 5.4.2.11) (EC 5.4.2.4) (BPG-dependent PGAM 1) (Phosphoglycerate mutase isozyme B) (PGAM-B) |
| P13667 | PDIA4_HUMAN | Protein disulfide-isomerase A4 (EC 5.3.4.1) (Endoplasmic reticulum resident protein 70) (ER protein 70) (ERp70) (Endoplasmic reticulum resident protein 72) (ER protein 72) (ERp-72) (ERp72) |
| P12109 | CO6A1_HUMAN | Collagen alpha-1(VI) chain |
| Q86X55 | CARM1_HUMAN | Histone-arginine methyltransferase CARM1 (EC 2.1.1.319) (Coactivator-associated arginine methyltransferase 1) (Protein arginine N-methyltransferase 4) |
| P07814 | SYEP_HUMAN | Bifunctional glutamate/proline--tRNA ligase (Bifunctional aminoacyl-tRNA synthetase) (Cell proliferation-inducing gene 32 protein) (Glutamatyl-prolyl-tRNA synthetase) [Includes: Glutamate--tRNA ligase (EC 6.1.1.17) (Glutamyl-tRNA synthetase) (GluRS); Proline--tRNA ligase (EC 6.1.1.15) (Prolyl-tRNA synthetase)] |
| Q04637 | IF4G1_HUMAN | Eukaryotic translation initiation factor 4 gamma 1 (eIF-4-gamma 1) (eIF-4G 1) (eIF-4G1) (p220) |
| Q8WTQ7 | GRK7_HUMAN | Rhodopsin kinase (EC 2.7.11.14) (G protein-coupled receptor kinase 7) (G protein-coupled receptor kinase GRK7) |
| Q13616 | CUL1_HUMAN | Cullin-1 (CUL-1) |
| Q10567 | AP1B1_HUMAN | AP-1 complex subunit beta-1 (Adaptor protein complex AP-1 subunit beta-1) (Adaptor-related protein complex 1 subunit beta-1) (Beta-1-adaptin) (Beta-adaptin 1) (Clathrin assembly protein complex 1 beta large chain) (Golgi adaptor HA1/AP1 adaptin beta subunit) |
| P27816 | MAP4_HUMAN | Microtubule-associated protein 4 (MAP-4) |
| Q00059 | TFAM_HUMAN | Transcription factor A, mitochondrial (mtTFA) (Mitochondrial transcription factor 1) (MtTF1) (Transcription factor 6) (TCF-6) (Transcription factor 6-like 2) |
| Q05639 | EF1A2_HUMAN | Elongation factor 1-alpha 2 (EF-1-alpha-2) (Eukaryotic elongation factor 1 A-2) (eEF1A-2) (Statin-S1) |
| Q92616 | GCN1_HUMAN | eIF-2-alpha kinase activator GCN1 (GCN1 eIF-2-alpha kinase activator homolog) (GCN1-like protein 1) (General control of amino-acid synthesis 1-like protein 1) (Translational activator GCN1) (HsGCN1) |
| P16520 | GBB3_HUMAN | Guanine nucleotide-binding protein G(I)/G(S)/G(T) subunit beta-3 (Transducin beta chain 3) |
| P0DN77 | OPSG2_HUMAN | Medium-wave-sensitive opsin 2 (Green cone photoreceptor pigment) (Green-sensitive opsin) (GOP) (Opsin 1 cone pigments medium-wave-sensitive 2) |
| Q9UHY7 | ENOPH_HUMAN | Enolase-phosphatase E1 (EC 3.1.3.77) (2,3-diketo-5-methylthio-1-phosphopentane phosphatase) (MASA homolog) |
| Q9Y3I1 | FBX7_HUMAN | F-box only protein 7 |
| Q8IX01 | SUGP2_HUMAN | SURP and G-patch domain-containing protein 2 (Arginine/serine-rich-splicing factor 14) (Splicing factor, arginine/serine-rich 14) |
| A5YKK6 | CNOT1_HUMAN | CCR4-NOT transcription complex subunit 1 (CCR4-associated factor 1) (Negative regulator of transcription subunit 1 homolog) (NOT1H) (hNOT1) |
| P03999 | OPSB_HUMAN | Short-wave-sensitive opsin 1 (Blue cone photoreceptor pigment) (Blue-sensitive opsin) (BOP) |
| P47897 | SYQ_HUMAN | Glutamine--tRNA ligase (EC 6.1.1.18) (Glutaminyl-tRNA synthetase) (GlnRS) |
| P08779 | K1C16_HUMAN | Keratin, type I cytoskeletal 16 (Cytokeratin-16) (CK-16) (Keratin-16) (K16) |
| Q04695 | K1C17_HUMAN | Keratin, type I cytoskeletal 17 (39.1) (Cytokeratin-17) (CK-17) (Keratin-17) (K17) |
| Q9NSD9 | SYFB_HUMAN | Phenylalanine--tRNA ligase beta subunit (EC 6.1.1.20) (Phenylalanyl-tRNA synthetase beta subunit) (PheRS) |
| O60662 | KLH41_HUMAN | Kelch-like protein 41 (Kel-like protein 23) (Kelch repeat and BTB domain-containing protein 10) (Kelch-related protein 1) (Sarcosin) |
| Q9NZW5 | MPP6_HUMAN | MAGUK p55 subfamily member 6 (Veli-associated MAGUK 1) (VAM-1) |
| P63173 | RL38_HUMAN | 60S ribosomal protein L38 (Large ribosomal subunit protein eL38) |
| P11171 | 41_HUMAN | Protein 4.1 (P4.1) (4.1R) (Band 4.1) (EPB4.1) |
| Q13263 | TIF1B_HUMAN | Transcription intermediary factor 1-beta (TIF1-beta) (E3 SUMO-protein ligase TRIM28) (EC 2.3.2.27) (KRAB-associated protein 1) (KAP-1) (KRAB-interacting protein 1) (KRIP-1) (Nuclear corepressor KAP-1) (RING finger protein 96) (RING-type E3 ubiquitin transferase TIF1-beta) (Tripartite motif-containing protein 28) |
| Q7L775 | EPMIP_HUMAN | EPM2A-interacting protein 1 (Laforin-interacting protein) |
| P26641 | EF1G_HUMAN | Elongation factor 1-gamma (EF-1-gamma) (eEF-1B gamma) |
| P48444 | COPD_HUMAN | Coatomer subunit delta (Archain) (Delta-coat protein) (Delta-COP) |
| P00390 | GSHR_HUMAN | Glutathione reductase, mitochondrial (GR) (GRase) (EC 1.8.1.7) |
| Q99613 | EIF3C_HUMAN | Eukaryotic translation initiation factor 3 subunit C (eIF3c) (Eukaryotic translation initiation factor 3 subunit 8) (eIF3 p110) |
| D3DX48 | D3DX48_HUMAN | MAGUK p55 subfamily member 2 (Membrane protein, palmitoylated 2 (MAGUK p55 subfamily member 2), isoform CRA_b) |
| P14618 | KPYM_HUMAN | Pyruvate kinase PKM (EC 2.7.1.40) (Cytosolic thyroid hormone-binding protein) (CTHBP) (Opa-interacting protein 3) (OIP-3) (Pyruvate kinase 2/3) (Pyruvate kinase muscle isozyme) (Thyroid hormone-binding protein 1) (THBP1) (Tumor M2-PK) (p58) |
| Q14318 | FKBP8_HUMAN | Peptidyl-prolyl cis-trans isomerase FKBP8 (PPIase FKBP8) (EC 5.2.1.8) (38 kDa FK506-binding protein) (38 kDa FKBP) (FKBP-38) (hFKBP38) (FK506-binding protein 8) (FKBP-8) (FKBPR38) (Rotamase) |
| O76094 | SRP72_HUMAN | Signal recognition particle subunit SRP72 (SRP72) (Signal recognition particle 72 kDa protein) |
| P60033 | CD81_HUMAN | CD81 antigen (26 kDa cell surface protein TAPA-1) (Target of the antiproliferative antibody 1) (Tetraspanin-28) (Tspan-28) (CD antigen CD81) |
| P05455 | LA_HUMAN | Lupus La protein (La autoantigen) (La ribonucleoprotein) (Sjoegren syndrome type B antigen) (SS-B) |
| P46821 | MAP1B_HUMAN | Microtubule-associated protein 1B (MAP-1B) [Cleaved into: MAP1B heavy chain; MAP1 light chain LC1] |
| P62736 | ACTA_HUMAN | Actin, aortic smooth muscle (Alpha-actin-2) (Cell growth-inhibiting gene 46 protein) |
| P05091 | ALDH2_HUMAN | Aldehyde dehydrogenase, mitochondrial (EC 1.2.1.3) (ALDH class 2) (ALDH-E2) (ALDHI) |
| P53999 | TCP4_HUMAN | Activated RNA polymerase II transcriptional coactivator p15 (Positive cofactor 4) (PC4) (SUB1 homolog) (p14) |
| O75828 | CBR3_HUMAN | Carbonyl reductase [NADPH] 3 (EC 1.1.1.184) (NADPH-dependent carbonyl reductase 3) (Short chain dehydrogenase/reductase family 21C member 2) |
| Q8N1B4 | VPS52_HUMAN | Vacuolar protein sorting-associated protein 52 homolog (SAC2 suppressor of actin mutations 2-like protein) |
| Q15118 | PDK1_HUMAN | [Pyruvate dehydrogenase (acetyl-transferring)] kinase isozyme 1, mitochondrial (EC 2.7.11.2) (Pyruvate dehydrogenase kinase isoform 1) (PDH kinase 1) |
| P55209 | NP1L1_HUMAN | Nucleosome assembly protein 1-like 1 (NAP-1-related protein) (hNRP) |
| Q96L92 | SNX27_HUMAN | Sorting nexin-27 |
| P61247 | RS3A_HUMAN | 40S ribosomal protein S3a (Small ribosomal subunit protein eS1) (v-fos transformation effector protein) (Fte-1) |
| A0A075B6E2 | A0A075B6E2_HUMAN | 40S ribosomal protein S19 |
| P05165 | PCCA_HUMAN | Propionyl-CoA carboxylase alpha chain, mitochondrial (PCCase subunit alpha) (EC 6.4.1.3) (Propanoyl-CoA:carbon dioxide ligase subunit alpha) |
| P05067 | A4_HUMAN | Amyloid beta A4 protein (ABPP) (APPI) (APP) (Alzheimer disease amyloid protein) (Amyloid precursor protein) (Beta-amyloid precursor protein) (Cerebral vascular amyloid peptide) (CVAP) (PreA4) (Protease nexin-II) (PN-II) [Cleaved into: N-APP; Soluble APP-alpha (S-APP-alpha); Soluble APP-beta (S-APP-beta); C99; Beta-amyloid protein 42 (Beta-APP42); Beta-amyloid protein 40 (Beta-APP40); C83; P3(42); P3(40); C80; Gamma-secretase C-terminal fragment 59 (Amyloid intracellular domain 59) (AICD-59) (AID(59)) (Gamma-CTF(59)); Gamma-secretase C-terminal fragment 57 (Amyloid intracellular domain 57) (AICD-57) (AID(57)) (Gamma-CTF(57)); Gamma-secretase C-terminal fragment 50 (Amyloid intracellular domain 50) (AICD-50) (AID(50)) (Gamma-CTF(50)); C31] |
| P59190 | RAB15_HUMAN | Ras-related protein Rab-15 |
| Q53GS9 | SNUT2_HUMAN | U4/U6.U5 tri-snRNP-associated protein 2 (Inactive ubiquitin-specific peptidase 39) (SAD1 homolog) (U4/U6.U5 tri-snRNP-associated 65 kDa protein) (65K) |
| A8MWX0 | A8MWX0_HUMAN | Receptor expression-enhancing protein (Fragment) |
| Q14152 | EIF3A_HUMAN | Eukaryotic translation initiation factor 3 subunit A (eIF3a) (Eukaryotic translation initiation factor 3 subunit 10) (eIF-3-theta) (eIF3 p167) (eIF3 p180) (eIF3 p185) |
| Q12905 | ILF2_HUMAN | Interleukin enhancer-binding factor 2 (Nuclear factor of activated T-cells 45 kDa) |
| Q9H4A4 | AMPB_HUMAN | Aminopeptidase B (AP-B) (EC 3.4.11.6) (Arginine aminopeptidase) (Arginyl aminopeptidase) |
| Q9GZP4 | PITH1_HUMAN | PITH domain-containing protein 1 |
| O75935 | DCTN3_HUMAN | Dynactin subunit 3 (Dynactin complex subunit 22 kDa subunit) (p22) |
| Q71UM5 | RS27L_HUMAN | 40S ribosomal protein S27-like (Small ribosomal subunit protein eS27-like) |
| P61160 | ARP2_HUMAN | Actin-related protein 2 (Actin-like protein 2) |
| P41252 | SYIC_HUMAN | Isoleucine--tRNA ligase, cytoplasmic (EC 6.1.1.5) (Isoleucyl-tRNA synthetase) (IRS) (IleRS) |
| P11217 | PYGM_HUMAN | Glycogen phosphorylase, muscle form (EC 2.4.1.1) (Myophosphorylase) |
| P51648 | AL3A2_HUMAN | Fatty aldehyde dehydrogenase (EC 1.2.1.3) (Aldehyde dehydrogenase 10) (Aldehyde dehydrogenase family 3 member A2) (Microsomal aldehyde dehydrogenase) |
| O43237 | DC1L2_HUMAN | Cytoplasmic dynein 1 light intermediate chain 2 (Dynein light intermediate chain 2, cytosolic) (LIC-2) (LIC53/55) |
| P53680 | AP2S1_HUMAN | AP-2 complex subunit sigma (Adaptor protein complex AP-2 subunit sigma) (Adaptor-related protein complex 2 subunit sigma) (Clathrin assembly protein 2 sigma small chain) (Clathrin coat assembly protein AP17) (Clathrin coat-associated protein AP17) (HA2 17 kDa subunit) (Plasma membrane adaptor AP-2 17 kDa protein) (Sigma2-adaptin) |
| P23634 | AT2B4_HUMAN | Plasma membrane calcium-transporting ATPase 4 (PMCA4) (EC 3.6.3.8) (Matrix-remodeling-associated protein 1) (Plasma membrane calcium ATPase isoform 4) (Plasma membrane calcium pump isoform 4) |
| Q96CN7 | ISOC1_HUMAN | Isochorismatase domain-containing protein 1 |
| P50914 | RL14_HUMAN | 60S ribosomal protein L14 (CAG-ISL 7) (Large ribosomal subunit protein eL14) |
| P62249 | RS16_HUMAN | 40S ribosomal protein S16 (Small ribosomal subunit protein uS9) |
| O43776 | SYNC_HUMAN | Asparagine--tRNA ligase, cytoplasmic (EC 6.1.1.22) (Asparaginyl-tRNA synthetase) (AsnRS) |
| P42704 | LPPRC_HUMAN | Leucine-rich PPR motif-containing protein, mitochondrial (130 kDa leucine-rich protein) (LRP 130) (GP130) |
| Q7KZF4 | SND1_HUMAN | Staphylococcal nuclease domain-containing protein 1 (100 kDa coactivator) (EBNA2 coactivator p100) (Tudor domain-containing protein 11) (p100 co-activator) |
| Q9H115 | SNAB_HUMAN | Beta-soluble NSF attachment protein (SNAP-beta) (N-ethylmaleimide-sensitive factor attachment protein beta) |
| Q12906 | ILF3_HUMAN | Interleukin enhancer-binding factor 3 (Double-stranded RNA-binding protein 76) (DRBP76) (M-phase phosphoprotein 4) (MPP4) (Nuclear factor associated with dsRNA) (NFAR) (Nuclear factor of activated T-cells 90 kDa) (NF-AT-90) (Translational control protein 80) (TCP80) |
| P51149 | RAB7A_HUMAN | Ras-related protein Rab-7a |
| Q14764 | MVP_HUMAN | Major vault protein (MVP) (Lung resistance-related protein) |
| Q9UNS2 | CSN3_HUMAN | COP9 signalosome complex subunit 3 (SGN3) (Signalosome subunit 3) (JAB1-containing signalosome subunit 3) |
| Q96QK1 | VPS35_HUMAN | Vacuolar protein sorting-associated protein 35 (hVPS35) (Maternal-embryonic 3) (Vesicle protein sorting 35) |
| P62266 | RS23_HUMAN | 40S ribosomal protein S23 (Small ribosomal subunit protein uS12) |
| P26640 | SYVC_HUMAN | Valine--tRNA ligase (EC 6.1.1.9) (Protein G7a) (Valyl-tRNA synthetase) (ValRS) |
| Q01813 | PFKAP_HUMAN | ATP-dependent 6-phosphofructokinase, platelet type (ATP-PFK) (PFK-P) (EC 2.7.1.11) (6-phosphofructokinase type C) (Phosphofructo-1-kinase isozyme C) (PFK-C) (Phosphohexokinase) |
| P54136 | SYRC_HUMAN | Arginine--tRNA ligase, cytoplasmic (EC 6.1.1.19) (Arginyl-tRNA synthetase) (ArgRS) |
| Q08945 | SSRP1_HUMAN | FACT complex subunit SSRP1 (Chromatin-specific transcription elongation factor 80 kDa subunit) (Facilitates chromatin transcription complex 80 kDa subunit) (FACT 80 kDa subunit) (FACTp80) (Facilitates chromatin transcription complex subunit SSRP1) (Recombination signal sequence recognition protein 1) (Structure-specific recognition protein 1) (hSSRP1) (T160) |
| Q14980 | NUMA1_HUMAN | Nuclear mitotic apparatus protein 1 (Nuclear matrix protein-22) (NMP-22) (Nuclear mitotic apparatus protein) (NuMA protein) (SP-H antigen) |
| P09417 | DHPR_HUMAN | Dihydropteridine reductase (EC 1.5.1.34) (HDHPR) (Quinoid dihydropteridine reductase) (Short chain dehydrogenase/reductase family 33C member 1) |
| Q7L5N1 | CSN6_HUMAN | COP9 signalosome complex subunit 6 (SGN6) (Signalosome subunit 6) (JAB1-containing signalosome subunit 6) (MOV34 homolog) (Vpr-interacting protein) (hVIP) |
| Q9P2J5 | SYLC_HUMAN | Leucine--tRNA ligase, cytoplasmic (EC 6.1.1.4) (Leucyl-tRNA synthetase) (LeuRS) |
| P11216 | PYGB_HUMAN | Glycogen phosphorylase, brain form (EC 2.4.1.1) |
| Q08380 | LG3BP_HUMAN | Galectin-3-binding protein (Basement membrane autoantigen p105) (Lectin galactoside-binding soluble 3-binding protein) (Mac-2-binding protein) (MAC2BP) (Mac-2 BP) (Tumor-associated antigen 90K) |
| Q14576 | ELAV3_HUMAN | ELAV-like protein 3 (Hu-antigen C) (HuC) (Paraneoplastic cerebellar degeneration-associated antigen) (Paraneoplastic limbic encephalitis antigen 21) |
| P46778 | RL21_HUMAN | 60S ribosomal protein L21 (Large ribosomal subunit protein eL21) |
| P05388 | RLA0_HUMAN | 60S acidic ribosomal protein P0 (60S ribosomal protein L10E) (Large ribosomal subunit protein uL10) |
| P62191 | PRS4_HUMAN | 26S proteasome regulatory subunit 4 (P26s4) (26S proteasome AAA-ATPase subunit RPT2) (Proteasome 26S subunit ATPase 1) |
| P56192 | SYMC_HUMAN | Methionine--tRNA ligase, cytoplasmic (EC 6.1.1.10) (Methionyl-tRNA synthetase) (MetRS) |
| Q9Y3U8 | RL36_HUMAN | 60S ribosomal protein L36 (Large ribosomal subunit protein eL36) |
| Q9Y6G9 | DC1L1_HUMAN | Cytoplasmic dynein 1 light intermediate chain 1 (LIC1) (Dynein light chain A) (DLC-A) (Dynein light intermediate chain 1, cytosolic) |
| P19525 | E2AK2_HUMAN | Interferon-induced, double-stranded RNA-activated protein kinase (EC 2.7.11.1) (Eukaryotic translation initiation factor 2-alpha kinase 2) (eIF-2A protein kinase 2) (Interferon-inducible RNA-dependent protein kinase) (P1/eIF-2A protein kinase) (Protein kinase RNA-activated) (PKR) (Protein kinase R) (Tyrosine-protein kinase EIF2AK2) (EC 2.7.10.2) (p68 kinase) |
| P32929 | CGL_HUMAN | Cystathionine gamma-lyase (EC 4.4.1.1) (Cysteine-protein sulfhydrase) (Gamma-cystathionase) |
| Q13510 | ASAH1_HUMAN | Acid ceramidase (AC) (ACDase) (Acid CDase) (EC 3.5.1.23) (Acylsphingosine deacylase) (N-acylsphingosine amidohydrolase) (Putative 32 kDa heart protein) (PHP32) [Cleaved into: Acid ceramidase subunit alpha; Acid ceramidase subunit beta] |
| Q5JR95 | Q5JR95_HUMAN | 40S ribosomal protein S8 |
| P62753 | RS6_HUMAN | 40S ribosomal protein S6 (Phosphoprotein NP33) (Small ribosomal subunit protein eS6) |
| P62241 | RS8_HUMAN | 40S ribosomal protein S8 (Small ribosomal subunit protein eS8) |
| O43143 | DHX15_HUMAN | Pre-mRNA-splicing factor ATP-dependent RNA helicase DHX15 (EC 3.6.4.13) (ATP-dependent RNA helicase #46) (DEAH box protein 15) |
| Q9NZN4 | EHD2_HUMAN | EH domain-containing protein 2 (PAST homolog 2) |
| P61586 | RHOA_HUMAN | Transforming protein RhoA (Rho cDNA clone 12) (h12) |
| P27635 | RL10_HUMAN | 60S ribosomal protein L10 (Laminin receptor homolog) (Large ribosomal subunit protein uL16) (Protein QM) (Tumor suppressor QM) |
| Q9UHB9 | SRP68_HUMAN | Signal recognition particle subunit SRP68 (SRP68) (Signal recognition particle 68 kDa protein) |
| P36575 | ARRC_HUMAN | Arrestin-C (Cone arrestin) (C-arrestin) (cArr) (Retinal cone arrestin-3) (X-arrestin) |
| P13639 | EF2_HUMAN | Elongation factor 2 (EF-2) |
| Q01433 | AMPD2_HUMAN | AMP deaminase 2 (EC 3.5.4.6) (AMP deaminase isoform L) |
| P50991 | TCPD_HUMAN | T-complex protein 1 subunit delta (TCP-1-delta) (CCT-delta) (Stimulator of TAR RNA-binding) |
| P07305 | H10_HUMAN | Histone H1.0 (Histone H1') (Histone H1(0)) [Cleaved into: Histone H1.0, N-terminally processed] |
| P30038 | AL4A1_HUMAN | Delta-1-pyrroline-5-carboxylate dehydrogenase, mitochondrial (P5C dehydrogenase) (EC 1.2.1.88) (Aldehyde dehydrogenase family 4 member A1) (L-glutamate gamma-semialdehyde dehydrogenase) |
| P22033 | MUTA_HUMAN | Methylmalonyl-CoA mutase, mitochondrial (MCM) (EC 5.4.99.2) (Methylmalonyl-CoA isomerase) |
| Q9GZT3 | SLIRP_HUMAN | SRA stem-loop-interacting RNA-binding protein, mitochondrial |
| Q13596 | SNX1_HUMAN | Sorting nexin-1 |
| Q9Y285 | SYFA_HUMAN | Phenylalanine--tRNA ligase alpha subunit (EC 6.1.1.20) (CML33) (Phenylalanyl-tRNA synthetase alpha subunit) (PheRS) |
| P17987 | TCPA_HUMAN | T-complex protein 1 subunit alpha (TCP-1-alpha) (CCT-alpha) |
| P30050 | RL12_HUMAN | 60S ribosomal protein L12 (Large ribosomal subunit protein uL11) |
| Q9H9Q2 | CSN7B_HUMAN | COP9 signalosome complex subunit 7b (SGN7b) (Signalosome subunit 7b) (JAB1-containing signalosome subunit 7b) |
| P55011 | S12A2_HUMAN | Solute carrier family 12 member 2 (Basolateral Na-K-Cl symporter) (Bumetanide-sensitive sodium-(potassium)-chloride cotransporter 1) |
| Q16762 | THTR_HUMAN | Thiosulfate sulfurtransferase (EC 2.8.1.1) (Rhodanese) |
| O15294 | OGT1_HUMAN | UDP-N-acetylglucosamine--peptide N-acetylglucosaminyltransferase 110 kDa subunit (EC 2.4.1.255) (O-GlcNAc transferase subunit p110) (O-linked N-acetylglucosamine transferase 110 kDa subunit) (OGT) |
| A0A0A0MSA4 | A0A0A0MSA4_HUMAN | Band 4.1-like protein 3 |
| A0A0A0MRA8 | A0A0A0MRA8_HUMAN | Band 4.1-like protein 3 |
| P17858 | PFKAL_HUMAN | ATP-dependent 6-phosphofructokinase, liver type (ATP-PFK) (PFK-L) (EC 2.7.1.11) (6-phosphofructokinase type B) (Phosphofructo-1-kinase isozyme B) (PFK-B) (Phosphohexokinase) |
| Q9P035 | HACD3_HUMAN | Very-long-chain (3R)-3-hydroxyacyl-CoA dehydratase 3 (EC 4.2.1.134) (3-hydroxyacyl-CoA dehydratase 3) (HACD3) (Butyrate-induced protein 1) (B-ind1) (hB-ind1) (Protein-tyrosine phosphatase-like A domain-containing protein 1) |
| Q9NVJ2 | ARL8B_HUMAN | ADP-ribosylation factor-like protein 8B (ADP-ribosylation factor-like protein 10C) (Novel small G protein indispensable for equal chromosome segregation 1) |
| Q15067 | ACOX1_HUMAN | Peroxisomal acyl-coenzyme A oxidase 1 (AOX) (EC 1.3.3.6) (Palmitoyl-CoA oxidase) (Straight-chain acyl-CoA oxidase) (SCOX) |
| P46781 | RS9_HUMAN | 40S ribosomal protein S9 (Small ribosomal subunit protein uS4) |
| Q9UPX0 | TUTLB_HUMAN | Protein turtle homolog B (Immunoglobulin superfamily member 9B) (IgSF9B) |
| Q16799 | RTN1_HUMAN | Reticulon-1 (Neuroendocrine-specific protein) |
| P62330 | ARF6_HUMAN | ADP-ribosylation factor 6 |
| Q9UBB6 | NCDN_HUMAN | Neurochondrin |
| Q9UM22 | EPDR1_HUMAN | Mammalian ependymin-related protein 1 (MERP-1) (Upregulated in colorectal cancer gene 1 protein) |
| P23396 | RS3_HUMAN | 40S ribosomal protein S3 (EC 4.2.99.18) (Small ribosomal subunit protein uS3) |
| P59998 | ARPC4_HUMAN | Actin-related protein 2/3 complex subunit 4 (Arp2/3 complex 20 kDa subunit) (p20-ARC) |
| O75964 | ATP5L_HUMAN | ATP synthase subunit g, mitochondrial (ATPase subunit g) |
| Q9UQB3 | CTND2_HUMAN | Catenin delta-2 (Delta-catenin) (GT24) (Neural plakophilin-related ARM-repeat protein) (NPRAP) (Neurojungin) |
| Q9NQC3 | RTN4_HUMAN | Reticulon-4 (Foocen) (Neurite outgrowth inhibitor) (Nogo protein) (Neuroendocrine-specific protein) (NSP) (Neuroendocrine-specific protein C homolog) (RTN-x) (Reticulon-5) |
| P51674 | GPM6A_HUMAN | Neuronal membrane glycoprotein M6-a (M6a) |
| Q9UBC2 | EP15R_HUMAN | Epidermal growth factor receptor substrate 15-like 1 (Eps15-related protein) (Eps15R) |
| Q13554 | KCC2B_HUMAN | Calcium/calmodulin-dependent protein kinase type II subunit beta (CaM kinase II subunit beta) (CaMK-II subunit beta) (EC 2.7.11.17) |
| P08247 | SYPH_HUMAN | Synaptophysin (Major synaptic vesicle protein p38) |
| Q8N9R8 | SCAI_HUMAN | Protein SCAI (Suppressor of cancer cell invasion protein) |
| Q8N568 | DCLK2_HUMAN | Serine/threonine-protein kinase DCLK2 (EC 2.7.11.1) (CaMK-like CREB regulatory kinase 2) (CL2) (CLICK-II) (CLICK2) (Doublecortin domain-containing protein 3B) (Doublecortin-like and CAM kinase-like 2) (Doublecortin-like kinase 2) |
| O95674 | CDS2_HUMAN | Phosphatidate cytidylyltransferase 2 (EC 2.7.7.41) (CDP-DAG synthase 2) (CDP-DG synthase 2) (CDP-diacylglycerol synthase 2) (CDS 2) (CDP-diglyceride pyrophosphorylase 2) (CDP-diglyceride synthase 2) (CTP:phosphatidate cytidylyltransferase 2) |
| O43747 | AP1G1_HUMAN | AP-1 complex subunit gamma-1 (Adaptor protein complex AP-1 subunit gamma-1) (Adaptor-related protein complex 1 subunit gamma-1) (Clathrin assembly protein complex 1 gamma-1 large chain) (Gamma1-adaptin) (Golgi adaptor HA1/AP1 adaptin subunit gamma-1) |
| O75436 | VP26A_HUMAN | Vacuolar protein sorting-associated protein 26A (Vesicle protein sorting 26A) (hVPS26) |
| P20336 | RAB3A_HUMAN | Ras-related protein Rab-3A |
| O15537 | XLRS1_HUMAN | Retinoschisin (X-linked juvenile retinoschisis protein) |
| P35268 | RL22_HUMAN | 60S ribosomal protein L22 (EBER-associated protein) (EAP) (Epstein-Barr virus small RNA-associated protein) (Heparin-binding protein HBp15) (Large ribosomal subunit protein eL22) |
| Q8TC07 | TBC15_HUMAN | TBC1 domain family member 15 (GTPase-activating protein RAB7) (GAP for RAB7) (Rab7-GAP) |
| Q9H0U4 | RAB1B_HUMAN | Ras-related protein Rab-1B |
| P61106 | RAB14_HUMAN | Ras-related protein Rab-14 |
| Q8WUK0 | PTPM1_HUMAN | Phosphatidylglycerophosphatase and protein-tyrosine phosphatase 1 (EC 3.1.3.27) (PTEN-like phosphatase) (Phosphoinositide lipid phosphatase) (Protein-tyrosine phosphatase mitochondrial 1) (EC 3.1.3.16) (EC 3.1.3.48) |
| Q08211 | DHX9_HUMAN | ATP-dependent RNA helicase A (RHA) (EC 3.6.4.13) (DEAH box protein 9) (Leukophysin) (LKP) (Nuclear DNA helicase II) (NDH II) |
| P56134 | ATPK_HUMAN | ATP synthase subunit f, mitochondrial |
| P51571 | SSRD_HUMAN | Translocon-associated protein subunit delta (TRAP-delta) (Signal sequence receptor subunit delta) (SSR-delta) |
| Q15386 | UBE3C_HUMAN | Ubiquitin-protein ligase E3C (EC 2.3.2.26) (HECT-type ubiquitin transferase E3C) (HectH2) |
| Q9Y512 | SAM50_HUMAN | Sorting and assembly machinery component 50 homolog (Transformation-related gene 3 protein) (TRG-3) |
| P51659 | DHB4_HUMAN | Peroxisomal multifunctional enzyme type 2 (MFE-2) (17-beta-hydroxysteroid dehydrogenase 4) (17-beta-HSD 4) (D-bifunctional protein) (DBP) (Multifunctional protein 2) (MPF-2) (Short chain dehydrogenase/reductase family 8C member 1) [Cleaved into: (3R)-hydroxyacyl-CoA dehydrogenase (EC 1.1.1.n12); Enoyl-CoA hydratase 2 (EC 4.2.1.107) (EC 4.2.1.119) (3-alpha,7-alpha,12-alpha-trihydroxy-5-beta-cholest-24-enoyl-CoA hydratase)] |
| P61158 | ARP3_HUMAN | Actin-related protein 3 (Actin-like protein 3) |
| P03886 | NU1M_HUMAN | NADH-ubiquinone oxidoreductase chain 1 (EC 1.6.5.3) (NADH dehydrogenase subunit 1) |
| Q93009 | UBP7_HUMAN | Ubiquitin carboxyl-terminal hydrolase 7 (EC 3.4.19.12) (Deubiquitinating enzyme 7) (Herpesvirus-associated ubiquitin-specific protease) (Ubiquitin thioesterase 7) (Ubiquitin-specific-processing protease 7) |
| Q8TBG9 | SYNPR_HUMAN | Synaptoporin |
| Q14008 | CKAP5_HUMAN | Cytoskeleton-associated protein 5 (Colonic and hepatic tumor overexpressed gene protein) (Ch-TOG) |
| O95716 | RAB3D_HUMAN | Ras-related protein Rab-3D |
| P22087 | FBRL_HUMAN | rRNA 2'-O-methyltransferase fibrillarin (EC 2.1.1.-) (34 kDa nucleolar scleroderma antigen) (Histone-glutamine methyltransferase) |
| Q92930 | RAB8B_HUMAN | Ras-related protein Rab-8B |
| P61006 | RAB8A_HUMAN | Ras-related protein Rab-8A (Oncogene c-mel) |
| Q9UDW1 | QCR9_HUMAN | Cytochrome b-c1 complex subunit 9 (Complex III subunit 9) (Complex III subunit X) (Cytochrome c1 non-heme 7 kDa protein) (Ubiquinol-cytochrome c reductase complex 7.2 kDa protein) |
| P61019 | RAB2A_HUMAN | Ras-related protein Rab-2A |
| P62820 | RAB1A_HUMAN | Ras-related protein Rab-1A (YPT1-related protein) |
| P35606 | COPB2_HUMAN | Coatomer subunit beta' (Beta'-coat protein) (Beta'-COP) (p102) |
| P78527 | PRKDC_HUMAN | DNA-dependent protein kinase catalytic subunit (DNA-PK catalytic subunit) (DNA-PKcs) (EC 2.7.11.1) (DNPK1) (p460) |
| P27694 | RFA1_HUMAN | Replication protein A 70 kDa DNA-binding subunit (RP-A p70) (Replication factor A protein 1) (RF-A protein 1) (Single-stranded DNA-binding protein) [Cleaved into: Replication protein A 70 kDa DNA-binding subunit, N-terminally processed] |
| Q9UBT2 | SAE2_HUMAN | SUMO-activating enzyme subunit 2 (EC 6.3.2.-) (Anthracycline-associated resistance ARX) (Ubiquitin-like 1-activating enzyme E1B) (Ubiquitin-like modifier-activating enzyme 2) |
| Q05193 | DYN1_HUMAN | Dynamin-1 (EC 3.6.5.5) |
| Q9NY65 | TBA8_HUMAN | Tubulin alpha-8 chain (Alpha-tubulin 8) (Tubulin alpha chain-like 2) |
| P46779 | RL28_HUMAN | 60S ribosomal protein L28 (Large ribosomal subunit protein eL28) |
| Q9Y2Z4 | SYYM_HUMAN | Tyrosine--tRNA ligase, mitochondrial (EC 6.1.1.1) (Tyrosyl-tRNA synthetase) (TyrRS) |
| P68366 | TBA4A_HUMAN | Tubulin alpha-4A chain (Alpha-tubulin 1) (Testis-specific alpha-tubulin) (Tubulin H2-alpha) (Tubulin alpha-1 chain) |
| Q9BY67 | CADM1_HUMAN | Cell adhesion molecule 1 (Immunoglobulin superfamily member 4) (IgSF4) (Nectin-like protein 2) (NECL-2) (Spermatogenic immunoglobulin superfamily) (SgIgSF) (Synaptic cell adhesion molecule) (SynCAM) (Tumor suppressor in lung cancer 1) (TSLC-1) |
| Q71U36 | TBA1A_HUMAN | Tubulin alpha-1A chain (Alpha-tubulin 3) (Tubulin B-alpha-1) (Tubulin alpha-3 chain) [Cleaved into: Detyrosinated tubulin alpha-1A chain] |
| Q5H9R7 | PP6R3_HUMAN | Serine/threonine-protein phosphatase 6 regulatory subunit 3 (SAPS domain family member 3) (Sporulation-induced transcript 4-associated protein SAPL) |
| Q2M2I8 | AAK1_HUMAN | AP2-associated protein kinase 1 (EC 2.7.11.1) (Adaptor-associated kinase 1) |
| Q9H2X9 | S12A5_HUMAN | Solute carrier family 12 member 5 (Electroneutral potassium-chloride cotransporter 2) (K-Cl cotransporter 2) (hKCC2) (Neuronal K-Cl cotransporter) |
| Q9NZR1 | TMOD2_HUMAN | Tropomodulin-2 (Neuronal tropomodulin) (N-Tmod) |
| H7C279 | H7C279_HUMAN | Serine/threonine-protein kinase 25 (Fragment) |
| Q9UGV2 | NDRG3_HUMAN | Protein NDRG3 (N-myc downstream-regulated gene 3 protein) |
| Q00610 | CLH1_HUMAN | Clathrin heavy chain 1 (Clathrin heavy chain on chromosome 17) (CLH-17) |
| O95292 | VAPB_HUMAN | Vesicle-associated membrane protein-associated protein B/C (VAMP-B/VAMP-C) (VAMP-associated protein B/C) (VAP-B/VAP-C) |
| P54709 | AT1B3_HUMAN | Sodium/potassium-transporting ATPase subunit beta-3 (Sodium/potassium-dependent ATPase subunit beta-3) (ATPB-3) (CD antigen CD298) |
| Q15813 | TBCE_HUMAN | Tubulin-specific chaperone E (Tubulin-folding cofactor E) |
| J3KN01 | J3KN01_HUMAN | Afadin |
| Q92900 | RENT1_HUMAN | Regulator of nonsense transcripts 1 (EC 3.6.4.-) (ATP-dependent helicase RENT1) (Nonsense mRNA reducing factor 1) (NORF1) (Up-frameshift suppressor 1 homolog) (hUpf1) |
| P78357 | CNTP1_HUMAN | Contactin-associated protein 1 (Caspr) (Caspr1) (Neurexin IV) (Neurexin-4) (p190) |
| P08195 | 4F2_HUMAN | 4F2 cell-surface antigen heavy chain (4F2hc) (4F2 heavy chain antigen) (Lymphocyte activation antigen 4F2 large subunit) (Solute carrier family 3 member 2) (CD antigen CD98) |
| Q9UNX3 | RL26L_HUMAN | 60S ribosomal protein L26-like 1 (Large ribosomal subunit protein uL24-like 1) |
| P49748 | ACADV_HUMAN | Very long-chain specific acyl-CoA dehydrogenase, mitochondrial (VLCAD) (EC 1.3.8.9) |
| Q7Z460 | CLAP1_HUMAN | CLIP-associating protein 1 (Cytoplasmic linker-associated protein 1) (Multiple asters homolog 1) (Protein Orbit homolog 1) (hOrbit1) |
| A0A0A0MR39 | A0A0A0MR39_HUMAN | Myelin expression factor 2 (Myelin expression factor 2, isoform CRA_a) |
| Q14320 | FA50A_HUMAN | Protein FAM50A (Protein HXC-26) (Protein XAP-5) |
| Q9Y376 | CAB39_HUMAN | Calcium-binding protein 39 (MO25alpha) (Protein Mo25) |
| Q14151 | SAFB2_HUMAN | Scaffold attachment factor B2 (SAF-B2) |
| P62906 | RL10A_HUMAN | 60S ribosomal protein L10a (CSA-19) (Large ribosomal subunit protein uL1) (Neural precursor cell expressed developmentally down-regulated protein 6) (NEDD-6) |
| P54687 | BCAT1_HUMAN | Branched-chain-amino-acid aminotransferase, cytosolic (BCAT(c)) (EC 2.6.1.42) (Protein ECA39) |
| J3KMX5 | J3KMX5_HUMAN | 40S ribosomal protein S13 |
| Q8IZ81 | ELMD2_HUMAN | ELMO domain-containing protein 2 |
| O95563 | MPC2_HUMAN | Mitochondrial pyruvate carrier 2 (Brain protein 44) |
| Q93008 | USP9X_HUMAN | Probable ubiquitin carboxyl-terminal hydrolase FAF-X (EC 3.4.19.12) (Deubiquitinating enzyme FAF-X) (Fat facets in mammals) (hFAM) (Fat facets protein-related, X-linked) (Ubiquitin thioesterase FAF-X) (Ubiquitin-specific protease 9, X chromosome) (Ubiquitin-specific-processing protease FAF-X) |
| O95573 | ACSL3_HUMAN | Long-chain-fatty-acid--CoA ligase 3 (EC 6.2.1.3) (Long-chain acyl-CoA synthetase 3) (LACS 3) |
| P30049 | ATPD_HUMAN | ATP synthase subunit delta, mitochondrial (F-ATPase delta subunit) |
| Q96PE3 | INP4A_HUMAN | Type I inositol 3,4-bisphosphate 4-phosphatase (EC 3.1.3.66) (Inositol polyphosphate 4-phosphatase type I) |
| P62760 | VISL1_HUMAN | Visinin-like protein 1 (VILIP) (VLP-1) (Hippocalcin-like protein 3) (HLP3) |
| Q9Y678 | COPG1_HUMAN | Coatomer subunit gamma-1 (Gamma-1-coat protein) (Gamma-1-COP) |
| P08238 | HS90B_HUMAN | Heat shock protein HSP 90-beta (HSP 90) (Heat shock 84 kDa) (HSP 84) (HSP84) |
| Q02543 | RL18A_HUMAN | 60S ribosomal protein L18a (Large ribosomal subunit protein eL20) |
| O95373 | IPO7_HUMAN | Importin-7 (Imp7) (Ran-binding protein 7) (RanBP7) |
| Q92896 | GSLG1_HUMAN | Golgi apparatus protein 1 (CFR-1) (Cysteine-rich fibroblast growth factor receptor) (E-selectin ligand 1) (ESL-1) (Golgi sialoglycoprotein MG-160) |
| Q14919 | NC2A_HUMAN | Dr1-associated corepressor (Dr1-associated protein 1) (Negative cofactor 2-alpha) (NC2-alpha) |
| Q96HU8 | DIRA2_HUMAN | GTP-binding protein Di-Ras2 (Distinct subgroup of the Ras family member 2) |
| Q9UI12 | VATH_HUMAN | V-type proton ATPase subunit H (V-ATPase subunit H) (Nef-binding protein 1) (NBP1) (Protein VMA13 homolog) (V-ATPase 50/57 kDa subunits) (Vacuolar proton pump subunit H) (Vacuolar proton pump subunit SFD) |
| C9JD32 | C9JD32_HUMAN | 60S ribosomal protein L23 (Fragment) |
| Q9ULD0 | OGDHL_HUMAN | 2-oxoglutarate dehydrogenase-like, mitochondrial (EC 1.2.4.-) (2-oxoglutarate dehydrogenase complex component E1-like) (OGDC-E1-like) (Alpha-ketoglutarate dehydrogenase-like) |
| O75390 | CISY_HUMAN | Citrate synthase, mitochondrial (EC 2.3.3.1) (Citrate (Si)-synthase) |
| Q9UJS0 | CMC2_HUMAN | Calcium-binding mitochondrial carrier protein Aralar2 (Citrin) (Mitochondrial aspartate glutamate carrier 2) (Solute carrier family 25 member 13) |
| P36542 | ATPG_HUMAN | ATP synthase subunit gamma, mitochondrial (F-ATPase gamma subunit) |
| P53618 | COPB_HUMAN | Coatomer subunit beta (Beta-coat protein) (Beta-COP) |
| J3KPX7 | J3KPX7_HUMAN | Prohibitin-2 |
| O94925 | GLSK_HUMAN | Glutaminase kidney isoform, mitochondrial (GLS) (EC 3.5.1.2) (K-glutaminase) (L-glutamine amidohydrolase) |
| P02788 | TRFL_HUMAN | Lactotransferrin (Lactoferrin) (EC 3.4.21.-) (Growth-inhibiting protein 12) (Talalactoferrin) [Cleaved into: Lactoferricin-H (Lfcin-H); Kaliocin-1; Lactoferroxin-A; Lactoferroxin-B; Lactoferroxin-C] |
| Q9UNW9 | NOVA2_HUMAN | RNA-binding protein Nova-2 (Astrocytic NOVA1-like RNA-binding protein) (Neuro-oncological ventral antigen 2) |
| Q96EY1 | DNJA3_HUMAN | DnaJ homolog subfamily A member 3, mitochondrial (DnaJ protein Tid-1) (hTid-1) (Hepatocellular carcinoma-associated antigen 57) (Tumorous imaginal discs protein Tid56 homolog) |
| Q9H0D6 | XRN2_HUMAN | 5'-3' exoribonuclease 2 (EC 3.1.13.-) (DHM1-like protein) (DHP protein) |
| P11498 | PYC_HUMAN | Pyruvate carboxylase, mitochondrial (EC 6.4.1.1) (Pyruvic carboxylase) (PCB) |
| P53367 | ARFP1_HUMAN | Arfaptin-1 (ADP-ribosylation factor-interacting protein 1) |
| P60709 | ACTB_HUMAN | Actin, cytoplasmic 1 (Beta-actin) [Cleaved into: Actin, cytoplasmic 1, N-terminally processed] |
| P63261 | ACTG_HUMAN | Actin, cytoplasmic 2 (Gamma-actin) [Cleaved into: Actin, cytoplasmic 2, N-terminally processed] |
| P10515 | ODP2_HUMAN | Dihydrolipoyllysine-residue acetyltransferase component of pyruvate dehydrogenase complex, mitochondrial (EC 2.3.1.12) (70 kDa mitochondrial autoantigen of primary biliary cirrhosis) (PBC) (Dihydrolipoamide acetyltransferase component of pyruvate dehydrogenase complex) (M2 antigen complex 70 kDa subunit) (Pyruvate dehydrogenase complex component E2) (PDC-E2) (PDCE2) |
| P19367 | HXK1_HUMAN | Hexokinase-1 (EC 2.7.1.1) (Brain form hexokinase) (Hexokinase type I) (HK I) |
| P07900 | HS90A_HUMAN | Heat shock protein HSP 90-alpha (Heat shock 86 kDa) (HSP 86) (HSP86) (Lipopolysaccharide-associated protein 2) (LAP-2) (LPS-associated protein 2) (Renal carcinoma antigen NY-REN-38) |
| P53007 | TXTP_HUMAN | Tricarboxylate transport protein, mitochondrial (Citrate transport protein) (CTP) (Solute carrier family 25 member 1) (Tricarboxylate carrier protein) |
| P57088 | TMM33_HUMAN | Transmembrane protein 33 (Protein DB83) (SHINC-3) |
| Q16555 | DPYL2_HUMAN | Dihydropyrimidinase-related protein 2 (DRP-2) (Collapsin response mediator protein 2) (CRMP-2) (N2A3) (Unc-33-like phosphoprotein 2) (ULIP-2) |
| O43148 | MCES_HUMAN | mRNA cap guanine-N7 methyltransferase (EC 2.1.1.56) (RG7MT1) (mRNA (guanine-N(7)-)-methyltransferase) (mRNA cap methyltransferase) (hCMT1) (hMet) (hcm1p) |
| P40939 | ECHA_HUMAN | Trifunctional enzyme subunit alpha, mitochondrial (78 kDa gastrin-binding protein) (TP-alpha) [Includes: Long-chain enoyl-CoA hydratase (EC 4.2.1.17); Long chain 3-hydroxyacyl-CoA dehydrogenase (EC 1.1.1.211)] |
| Q13131 | AAPK1_HUMAN | 5'-AMP-activated protein kinase catalytic subunit alpha-1 (AMPK subunit alpha-1) (EC 2.7.11.1) (Acetyl-CoA carboxylase kinase) (ACACA kinase) (EC 2.7.11.27) (Hydroxymethylglutaryl-CoA reductase kinase) (HMGCR kinase) (EC 2.7.11.31) (Tau-protein kinase PRKAA1) (EC 2.7.11.26) |
| Q9BSJ2 | GCP2_HUMAN | Gamma-tubulin complex component 2 (GCP-2) (hGCP2) (Gamma-ring complex protein 103 kDa) (h103p) (hGrip103) (Spindle pole body protein Spc97 homolog) (hSpc97) |
| P51531 | SMCA2_HUMAN | Probable global transcription activator SNF2L2 (EC 3.6.4.-) (ATP-dependent helicase SMARCA2) (BRG1-associated factor 190B) (BAF190B) (Protein brahma homolog) (hBRM) (SNF2-alpha) (SWI/SNF-related matrix-associated actin-dependent regulator of chromatin subfamily A member 2) |
| P46977 | STT3A_HUMAN | Dolichyl-diphosphooligosaccharide--protein glycosyltransferase subunit STT3A (Oligosaccharyl transferase subunit STT3A) (STT3-A) (EC 2.4.99.18) (B5) (Integral membrane protein 1) (Transmembrane protein TMC) |
| P22695 | QCR2_HUMAN | Cytochrome b-c1 complex subunit 2, mitochondrial (Complex III subunit 2) (Core protein II) (Ubiquinol-cytochrome-c reductase complex core protein 2) |
| P21283 | VATC1_HUMAN | V-type proton ATPase subunit C 1 (V-ATPase subunit C 1) (Vacuolar proton pump subunit C 1) |
| P00403 | COX2_HUMAN | Cytochrome c oxidase subunit 2 (Cytochrome c oxidase polypeptide II) |
| O75489 | NDUS3_HUMAN | NADH dehydrogenase [ubiquinone] iron-sulfur protein 3, mitochondrial (EC 1.6.5.3) (EC 1.6.99.3) (Complex I-30kD) (CI-30kD) (NADH-ubiquinone oxidoreductase 30 kDa subunit) |
| P47804 | RGR_HUMAN | RPE-retinal G protein-coupled receptor |
| Q9UDX5 | MTFP1_HUMAN | Mitochondrial fission process protein 1 (Mitochondrial 18 kDa protein) (MTP18) |
| P04181 | OAT_HUMAN | Ornithine aminotransferase, mitochondrial (EC 2.6.1.13) (Ornithine delta-aminotransferase) (Ornithine--oxo-acid aminotransferase) [Cleaved into: Ornithine aminotransferase, hepatic form; Ornithine aminotransferase, renal form] |
| P35613 | BASI_HUMAN | Basigin (5F7) (Collagenase stimulatory factor) (Extracellular matrix metalloproteinase inducer) (EMMPRIN) (Leukocyte activation antigen M6) (OK blood group antigen) (Tumor cell-derived collagenase stimulatory factor) (TCSF) (CD antigen CD147) |
| Q9Y3D6 | FIS1_HUMAN | Mitochondrial fission 1 protein (FIS1 homolog) (hFis1) (Tetratricopeptide repeat protein 11) (TPR repeat protein 11) |
| P50993 | AT1A2_HUMAN | Sodium/potassium-transporting ATPase subunit alpha-2 (Na(+)/K(+) ATPase alpha-2 subunit) (EC 3.6.3.9) (Sodium pump subunit alpha-2) |
| P63208 | SKP1_HUMAN | S-phase kinase-associated protein 1 (Cyclin-A/CDK2-associated protein p19) (p19A) (Organ of Corti protein 2) (OCP-2) (Organ of Corti protein II) (OCP-II) (RNA polymerase II elongation factor-like protein) (SIII) (Transcription elongation factor B polypeptide 1-like) (p19skp1) |
| Q00688 | FKBP3_HUMAN | Peptidyl-prolyl cis-trans isomerase FKBP3 (PPIase FKBP3) (EC 5.2.1.8) (25 kDa FK506-binding protein) (25 kDa FKBP) (FKBP-25) (FK506-binding protein 3) (FKBP-3) (Immunophilin FKBP25) (Rapamycin-selective 25 kDa immunophilin) (Rotamase) |
| Q9BRX8 | F213A_HUMAN | Redox-regulatory protein FAM213A (Peroxiredoxin-like 2 activated in M-CSF stimulated monocytes) (Protein PAMM) |
| P05023 | AT1A1_HUMAN | Sodium/potassium-transporting ATPase subunit alpha-1 (Na(+)/K(+) ATPase alpha-1 subunit) (EC 3.6.3.9) (Sodium pump subunit alpha-1) |
| P41250 | GARS_HUMAN | Glycine--tRNA ligase (EC 3.6.1.17) (EC 6.1.1.14) (Diadenosine tetraphosphate synthetase) (AP-4-A synthetase) (Glycyl-tRNA synthetase) (GlyRS) |
| Q9BUF5 | TBB6_HUMAN | Tubulin beta-6 chain (Tubulin beta class V) |
| P48449 | ERG7_HUMAN | Lanosterol synthase (EC 5.4.99.7) (2,3-epoxysqualene--lanosterol cyclase) (Oxidosqualene--lanosterol cyclase) (OSC) (hOSC) |
| Q8IWA5 | CTL2_HUMAN | Choline transporter-like protein 2 (Solute carrier family 44 member 2) |
| H7C072 | H7C072_HUMAN | THO complex subunit 5 homolog (Fragment) |
| P49418 | AMPH_HUMAN | Amphiphysin |
| P60228 | EIF3E_HUMAN | Eukaryotic translation initiation factor 3 subunit E (eIF3e) (Eukaryotic translation initiation factor 3 subunit 6) (Viral integration site protein INT-6 homolog) (eIF-3 p48) |
| P36578 | RL4_HUMAN | 60S ribosomal protein L4 (60S ribosomal protein L1) (Large ribosomal subunit protein uL4) |
| P31153 | METK2_HUMAN | S-adenosylmethionine synthase isoform type-2 (AdoMet synthase 2) (EC 2.5.1.6) (Methionine adenosyltransferase 2) (MAT 2) (Methionine adenosyltransferase II) (MAT-II) |
| Q13564 | ULA1_HUMAN | NEDD8-activating enzyme E1 regulatory subunit (Amyloid beta precursor protein-binding protein 1, 59 kDa) (APP-BP1) (Amyloid protein-binding protein 1) (Proto-oncogene protein 1) |
| P13645 | K1C10_HUMAN | Keratin, type I cytoskeletal 10 (Cytokeratin-10) (CK-10) (Keratin-10) (K10) |
| Q00765 | REEP5_HUMAN | Receptor expression-enhancing protein 5 (Polyposis locus protein 1) (Protein TB2) |
| Q8IZ83 | A16A1_HUMAN | Aldehyde dehydrogenase family 16 member A1 |
| Q9Y4I1 | MYO5A_HUMAN | Unconventional myosin-Va (Dilute myosin heavy chain, non-muscle) (Myosin heavy chain 12) (Myosin-12) (Myoxin) |
| P46782 | RS5_HUMAN | 40S ribosomal protein S5 (Small ribosomal subunit protein uS7) [Cleaved into: 40S ribosomal protein S5, N-terminally processed] |
| P21579 | SYT1_HUMAN | Synaptotagmin-1 (Synaptotagmin I) (SytI) (p65) |
| O95486 | SC24A_HUMAN | Protein transport protein Sec24A (SEC24-related protein A) |
| Q15046 | SYK_HUMAN | Lysine--tRNA ligase (EC 6.1.1.6) (Lysyl-tRNA synthetase) (LysRS) |
| O43432 | IF4G3_HUMAN | Eukaryotic translation initiation factor 4 gamma 3 (eIF-4-gamma 3) (eIF-4G 3) (eIF4G 3) (eIF-4-gamma II) (eIF4GII) |
| F8W9U4 | F8W9U4_HUMAN | Microtubule-associated protein |
| Q92538 | GBF1_HUMAN | Golgi-specific brefeldin A-resistance guanine nucleotide exchange factor 1 (BFA-resistant GEF 1) |
| P32969 | RL9_HUMAN | 60S ribosomal protein L9 (Large ribosomal subunit protein uL6) |
| P49840 | GSK3A_HUMAN | Glycogen synthase kinase-3 alpha (GSK-3 alpha) (EC 2.7.11.26) (Serine/threonine-protein kinase GSK3A) (EC 2.7.11.1) |
| O00519 | FAAH1_HUMAN | Fatty-acid amide hydrolase 1 (EC 3.5.1.99) (Anandamide amidohydrolase 1) (Oleamide hydrolase 1) |
| P08865 | RSSA_HUMAN | 40S ribosomal protein SA (37 kDa laminin receptor precursor) (37LRP) (37/67 kDa laminin receptor) (LRP/LR) (67 kDa laminin receptor) (67LR) (Colon carcinoma laminin-binding protein) (Laminin receptor 1) (LamR) (Laminin-binding protein precursor p40) (LBP/p40) (Multidrug resistance-associated protein MGr1-Ag) (NEM/1CHD4) (Small ribosomal subunit protein uS2) |
| Q8N684 | CPSF7_HUMAN | Cleavage and polyadenylation specificity factor subunit 7 (Cleavage and polyadenylation specificity factor 59 kDa subunit) (CFIm59) (CPSF 59 kDa subunit) (Pre-mRNA cleavage factor Im 59 kDa subunit) |
| Q8IXB1 | DJC10_HUMAN | DnaJ homolog subfamily C member 10 (EC 1.8.4.-) (Endoplasmic reticulum DNA J domain-containing protein 5) (ER-resident protein ERdj5) (ERdj5) (Macrothioredoxin) (MTHr) |
| F8W726 | F8W726_HUMAN | Ubiquitin-associated protein 2-like |
| Q12931 | TRAP1_HUMAN | Heat shock protein 75 kDa, mitochondrial (HSP 75) (TNFR-associated protein 1) (Tumor necrosis factor type 1 receptor-associated protein) (TRAP-1) |
| Q8TAQ2 | SMRC2_HUMAN | SWI/SNF complex subunit SMARCC2 (BRG1-associated factor 170) (BAF170) (SWI/SNF complex 170 kDa subunit) (SWI/SNF-related matrix-associated actin-dependent regulator of chromatin subfamily C member 2) |
| P40925 | MDHC_HUMAN | Malate dehydrogenase, cytoplasmic (EC 1.1.1.37) (Cytosolic malate dehydrogenase) (Diiodophenylpyruvate reductase) (EC 1.1.1.96) |
| P35580 | MYH10_HUMAN | Myosin-10 (Cellular myosin heavy chain, type B) (Myosin heavy chain 10) (Myosin heavy chain, non-muscle IIb) (Non-muscle myosin heavy chain B) (NMMHC-B) (Non-muscle myosin heavy chain IIb) (NMMHC II-b) (NMMHC-IIB) |
| Q96JE9 | MAP6_HUMAN | Microtubule-associated protein 6 (MAP-6) (Stable tubule-only polypeptide) (STOP) |
| P11233 | RALA_HUMAN | Ras-related protein Ral-A |
| Q15393 | SF3B3_HUMAN | Splicing factor 3B subunit 3 (Pre-mRNA-splicing factor SF3b 130 kDa subunit) (SF3b130) (STAF130) (Spliceosome-associated protein 130) (SAP 130) |
| P33121 | ACSL1_HUMAN | Long-chain-fatty-acid--CoA ligase 1 (EC 6.2.1.3) (Acyl-CoA synthetase 1) (ACS1) (Long-chain acyl-CoA synthetase 1) (LACS 1) (Long-chain acyl-CoA synthetase 2) (LACS 2) (Long-chain fatty acid-CoA ligase 2) (Palmitoyl-CoA ligase 1) (Palmitoyl-CoA ligase 2) |
| Q9P2U7 | VGLU1_HUMAN | Vesicular glutamate transporter 1 (VGluT1) (Brain-specific Na(+)-dependent inorganic phosphate cotransporter) (Solute carrier family 17 member 7) |
| Q9UKS6 | PACN3_HUMAN | Protein kinase C and casein kinase substrate in neurons protein 3 (SH3 domain-containing protein 6511) |
| P30531 | SC6A1_HUMAN | Sodium- and chloride-dependent GABA transporter 1 (GAT-1) (Solute carrier family 6 member 1) |
| Q9Y570 | PPME1_HUMAN | Protein phosphatase methylesterase 1 (PME-1) (EC 3.1.1.89) |
| P61803 | DAD1_HUMAN | Dolichyl-diphosphooligosaccharide--protein glycosyltransferase subunit DAD1 (Oligosaccharyl transferase subunit DAD1) (Defender against cell death 1) (DAD-1) |
| P84074 | HPCA_HUMAN | Neuron-specific calcium-binding protein hippocalcin (Calcium-binding protein BDR-2) |
| P11047 | LAMC1_HUMAN | Laminin subunit gamma-1 (Laminin B2 chain) (Laminin-1 subunit gamma) (Laminin-10 subunit gamma) (Laminin-11 subunit gamma) (Laminin-2 subunit gamma) (Laminin-3 subunit gamma) (Laminin-4 subunit gamma) (Laminin-6 subunit gamma) (Laminin-7 subunit gamma) (Laminin-8 subunit gamma) (Laminin-9 subunit gamma) (S-laminin subunit gamma) (S-LAM gamma) |
| O15371 | EIF3D_HUMAN | Eukaryotic translation initiation factor 3 subunit D (eIF3d) (Eukaryotic translation initiation factor 3 subunit 7) (eIF-3-zeta) (eIF3 p66) |
| O75122 | CLAP2_HUMAN | CLIP-associating protein 2 (Cytoplasmic linker-associated protein 2) (Protein Orbit homolog 2) (hOrbit2) |
| Q969V3 | NCLN_HUMAN | Nicalin (Nicastrin-like protein) |
| O14561 | ACPM_HUMAN | Acyl carrier protein, mitochondrial (ACP) (CI-SDAP) (NADH-ubiquinone oxidoreductase 9.6 kDa subunit) |
| O00154 | BACH_HUMAN | Cytosolic acyl coenzyme A thioester hydrolase (EC 3.1.2.2) (Acyl-CoA thioesterase 7) (Brain acyl-CoA hydrolase) (BACH) (CTE-IIa) (CTE-II) (Long chain acyl-CoA thioester hydrolase) |
| O15382 | BCAT2_HUMAN | Branched-chain-amino-acid aminotransferase, mitochondrial (BCAT(m)) (EC 2.6.1.42) (Placental protein 18) (PP18) |
| P40429 | RL13A_HUMAN | 60S ribosomal protein L13a (23 kDa highly basic protein) (Large ribosomal subunit protein uL13) |
| Q6PI48 | SYDM_HUMAN | Aspartate--tRNA ligase, mitochondrial (EC 6.1.1.12) (Aspartyl-tRNA synthetase) (AspRS) |
| Q7L099 | RUFY3_HUMAN | Protein RUFY3 (RUN and FYVE domain-containing protein 3) (Rap2-interacting protein x) (RIPx) (Single axon-regulated protein) (Singar) |
| P63167 | DYL1_HUMAN | Dynein light chain 1, cytoplasmic (8 kDa dynein light chain) (DLC8) (Dynein light chain LC8-type 1) (Protein inhibitor of neuronal nitric oxide synthase) (PIN) |
| P48147 | PPCE_HUMAN | Prolyl endopeptidase (PE) (EC 3.4.21.26) (Post-proline cleaving enzyme) |
| Q13423 | NNTM_HUMAN | NAD(P) transhydrogenase, mitochondrial (EC 1.6.1.2) (Nicotinamide nucleotide transhydrogenase) (Pyridine nucleotide transhydrogenase) |
| Q96FZ7 | CHMP6_HUMAN | Charged multivesicular body protein 6 (Chromatin-modifying protein 6) (Vacuolar protein sorting-associated protein 20) (Vps20) (hVps20) |
| P30519 | HMOX2_HUMAN | Heme oxygenase 2 (HO-2) (EC 1.14.14.18) |
| Q92696 | PGTA_HUMAN | Geranylgeranyl transferase type-2 subunit alpha (EC 2.5.1.60) (Geranylgeranyl transferase type II subunit alpha) (Rab geranyl-geranyltransferase subunit alpha) (Rab GG transferase alpha) (Rab GGTase alpha) (Rab geranylgeranyltransferase subunit alpha) |
| Q3KQU3 | MA7D1_HUMAN | MAP7 domain-containing protein 1 (Arginine/proline-rich coiled-coil domain-containing protein 1) (Proline/arginine-rich coiled-coil domain-containing protein 1) |
| P39060 | COIA1_HUMAN | Collagen alpha-1(XVIII) chain [Cleaved into: Endostatin] |
| P13591 | NCAM1_HUMAN | Neural cell adhesion molecule 1 (N-CAM-1) (NCAM-1) (CD antigen CD56) |
| Q8NBS9 | TXND5_HUMAN | Thioredoxin domain-containing protein 5 (Endoplasmic reticulum resident protein 46) (ER protein 46) (ERp46) (Thioredoxin-like protein p46) |
| Q9BRG1 | VPS25_HUMAN | Vacuolar protein-sorting-associated protein 25 (hVps25) (Dermal papilla-derived protein 9) (ELL-associated protein of 20 kDa) (ESCRT-II complex subunit VPS25) |
| Q9Y4E6 | WDR7_HUMAN | WD repeat-containing protein 7 (Rabconnectin-3 beta) (TGF-beta resistance-associated protein TRAG) |
| O60282 | KIF5C_HUMAN | Kinesin heavy chain isoform 5C (Kinesin heavy chain neuron-specific 2) |
| J3KNA1 | J3KNA1_HUMAN | Kinesin-like protein |
| Q9P0L0 | VAPA_HUMAN | Vesicle-associated membrane protein-associated protein A (VAMP-A) (VAMP-associated protein A) (VAP-A) (33 kDa VAMP-associated protein) (VAP-33) |
| O95487 | SC24B_HUMAN | Protein transport protein Sec24B (SEC24-related protein B) |
| P14868 | SYDC_HUMAN | Aspartate--tRNA ligase, cytoplasmic (EC 6.1.1.12) (Aspartyl-tRNA synthetase) (AspRS) (Cell proliferation-inducing gene 40 protein) |
| P26639 | SYTC_HUMAN | Threonine--tRNA ligase, cytoplasmic (EC 6.1.1.3) (Threonyl-tRNA synthetase) (ThrRS) |
| P51160 | PDE6C_HUMAN | Cone cGMP-specific 3',5'-cyclic phosphodiesterase subunit alpha' (EC 3.1.4.35) (cGMP phosphodiesterase 6C) |
| Q8N573 | OXR1_HUMAN | Oxidation resistance protein 1 |
| P62854 | RS26_HUMAN | 40S ribosomal protein S26 (Small ribosomal subunit protein eS26) |
| O75533 | SF3B1_HUMAN | Splicing factor 3B subunit 1 (Pre-mRNA-splicing factor SF3b 155 kDa subunit) (SF3b155) (Spliceosome-associated protein 155) (SAP 155) |
| P04275 | VWF_HUMAN | von Willebrand factor (vWF) [Cleaved into: von Willebrand antigen 2 (von Willebrand antigen II)] |
| Q4J6C6 | PPCEL_HUMAN | Prolyl endopeptidase-like (EC 3.4.21.-) (Prolylendopeptidase-like) |
| P20020 | AT2B1_HUMAN | Plasma membrane calcium-transporting ATPase 1 (PMCA1) (EC 3.6.3.8) (Plasma membrane calcium ATPase isoform 1) (Plasma membrane calcium pump isoform 1) |
| Q01814 | AT2B2_HUMAN | Plasma membrane calcium-transporting ATPase 2 (PMCA2) (EC 3.6.3.8) (Plasma membrane calcium ATPase isoform 2) (Plasma membrane calcium pump isoform 2) |
| O94906 | PRP6_HUMAN | Pre-mRNA-processing factor 6 (Androgen receptor N-terminal domain-transactivating protein 1) (ANT-1) (PRP6 homolog) (U5 snRNP-associated 102 kDa protein) (U5-102 kDa protein) |
| Q9BSJ8 | ESYT1_HUMAN | Extended synaptotagmin-1 (E-Syt1) (Membrane-bound C2 domain-containing protein) |
| O60264 | SMCA5_HUMAN | SWI/SNF-related matrix-associated actin-dependent regulator of chromatin subfamily A member 5 (SWI/SNF-related matrix-associated actin-dependent regulator of chromatin A5) (EC 3.6.4.-) (Sucrose nonfermenting protein 2 homolog) (hSNF2H) |
| O60488 | ACSL4_HUMAN | Long-chain-fatty-acid--CoA ligase 4 (EC 6.2.1.3) (Long-chain acyl-CoA synthetase 4) (LACS 4) |
| Q9Y639 | NPTN_HUMAN | Neuroplastin (Stromal cell-derived receptor 1) (SDR-1) |
| Q6P1M3 | L2GL2_HUMAN | Lethal(2) giant larvae protein homolog 2 (HGL) |
| H0Y921 | H0Y921_HUMAN | Phosphoglucomutase-2 (Fragment) |
| Q9UBB4 | ATX10_HUMAN | Ataxin-10 (Brain protein E46 homolog) (Spinocerebellar ataxia type 10 protein) |
| Q9NT62 | ATG3_HUMAN | Ubiquitin-like-conjugating enzyme ATG3 (EC 6.3.2.-) (Autophagy-related protein 3) (APG3-like) (hApg3) (Protein PC3-96) |
| O43301 | HS12A_HUMAN | Heat shock 70 kDa protein 12A |
| Q13813 | SPTN1_HUMAN | Spectrin alpha chain, non-erythrocytic 1 (Alpha-II spectrin) (Fodrin alpha chain) (Spectrin, non-erythroid alpha subunit) |
| Q06278 | AOXA_HUMAN | Aldehyde oxidase (EC 1.2.3.1) (Aldehyde oxidase 1) (Azaheterocycle hydroxylase) (EC 1.17.3.-) |
| Q8N0U8 | VKORL_HUMAN | Vitamin K epoxide reductase complex subunit 1-like protein 1 (VKORC1-like protein 1) (EC 1.17.4.4) |
| Q9UEY8 | ADDG_HUMAN | Gamma-adducin (Adducin-like protein 70) |
| Q8TBQ9 | KISHA_HUMAN | Protein kish-A (Transmembrane protein 167) (Transmembrane protein 167A) |
| Q96KG9 | SCYL1_HUMAN | N-terminal kinase-like protein (Coated vesicle-associated kinase of 90 kDa) (SCY1-like protein 1) (Telomerase regulation-associated protein) (Telomerase transcriptional element-interacting factor) (Teratoma-associated tyrosine kinase) |
| P55786 | PSA_HUMAN | Puromycin-sensitive aminopeptidase (PSA) (EC 3.4.11.14) (Cytosol alanyl aminopeptidase) (AAP-S) |
| P30740 | ILEU_HUMAN | Leukocyte elastase inhibitor (LEI) (Monocyte/neutrophil elastase inhibitor) (EI) (M/NEI) (Peptidase inhibitor 2) (PI-2) (Serpin B1) |
| P11277 | SPTB1_HUMAN | Spectrin beta chain, erythrocytic (Beta-I spectrin) |
| Q9H254 | SPTN4_HUMAN | Spectrin beta chain, non-erythrocytic 4 (Beta-IV spectrin) (Spectrin, non-erythroid beta chain 3) |
| P40616 | ARL1_HUMAN | ADP-ribosylation factor-like protein 1 |
| O60333 | KIF1B_HUMAN | Kinesin-like protein KIF1B (Klp) |
| Q5VV89 | Q5VV89_HUMAN | Microsomal glutathione S-transferase 3 |
| Q14204 | DYHC1_HUMAN | Cytoplasmic dynein 1 heavy chain 1 (Cytoplasmic dynein heavy chain 1) (Dynein heavy chain, cytosolic) |
| Q96SI9 | STRBP_HUMAN | Spermatid perinuclear RNA-binding protein |
| P53396 | ACLY_HUMAN | ATP-citrate synthase (EC 2.3.3.8) (ATP-citrate (pro-S-)-lyase) (ACL) (Citrate cleavage enzyme) |
| Q9Y295 | DRG1_HUMAN | Developmentally-regulated GTP-binding protein 1 (DRG-1) (Neural precursor cell expressed developmentally down-regulated protein 3) (NEDD-3) |
| P28288 | ABCD3_HUMAN | ATP-binding cassette sub-family D member 3 (70 kDa peroxisomal membrane protein) (PMP70) |
| P43007 | SATT_HUMAN | Neutral amino acid transporter A (Alanine/serine/cysteine/threonine transporter 1) (ASCT-1) (SATT) (Solute carrier family 1 member 4) |
| Q969U7 | PSMG2_HUMAN | Proteasome assembly chaperone 2 (PAC-2) (Hepatocellular carcinoma-susceptibility protein 3) (Tumor necrosis factor superfamily member 5-induced protein 1) |
| Q16720 | AT2B3_HUMAN | Plasma membrane calcium-transporting ATPase 3 (PMCA3) (EC 3.6.3.8) (Plasma membrane calcium ATPase isoform 3) (Plasma membrane calcium pump isoform 3) |
| Q8TCJ2 | STT3B_HUMAN | Dolichyl-diphosphooligosaccharide--protein glycosyltransferase subunit STT3B (Oligosaccharyl transferase subunit STT3B) (STT3-B) (EC 2.4.99.18) (Source of immunodominant MHC-associated peptides homolog) |
| Q15257 | PTPA_HUMAN | Serine/threonine-protein phosphatase 2A activator (EC 5.2.1.8) (PP2A, subunit B', PR53 isoform) (Phosphotyrosyl phosphatase activator) (PTPA) (Serine/threonine-protein phosphatase 2A regulatory subunit 4) (Serine/threonine-protein phosphatase 2A regulatory subunit B') |
| P18124 | RL7_HUMAN | 60S ribosomal protein L7 (Large ribosomal subunit protein uL30) |
| Q9Y6M7 | S4A7_HUMAN | Sodium bicarbonate cotransporter 3 (Electroneutral Na/HCO(3) cotransporter) (Sodium bicarbonate cotransporter 2) (Sodium bicarbonate cotransporter 2b) (Bicarbonate transporter) (Solute carrier family 4 member 7) |
| Q9UQM7 | KCC2A_HUMAN | Calcium/calmodulin-dependent protein kinase type II subunit alpha (CaM kinase II subunit alpha) (CaMK-II subunit alpha) (EC 2.7.11.17) |
| P46777 | RL5_HUMAN | 60S ribosomal protein L5 (Large ribosomal subunit protein uL18) |
| B7Z4B8 | B7Z4B8_HUMAN | Heterogeneous nuclear ribonucleoprotein U-like protein 1 (cDNA FLJ56481, highly similar to Heterogeneous nuclear ribonucleoprotein U-like protein 1) |
| P36915 | GNL1_HUMAN | Guanine nucleotide-binding protein-like 1 (GTP-binding protein HSR1) |
| Q9H0B6 | KLC2_HUMAN | Kinesin light chain 2 (KLC 2) |
| Q6IBS0 | TWF2_HUMAN | Twinfilin-2 (A6-related protein) (hA6RP) (Protein tyrosine kinase 9-like) (Twinfilin-1-like protein) |
| P37268 | FDFT_HUMAN | Squalene synthase (SQS) (SS) (EC 2.5.1.21) (FPP:FPP farnesyltransferase) (Farnesyl-diphosphate farnesyltransferase) |
| P49721 | PSB2_HUMAN | Proteasome subunit beta type-2 (EC 3.4.25.1) (Macropain subunit C7-I) (Multicatalytic endopeptidase complex subunit C7-I) (Proteasome component C7-I) |
| P62913 | RL11_HUMAN | 60S ribosomal protein L11 (CLL-associated antigen KW-12) (Large ribosomal subunit protein uL5) |
| Q00577 | PURA_HUMAN | Transcriptional activator protein Pur-alpha (Purine-rich single-stranded DNA-binding protein alpha) |
| P78417 | GSTO1_HUMAN | Glutathione S-transferase omega-1 (GSTO-1) (EC 2.5.1.18) (Glutathione S-transferase omega 1-1) (GSTO 1-1) (Glutathione-dependent dehydroascorbate reductase) (EC 1.8.5.1) (Monomethylarsonic acid reductase) (MMA(V) reductase) (EC 1.20.4.2) (S-(Phenacyl)glutathione reductase) (SPG-R) |
| Q9UKU0 | ACSL6_HUMAN | Long-chain-fatty-acid--CoA ligase 6 (EC 6.2.1.3) (Long-chain acyl-CoA synthetase 6) (LACS 6) |
| P37235 | HPCL1_HUMAN | Hippocalcin-like protein 1 (Calcium-binding protein BDR-1) (HLP2) (Visinin-like protein 3) (VILIP-3) |
| Q12907 | LMAN2_HUMAN | Vesicular integral-membrane protein VIP36 (Glycoprotein GP36b) (Lectin mannose-binding 2) (Vesicular integral-membrane protein 36) (VIP36) |
| Q562R1 | ACTBL_HUMAN | Beta-actin-like protein 2 (Kappa-actin) |
| Q8N335 | GPD1L_HUMAN | Glycerol-3-phosphate dehydrogenase 1-like protein (GPD1-L) (EC 1.1.1.8) |
| Q14240 | IF4A2_HUMAN | Eukaryotic initiation factor 4A-II (eIF-4A-II) (eIF4A-II) (EC 3.6.4.13) (ATP-dependent RNA helicase eIF4A-2) |
| Q02978 | M2OM_HUMAN | Mitochondrial 2-oxoglutarate/malate carrier protein (OGCP) (Solute carrier family 25 member 11) |
| P62070 | RRAS2_HUMAN | Ras-related protein R-Ras2 (Ras-like protein TC21) (Teratocarcinoma oncogene) |
| Q02218 | ODO1_HUMAN | 2-oxoglutarate dehydrogenase, mitochondrial (EC 1.2.4.2) (2-oxoglutarate dehydrogenase complex component E1) (OGDC-E1) (Alpha-ketoglutarate dehydrogenase) |
| A0A024R4E5 | A0A024R4E5_HUMAN | High density lipoprotein binding protein (Vigilin), isoform CRA_a (Vigilin) |
| O75306 | NDUS2_HUMAN | NADH dehydrogenase [ubiquinone] iron-sulfur protein 2, mitochondrial (EC 1.6.5.3) (EC 1.6.99.3) (Complex I-49kD) (CI-49kD) (NADH-ubiquinone oxidoreductase 49 kDa subunit) |
| P52429 | DGKE_HUMAN | Diacylglycerol kinase epsilon (DAG kinase epsilon) (EC 2.7.1.107) (Diglyceride kinase epsilon) (DGK-epsilon) |
| Q9UQ03 | COR2B_HUMAN | Coronin-2B (Coronin-like protein C) (Clipin-C) (Protein FC96) |
| P35527 | K1C9_HUMAN | Keratin, type I cytoskeletal 9 (Cytokeratin-9) (CK-9) (Keratin-9) (K9) |
| O15144 | ARPC2_HUMAN | Actin-related protein 2/3 complex subunit 2 (Arp2/3 complex 34 kDa subunit) (p34-ARC) |
| Q99459 | CDC5L_HUMAN | Cell division cycle 5-like protein (Cdc5-like protein) (Pombe cdc5-related protein) |
| P46926 | GNPI1_HUMAN | Glucosamine-6-phosphate isomerase 1 (EC 3.5.99.6) (Glucosamine-6-phosphate deaminase 1) (GNPDA 1) (GlcN6P deaminase 1) (Oscillin) |
| Q9H4G0 | E41L1_HUMAN | Band 4.1-like protein 1 (Neuronal protein 4.1) (4.1N) |
| Q96QR8 | PURB_HUMAN | Transcriptional activator protein Pur-beta (Purine-rich element-binding protein B) |
| P51148 | RAB5C_HUMAN | Ras-related protein Rab-5C (L1880) (RAB5L) |
| Q9HB71 | CYBP_HUMAN | Calcyclin-binding protein (CacyBP) (hCacyBP) (S100A6-binding protein) (Siah-interacting protein) |
| Q07954 | LRP1_HUMAN | Prolow-density lipoprotein receptor-related protein 1 (LRP-1) (Alpha-2-macroglobulin receptor) (A2MR) (Apolipoprotein E receptor) (APOER) (CD antigen CD91) [Cleaved into: Low-density lipoprotein receptor-related protein 1 85 kDa subunit (LRP-85); Low-density lipoprotein receptor-related protein 1 515 kDa subunit (LRP-515); Low-density lipoprotein receptor-related protein 1 intracellular domain (LRPICD)] |
| Q8TDJ6 | DMXL2_HUMAN | DmX-like protein 2 (Rabconnectin-3) |
| Q6IAA8 | LTOR1_HUMAN | Ragulator complex protein LAMTOR1 (Late endosomal/lysosomal adaptor and MAPK and MTOR activator 1) (Lipid raft adaptor protein p18) (Protein associated with DRMs and endosomes) (p27Kip1-releasing factor from RhoA) (p27RF-Rho) |
| P62745 | RHOB_HUMAN | Rho-related GTP-binding protein RhoB (Rho cDNA clone 6) (h6) |
| P62888 | RL30_HUMAN | 60S ribosomal protein L30 (Large ribosomal subunit protein eL30) |
| P62280 | RS11_HUMAN | 40S ribosomal protein S11 (Small ribosomal subunit protein uS17) |
| P63244 | RACK1_HUMAN | Receptor of activated protein C kinase 1 (Cell proliferation-inducing gene 21 protein) (Guanine nucleotide-binding protein subunit beta-2-like 1) (Guanine nucleotide-binding protein subunit beta-like protein 12.3) (Human lung cancer oncogene 7 protein) (HLC-7) (Receptor for activated C kinase) (Small ribosomal subunit protein RACK1) [Cleaved into: Receptor of activated protein C kinase 1, N-terminally processed (Guanine nucleotide-binding protein subunit beta-2-like 1, N-terminally processed)] |
| Q96AG4 | LRC59_HUMAN | Leucine-rich repeat-containing protein 59 (Ribosome-binding protein p34) (p34) |
| P46783 | RS10_HUMAN | 40S ribosomal protein S10 (Small ribosomal subunit protein eS10) |
| Q07021 | C1QBP_HUMAN | Complement component 1 Q subcomponent-binding protein, mitochondrial (ASF/SF2-associated protein p32) (Glycoprotein gC1qBP) (C1qBP) (Hyaluronan-binding protein 1) (Mitochondrial matrix protein p32) (gC1q-R protein) (p33) |
| P30825 | CTR1_HUMAN | High affinity cationic amino acid transporter 1 (CAT-1) (CAT1) (Ecotropic retroviral leukemia receptor homolog) (Ecotropic retrovirus receptor homolog) (Solute carrier family 7 member 1) (System Y+ basic amino acid transporter) |
| Q9H4B7 | TBB1_HUMAN | Tubulin beta-1 chain |
| O43395 | PRPF3_HUMAN | U4/U6 small nuclear ribonucleoprotein Prp3 (Pre-mRNA-splicing factor 3) (hPrp3) (U4/U6 snRNP 90 kDa protein) |
| Q9ULC5 | ACSL5_HUMAN | Long-chain-fatty-acid--CoA ligase 5 (EC 6.2.1.3) (Long-chain acyl-CoA synthetase 5) (LACS 5) |
| A0A0A0MRE6 | A0A0A0MRE6_HUMAN | WD repeat-containing protein 47 |
| Q8TBC4 | UBA3_HUMAN | NEDD8-activating enzyme E1 catalytic subunit (EC 6.3.2.-) (NEDD8-activating enzyme E1C) (Ubiquitin-activating enzyme E1C) (Ubiquitin-like modifier-activating enzyme 3) (Ubiquitin-activating enzyme 3) |
| Q5T9Q5 | Q5T9Q5_HUMAN | Succinate--CoA ligase [ADP-forming] subunit beta, mitochondrial (Fragment) |
| D3DQV9 | D3DQV9_HUMAN | Eukaryotic translation initiation factor 4 gamma 2 (Eukaryotic translation initiation factor 4 gamma, 2, isoform CRA_b) (Fragment) |
| Q7Z6Z7 | HUWE1_HUMAN | E3 ubiquitin-protein ligase HUWE1 (EC 2.3.2.26) (ARF-binding protein 1) (ARF-BP1) (HECT, UBA and WWE domain-containing protein 1) (HECT-type E3 ubiquitin transferase HUWE1) (Homologous to E6AP carboxyl terminus homologous protein 9) (HectH9) (Large structure of UREB1) (LASU1) (Mcl-1 ubiquitin ligase E3) (Mule) (Upstream regulatory element-binding protein 1) (URE-B1) (URE-binding protein 1) |
| P43304 | GPDM_HUMAN | Glycerol-3-phosphate dehydrogenase, mitochondrial (GPD-M) (GPDH-M) (EC 1.1.5.3) (mtGPD) |
| P04216 | THY1_HUMAN | Thy-1 membrane glycoprotein (CDw90) (Thy-1 antigen) (CD antigen CD90) |
| Q9BVA1 | TBB2B_HUMAN | Tubulin beta-2B chain |
| Q13885 | TBB2A_HUMAN | Tubulin beta-2A chain (Tubulin beta class IIa) |
| P68371 | TBB4B_HUMAN | Tubulin beta-4B chain (Tubulin beta-2 chain) (Tubulin beta-2C chain) |
| P04350 | TBB4A_HUMAN | Tubulin beta-4A chain (Tubulin 5 beta) (Tubulin beta-4 chain) |
| P07437 | TBB5_HUMAN | Tubulin beta chain (Tubulin beta-5 chain) |
| Q13509 | TBB3_HUMAN | Tubulin beta-3 chain (Tubulin beta-4 chain) (Tubulin beta-III) |
| Q16186 | ADRM1_HUMAN | Proteasomal ubiquitin receptor ADRM1 (110 kDa cell membrane glycoprotein) (Gp110) (Adhesion-regulating molecule 1) (ARM-1) (Proteasome regulatory particle non-ATPase 13) (hRpn13) (Rpn13 homolog) |
| Q9UJZ1 | STML2_HUMAN | Stomatin-like protein 2, mitochondrial (SLP-2) (EPB72-like protein 2) (Paraprotein target 7) (Paratarg-7) |
| O14735 | CDIPT_HUMAN | CDP-diacylglycerol--inositol 3-phosphatidyltransferase (EC 2.7.8.11) (Phosphatidylinositol synthase) (PI synthase) (PtdIns synthase) |
| Q9NUJ1 | ABHDA_HUMAN | Mycophenolic acid acyl-glucuronide esterase, mitochondrial (EC 3.1.1.93) (Alpha/beta hydrolase domain-containing protein 10) (Abhydrolase domain-containing protein 10) |
| O14610 | GBGT2_HUMAN | Guanine nucleotide-binding protein G(I)/G(S)/G(O) subunit gamma-T2 (G gamma-C) (G-gamma-8) (G-gamma-9) (Guanine nucleotide binding protein gamma transducing activity polypeptide 2) |
| P62244 | RS15A_HUMAN | 40S ribosomal protein S15a (Small ribosomal subunit protein uS8) |
| Q07960 | RHG01_HUMAN | Rho GTPase-activating protein 1 (CDC42 GTPase-activating protein) (GTPase-activating protein rhoGAP) (Rho-related small GTPase protein activator) (Rho-type GTPase-activating protein 1) (p50-RhoGAP) |
| P25705 | ATPA_HUMAN | ATP synthase subunit alpha, mitochondrial |
| Q9NS69 | TOM22_HUMAN | Mitochondrial import receptor subunit TOM22 homolog (hTom22) (1C9-2) (Translocase of outer membrane 22 kDa subunit homolog) |
| P62701 | RS4X_HUMAN | 40S ribosomal protein S4, X isoform (SCR10) (Single copy abundant mRNA protein) (Small ribosomal subunit protein eS4) |
| P43490 | NAMPT_HUMAN | Nicotinamide phosphoribosyltransferase (NAmPRTase) (Nampt) (EC 2.4.2.12) (Pre-B-cell colony-enhancing factor 1) (Pre-B cell-enhancing factor) (Visfatin) |
| O76070 | SYUG_HUMAN | Gamma-synuclein (Breast cancer-specific gene 1 protein) (Persyn) (Synoretin) (SR) |
| P39023 | RL3_HUMAN | 60S ribosomal protein L3 (HIV-1 TAR RNA-binding protein B) (TARBP-B) (Large ribosomal subunit protein uL3) |
| H7BXY3 | H7BXY3_HUMAN | Putative ATP-dependent RNA helicase DHX30 |
| A0A024R216 | A0A024R216_HUMAN | Hepatoma-derived growth factor, related protein 3, isoform CRA_a (Hepatoma-derived growth factor-related protein 3) |
| P22460 | KCNA5_HUMAN | Potassium voltage-gated channel subfamily A member 5 (HPCN1) (Voltage-gated potassium channel HK2) (Voltage-gated potassium channel subunit Kv1.5) |
| O43615 | TIM44_HUMAN | Mitochondrial import inner membrane translocase subunit TIM44 |
| Q9BXK5 | B2L13_HUMAN | Bcl-2-like protein 13 (Bcl2-L-13) (Bcl-rambo) (Protein Mil1) |
| Q8IYB4 | PEX5R_HUMAN | PEX5-related protein (PEX2-related protein) (PEX5-like protein) (Peroxin-5-related protein) (Peroxisome biogenesis factor 5-like) (Tetratricopeptide repeat-containing Rab8b-interacting protein) (Pex5Rp) (TRIP8b) |
| O60506 | HNRPQ_HUMAN | Heterogeneous nuclear ribonucleoprotein Q (hnRNP Q) (Glycine- and tyrosine-rich RNA-binding protein) (GRY-RBP) (NS1-associated protein 1) (Synaptotagmin-binding, cytoplasmic RNA-interacting protein) |
| Q8IYT4 | KATL2_HUMAN | Katanin p60 ATPase-containing subunit A-like 2 (Katanin p60 subunit A-like 2) (EC 3.6.4.3) (p60 katanin-like 2) |
| P53814 | SMTN_HUMAN | Smoothelin |
| Q3BDU5 | Q3BDU5_HUMAN | Prelamin-A/C (Rhabdomyosarcoma antigen MU-RMS-40.12) |
