# Supplementary material for: Proteomic analysis of the human retina reveals region-specific susceptibilities to metabolic- and oxidative stress-related diseases
Source: PLoS One. 2018 Feb 21;13(2):e0193250. doi: 10.1371/journal.pone.0193250 (PMC5821407; doi:10.1371/journal.pone.0193250)
Supplement: S4 Table — (DOCX) [file pone.0193250.s015.docx]

**Supplemental Table 4. Differentially-expressed proteins in the juxta-macular retina.**

| **UniProt ID** | **Entry name** | **Protein names** |
| --- | --- | --- |
| A0A024R216 | A0A024R216_HUMAN | Hepatoma-derived growth factor, related protein 3, isoform CRA_a (Hepatoma-derived growth factor-related protein 3) |
| O94973 | AP2A2_HUMAN | AP-2 complex subunit alpha-2 (100 kDa coated vesicle protein C) (Adaptor protein complex AP-2 subunit alpha-2) (Adaptor-related protein complex 2 subunit alpha-2) (Alpha-adaptin C) (Alpha2-adaptin) (Clathrin assembly protein complex 2 alpha-C large chain) (Huntingtin yeast partner J) (Huntingtin-interacting protein 9) (HIP-9) (Huntingtin-interacting protein J) (Plasma membrane adaptor HA2/AP2 adaptin alpha C subunit) |
| Q9HCD5 | NCOA5_HUMAN | Nuclear receptor coactivator 5 (NCoA-5) (Coactivator independent of AF-2) (CIA) |
| O76070 | SYUG_HUMAN | Gamma-synuclein (Breast cancer-specific gene 1 protein) (Persyn) (Synoretin) (SR) |
| Q9P2J5 | SYLC_HUMAN | Leucine--tRNA ligase, cytoplasmic (EC 6.1.1.4) (Leucyl-tRNA synthetase) (LeuRS) |
| Q13509 | TBB3_HUMAN | Tubulin beta-3 chain (Tubulin beta-4 chain) (Tubulin beta-III) |
| P04350 | TBB4A_HUMAN | Tubulin beta-4A chain (Tubulin 5 beta) (Tubulin beta-4 chain) |
| P68371 | TBB4B_HUMAN | Tubulin beta-4B chain (Tubulin beta-2 chain) (Tubulin beta-2C chain) |
| Q9BVA1 | TBB2B_HUMAN | Tubulin beta-2B chain |
| Q13885 | TBB2A_HUMAN | Tubulin beta-2A chain (Tubulin beta class IIa) |
| B7Z4B8 | B7Z4B8_HUMAN | Heterogeneous nuclear ribonucleoprotein U-like protein 1 (cDNA FLJ56481, highly similar to Heterogeneous nuclear ribonucleoprotein U-like protein 1) |
| Q16555 | DPYL2_HUMAN | Dihydropyrimidinase-related protein 2 (DRP-2) (Collapsin response mediator protein 2) (CRMP-2) (N2A3) (Unc-33-like phosphoprotein 2) (ULIP-2) |
| P60842 | IF4A1_HUMAN | Eukaryotic initiation factor 4A-I (eIF-4A-I) (eIF4A-I) (EC 3.6.4.13) (ATP-dependent RNA helicase eIF4A-1) |
| Q9H0D6 | XRN2_HUMAN | 5'-3' exoribonuclease 2 (EC 3.1.13.-) (DHM1-like protein) (DHP protein) |
| Q8IVF2 | AHNK2_HUMAN | Protein AHNAK2 |
| O95140 | MFN2_HUMAN | Mitofusin-2 (EC 3.6.5.-) (Transmembrane GTPase MFN2) |
| P29992 | GNA11_HUMAN | Guanine nucleotide-binding protein subunit alpha-11 (G alpha-11) (G-protein subunit alpha-11) (Guanine nucleotide-binding protein G(y) subunit alpha) |
| Q9BY11 | PACN1_HUMAN | Protein kinase C and casein kinase substrate in neurons protein 1 (Syndapin-1) |
| Q86Y39 | NDUAB_HUMAN | NADH dehydrogenase [ubiquinone] 1 alpha subcomplex subunit 11 (Complex I-B14.7) (CI-B14.7) (NADH-ubiquinone oxidoreductase subunit B14.7) |
| P62847 | RS24_HUMAN | 40S ribosomal protein S24 (Small ribosomal subunit protein eS24) |
| Q8IXT5 | RB12B_HUMAN | RNA-binding protein 12B (RNA-binding motif protein 12B) |
| P30038 | AL4A1_HUMAN | Delta-1-pyrroline-5-carboxylate dehydrogenase, mitochondrial (P5C dehydrogenase) (EC 1.2.1.88) (Aldehyde dehydrogenase family 4 member A1) (L-glutamate gamma-semialdehyde dehydrogenase) |
| A0A024R4E5 | A0A024R4E5_HUMAN | High density lipoprotein binding protein (Vigilin), isoform CRA_a (Vigilin) |
| D6RBK0 | D6RBK0_HUMAN | Prohibitin (Fragment) |
| Q9H2X9 | S12A5_HUMAN | Solute carrier family 12 member 5 (Electroneutral potassium-chloride cotransporter 2) (K-Cl cotransporter 2) (hKCC2) (Neuronal K-Cl cotransporter) |
| Q9UI15 | TAGL3_HUMAN | Transgelin-3 (Neuronal protein 22) (NP22) (Neuronal protein NP25) |
| P11177 | ODPB_HUMAN | Pyruvate dehydrogenase E1 component subunit beta, mitochondrial (PDHE1-B) (EC 1.2.4.1) |
| P46779 | RL28_HUMAN | 60S ribosomal protein L28 (Large ribosomal subunit protein eL28) |
| O95674 | CDS2_HUMAN | Phosphatidate cytidylyltransferase 2 (EC 2.7.7.41) (CDP-DAG synthase 2) (CDP-DG synthase 2) (CDP-diacylglycerol synthase 2) (CDS 2) (CDP-diglyceride pyrophosphorylase 2) (CDP-diglyceride synthase 2) (CTP:phosphatidate cytidylyltransferase 2) |
| O43809 | CPSF5_HUMAN | Cleavage and polyadenylation specificity factor subunit 5 (Cleavage and polyadenylation specificity factor 25 kDa subunit) (CFIm25) (CPSF 25 kDa subunit) (Nucleoside diphosphate-linked moiety X motif 21) (Nudix motif 21) (Pre-mRNA cleavage factor Im 25 kDa subunit) |
| Q96GD0 | PLPP_HUMAN | Pyridoxal phosphate phosphatase (PLP phosphatase) (EC 3.1.3.3) (EC 3.1.3.74) (Chronophin) |
| Q9NY47 | CA2D2_HUMAN | Voltage-dependent calcium channel subunit alpha-2/delta-2 (Voltage-gated calcium channel subunit alpha-2/delta-2) [Cleaved into: Voltage-dependent calcium channel subunit alpha-2-2; Voltage-dependent calcium channel subunit delta-2] |
| Q9UM22 | EPDR1_HUMAN | Mammalian ependymin-related protein 1 (MERP-1) (Upregulated in colorectal cancer gene 1 protein) |
| P36575 | ARRC_HUMAN | Arrestin-C (Cone arrestin) (C-arrestin) (cArr) (Retinal cone arrestin-3) (X-arrestin) |
| Q8TBC4 | UBA3_HUMAN | NEDD8-activating enzyme E1 catalytic subunit (EC 6.3.2.-) (NEDD8-activating enzyme E1C) (Ubiquitin-activating enzyme E1C) (Ubiquitin-like modifier-activating enzyme 3) (Ubiquitin-activating enzyme 3) |
| O95373 | IPO7_HUMAN | Importin-7 (Imp7) (Ran-binding protein 7) (RanBP7) |
| Q66K74 | MAP1S_HUMAN | Microtubule-associated protein 1S (MAP-1S) (BPY2-interacting protein 1) (Microtubule-associated protein 8) (Variable charge Y chromosome 2-interacting protein 1) (VCY2-interacting protein 1) (VCY2IP-1) [Cleaved into: MAP1S heavy chain; MAP1S light chain] |
| P16520 | GBB3_HUMAN | Guanine nucleotide-binding protein G(I)/G(S)/G(T) subunit beta-3 (Transducin beta chain 3) |
| P04216 | THY1_HUMAN | Thy-1 membrane glycoprotein (CDw90) (Thy-1 antigen) (CD antigen CD90) |
| A0A0A0MRA8 | A0A0A0MRA8_HUMAN | Band 4.1-like protein 3 |
| A0A0A0MSA4 | A0A0A0MSA4_HUMAN | Band 4.1-like protein 3 |
| P08247 | SYPH_HUMAN | Synaptophysin (Major synaptic vesicle protein p38) |
| Q14318 | FKBP8_HUMAN | Peptidyl-prolyl cis-trans isomerase FKBP8 (PPIase FKBP8) (EC 5.2.1.8) (38 kDa FK506-binding protein) (38 kDa FKBP) (FKBP-38) (hFKBP38) (FK506-binding protein 8) (FKBP-8) (FKBPR38) (Rotamase) |
| P07305 | H10_HUMAN | Histone H1.0 (Histone H1') (Histone H1(0)) [Cleaved into: Histone H1.0, N-terminally processed] |
| Q9BXK5 | B2L13_HUMAN | Bcl-2-like protein 13 (Bcl2-L-13) (Bcl-rambo) (Protein Mil1) |
| Q14008 | CKAP5_HUMAN | Cytoskeleton-associated protein 5 (Colonic and hepatic tumor overexpressed gene protein) (Ch-TOG) |
| Q13131 | AAPK1_HUMAN | 5'-AMP-activated protein kinase catalytic subunit alpha-1 (AMPK subunit alpha-1) (EC 2.7.11.1) (Acetyl-CoA carboxylase kinase) (ACACA kinase) (EC 2.7.11.27) (Hydroxymethylglutaryl-CoA reductase kinase) (HMGCR kinase) (EC 2.7.11.31) (Tau-protein kinase PRKAA1) (EC 2.7.11.26) |
| Q2M2I8 | AAK1_HUMAN | AP2-associated protein kinase 1 (EC 2.7.11.1) (Adaptor-associated kinase 1) |
| O60506 | HNRPQ_HUMAN | Heterogeneous nuclear ribonucleoprotein Q (hnRNP Q) (Glycine- and tyrosine-rich RNA-binding protein) (GRY-RBP) (NS1-associated protein 1) (Synaptotagmin-binding, cytoplasmic RNA-interacting protein) |
| P54687 | BCAT1_HUMAN | Branched-chain-amino-acid aminotransferase, cytosolic (BCAT(c)) (EC 2.6.1.42) (Protein ECA39) |
| P61158 | ARP3_HUMAN | Actin-related protein 3 (Actin-like protein 3) |
| P04181 | OAT_HUMAN | Ornithine aminotransferase, mitochondrial (EC 2.6.1.13) (Ornithine delta-aminotransferase) (Ornithine--oxo-acid aminotransferase) [Cleaved into: Ornithine aminotransferase, hepatic form; Ornithine aminotransferase, renal form] |
| O00159 | MYO1C_HUMAN | Unconventional myosin-Ic (Myosin I beta) (MMI-beta) (MMIb) |
| P36542 | ATPG_HUMAN | ATP synthase subunit gamma, mitochondrial (F-ATPase gamma subunit) |
| Q15386 | UBE3C_HUMAN | Ubiquitin-protein ligase E3C (EC 2.3.2.26) (HECT-type ubiquitin transferase E3C) (HectH2) |
| Q9ULD0 | OGDHL_HUMAN | 2-oxoglutarate dehydrogenase-like, mitochondrial (EC 1.2.4.-) (2-oxoglutarate dehydrogenase complex component E1-like) (OGDC-E1-like) (Alpha-ketoglutarate dehydrogenase-like) |
| P21796 | VDAC1_HUMAN | Voltage-dependent anion-selective channel protein 1 (VDAC-1) (hVDAC1) (Outer mitochondrial membrane protein porin 1) (Plasmalemmal porin) (Porin 31HL) (Porin 31HM) |
| J3KPX7 | J3KPX7_HUMAN | Prohibitin-2 |
| P56134 | ATPK_HUMAN | ATP synthase subunit f, mitochondrial |
| P48681 | NEST_HUMAN | Nestin |
| P62314 | SMD1_HUMAN | Small nuclear ribonucleoprotein Sm D1 (Sm-D1) (Sm-D autoantigen) (snRNP core protein D1) |
| Q5VV89 | Q5VV89_HUMAN | Microsomal glutathione S-transferase 3 |
| Q00610 | CLH1_HUMAN | Clathrin heavy chain 1 (Clathrin heavy chain on chromosome 17) (CLH-17) |
| P00403 | COX2_HUMAN | Cytochrome c oxidase subunit 2 (Cytochrome c oxidase polypeptide II) |
| Q9UJS0 | CMC2_HUMAN | Calcium-binding mitochondrial carrier protein Aralar2 (Citrin) (Mitochondrial aspartate glutamate carrier 2) (Solute carrier family 25 member 13) |
| C9JD32 | C9JD32_HUMAN | 60S ribosomal protein L23 (Fragment) |
| P08195 | 4F2_HUMAN | 4F2 cell-surface antigen heavy chain (4F2hc) (4F2 heavy chain antigen) (Lymphocyte activation antigen 4F2 large subunit) (Solute carrier family 3 member 2) (CD antigen CD98) |
| P10515 | ODP2_HUMAN | Dihydrolipoyllysine-residue acetyltransferase component of pyruvate dehydrogenase complex, mitochondrial (EC 2.3.1.12) (70 kDa mitochondrial autoantigen of primary biliary cirrhosis) (PBC) (Dihydrolipoamide acetyltransferase component of pyruvate dehydrogenase complex) (M2 antigen complex 70 kDa subunit) (Pyruvate dehydrogenase complex component E2) (PDC-E2) (PDCE2) |
| Q02978 | M2OM_HUMAN | Mitochondrial 2-oxoglutarate/malate carrier protein (OGCP) (Solute carrier family 25 member 11) |
| O75489 | NDUS3_HUMAN | NADH dehydrogenase [ubiquinone] iron-sulfur protein 3, mitochondrial (EC 1.6.5.3) (EC 1.6.99.3) (Complex I-30kD) (CI-30kD) (NADH-ubiquinone oxidoreductase 30 kDa subunit) |
| P19367 | HXK1_HUMAN | Hexokinase-1 (EC 2.7.1.1) (Brain form hexokinase) (Hexokinase type I) (HK I) |
| Q9UQ03 | COR2B_HUMAN | Coronin-2B (Coronin-like protein C) (Clipin-C) (Protein FC96) |
| Q92990 | GLMN_HUMAN | Glomulin (FK506-binding protein-associated protein) (FAP) (FKBP-associated protein) |
| Q9NZN4 | EHD2_HUMAN | EH domain-containing protein 2 (PAST homolog 2) |
| Q7Z6Z7 | HUWE1_HUMAN | E3 ubiquitin-protein ligase HUWE1 (EC 2.3.2.26) (ARF-binding protein 1) (ARF-BP1) (HECT, UBA and WWE domain-containing protein 1) (HECT-type E3 ubiquitin transferase HUWE1) (Homologous to E6AP carboxyl terminus homologous protein 9) (HectH9) (Large structure of UREB1) (LASU1) (Mcl-1 ubiquitin ligase E3) (Mule) (Upstream regulatory element-binding protein 1) (URE-B1) (URE-binding protein 1) |
| Q8TDJ6 | DMXL2_HUMAN | DmX-like protein 2 (Rabconnectin-3) |
| Q9NXG2 | THUM1_HUMAN | THUMP domain-containing protein 1 |
| Q07021 | C1QBP_HUMAN | Complement component 1 Q subcomponent-binding protein, mitochondrial (ASF/SF2-associated protein p32) (Glycoprotein gC1qBP) (C1qBP) (Hyaluronan-binding protein 1) (Mitochondrial matrix protein p32) (gC1q-R protein) (p33) |
| H9KV28 | H9KV28_HUMAN | Protein diaphanous homolog 1 |
| P05388 | RLA0_HUMAN | 60S acidic ribosomal protein P0 (60S ribosomal protein L10E) (Large ribosomal subunit protein uL10) |
| P83731 | RL24_HUMAN | 60S ribosomal protein L24 (60S ribosomal protein L30) (Large ribosomal subunit protein eL24) |
| O43681 | ASNA_HUMAN | ATPase ASNA1 (EC 3.6.-.-) (Arsenical pump-driving ATPase) (Arsenite-stimulated ATPase) (Transmembrane domain recognition complex 40 kDa ATPase subunit) (hARSA-I) (hASNA-I) |
| Q9UI12 | VATH_HUMAN | V-type proton ATPase subunit H (V-ATPase subunit H) (Nef-binding protein 1) (NBP1) (Protein VMA13 homolog) (V-ATPase 50/57 kDa subunits) (Vacuolar proton pump subunit H) (Vacuolar proton pump subunit SFD) |
| Q9NS69 | TOM22_HUMAN | Mitochondrial import receptor subunit TOM22 homolog (hTom22) (1C9-2) (Translocase of outer membrane 22 kDa subunit homolog) |
| P61247 | RS3A_HUMAN | 40S ribosomal protein S3a (Small ribosomal subunit protein eS1) (v-fos transformation effector protein) (Fte-1) |
| P49748 | ACADV_HUMAN | Very long-chain specific acyl-CoA dehydrogenase, mitochondrial (VLCAD) (EC 1.3.8.9) |
| O75390 | CISY_HUMAN | Citrate synthase, mitochondrial (EC 2.3.3.1) (Citrate (Si)-synthase) |
| Q9UBB6 | NCDN_HUMAN | Neurochondrin |
| Q7Z460 | CLAP1_HUMAN | CLIP-associating protein 1 (Cytoplasmic linker-associated protein 1) (Multiple asters homolog 1) (Protein Orbit homolog 1) (hOrbit1) |
| P08238 | HS90B_HUMAN | Heat shock protein HSP 90-beta (HSP 90) (Heat shock 84 kDa) (HSP 84) (HSP84) |
| Q92896 | GSLG1_HUMAN | Golgi apparatus protein 1 (CFR-1) (Cysteine-rich fibroblast growth factor receptor) (E-selectin ligand 1) (ESL-1) (Golgi sialoglycoprotein MG-160) |
| O43148 | MCES_HUMAN | mRNA cap guanine-N7 methyltransferase (EC 2.1.1.56) (RG7MT1) (mRNA (guanine-N(7)-)-methyltransferase) (mRNA cap methyltransferase) (hCMT1) (hMet) (hcm1p) |
| Q8TBG9 | SYNPR_HUMAN | Synaptoporin |
| Q9NSD9 | SYFB_HUMAN | Phenylalanine--tRNA ligase beta subunit (EC 6.1.1.20) (Phenylalanyl-tRNA synthetase beta subunit) (PheRS) |
| P62760 | VISL1_HUMAN | Visinin-like protein 1 (VILIP) (VLP-1) (Hippocalcin-like protein 3) (HLP3) |
| P53367 | ARFP1_HUMAN | Arfaptin-1 (ADP-ribosylation factor-interacting protein 1) |
| Q15067 | ACOX1_HUMAN | Peroxisomal acyl-coenzyme A oxidase 1 (AOX) (EC 1.3.3.6) (Palmitoyl-CoA oxidase) (Straight-chain acyl-CoA oxidase) (SCOX) |
| P54709 | AT1B3_HUMAN | Sodium/potassium-transporting ATPase subunit beta-3 (Sodium/potassium-dependent ATPase subunit beta-3) (ATPB-3) (CD antigen CD298) |
| Q13155 | AIMP2_HUMAN | Aminoacyl tRNA synthase complex-interacting multifunctional protein 2 (Multisynthase complex auxiliary component p38) (Protein JTV-1) |
| Q6IBS0 | TWF2_HUMAN | Twinfilin-2 (A6-related protein) (hA6RP) (Protein tyrosine kinase 9-like) (Twinfilin-1-like protein) |
| Q96QR8 | PURB_HUMAN | Transcriptional activator protein Pur-beta (Purine-rich element-binding protein B) |
| P23396 | RS3_HUMAN | 40S ribosomal protein S3 (EC 4.2.99.18) (Small ribosomal subunit protein uS3) |
| O75964 | ATP5L_HUMAN | ATP synthase subunit g, mitochondrial (ATPase subunit g) |
| P59998 | ARPC4_HUMAN | Actin-related protein 2/3 complex subunit 4 (Arp2/3 complex 20 kDa subunit) (p20-ARC) |
| O00154 | BACH_HUMAN | Cytosolic acyl coenzyme A thioester hydrolase (EC 3.1.2.2) (Acyl-CoA thioesterase 7) (Brain acyl-CoA hydrolase) (BACH) (CTE-IIa) (CTE-II) (Long chain acyl-CoA thioester hydrolase) |
| J3KN01 | J3KN01_HUMAN | Afadin |
| P05198 | IF2A_HUMAN | Eukaryotic translation initiation factor 2 subunit 1 (Eukaryotic translation initiation factor 2 subunit alpha) (eIF-2-alpha) (eIF-2A) (eIF-2alpha) |
| P62330 | ARF6_HUMAN | ADP-ribosylation factor 6 |
| Q9H0B6 | KLC2_HUMAN | Kinesin light chain 2 (KLC 2) |
| P61313 | RL15_HUMAN | 60S ribosomal protein L15 (Large ribosomal subunit protein eL15) |
| Q14576 | ELAV3_HUMAN | ELAV-like protein 3 (Hu-antigen C) (HuC) (Paraneoplastic cerebellar degeneration-associated antigen) (Paraneoplastic limbic encephalitis antigen 21) |
| O43617 | TPPC3_HUMAN | Trafficking protein particle complex subunit 3 (BET3 homolog) |
| O94925 | GLSK_HUMAN | Glutaminase kidney isoform, mitochondrial (GLS) (EC 3.5.1.2) (K-glutaminase) (L-glutamine amidohydrolase) |
| P30049 | ATPD_HUMAN | ATP synthase subunit delta, mitochondrial (F-ATPase delta subunit) |
| Q15813 | TBCE_HUMAN | Tubulin-specific chaperone E (Tubulin-folding cofactor E) |
| P41252 | SYIC_HUMAN | Isoleucine--tRNA ligase, cytoplasmic (EC 6.1.1.5) (Isoleucyl-tRNA synthetase) (IRS) (IleRS) |
| P61204 | ARF3_HUMAN | ADP-ribosylation factor 3 |
| O00468 | AGRIN_HUMAN | Agrin [Cleaved into: Agrin N-terminal 110 kDa subunit; Agrin C-terminal 110 kDa subunit; Agrin C-terminal 90 kDa fragment (C90); Agrin C-terminal 22 kDa fragment (C22)] |
| O75122 | CLAP2_HUMAN | CLIP-associating protein 2 (Cytoplasmic linker-associated protein 2) (Protein Orbit homolog 2) (hOrbit2) |
| Q14204 | DYHC1_HUMAN | Cytoplasmic dynein 1 heavy chain 1 (Cytoplasmic dynein heavy chain 1) (Dynein heavy chain, cytosolic) |
| O95782 | AP2A1_HUMAN | AP-2 complex subunit alpha-1 (100 kDa coated vesicle protein A) (Adaptor protein complex AP-2 subunit alpha-1) (Adaptor-related protein complex 2 subunit alpha-1) (Alpha-adaptin A) (Alpha1-adaptin) (Clathrin assembly protein complex 2 alpha-A large chain) (Plasma membrane adaptor HA2/AP2 adaptin alpha A subunit) |
| Q9UDW1 | QCR9_HUMAN | Cytochrome b-c1 complex subunit 9 (Complex III subunit 9) (Complex III subunit X) (Cytochrome c1 non-heme 7 kDa protein) (Ubiquinol-cytochrome c reductase complex 7.2 kDa protein) |
| P55010 | IF5_HUMAN | Eukaryotic translation initiation factor 5 (eIF-5) |
| Q53GS9 | SNUT2_HUMAN | U4/U6.U5 tri-snRNP-associated protein 2 (Inactive ubiquitin-specific peptidase 39) (SAD1 homolog) (U4/U6.U5 tri-snRNP-associated 65 kDa protein) (65K) |
| P28074 | PSB5_HUMAN | Proteasome subunit beta type-5 (EC 3.4.25.1) (Macropain epsilon chain) (Multicatalytic endopeptidase complex epsilon chain) (Proteasome chain 6) (Proteasome epsilon chain) (Proteasome subunit MB1) (Proteasome subunit X) |
| Q01814 | AT2B2_HUMAN | Plasma membrane calcium-transporting ATPase 2 (PMCA2) (EC 3.6.3.8) (Plasma membrane calcium ATPase isoform 2) (Plasma membrane calcium pump isoform 2) |
| Q9BSJ8 | ESYT1_HUMAN | Extended synaptotagmin-1 (E-Syt1) (Membrane-bound C2 domain-containing protein) |
| P36915 | GNL1_HUMAN | Guanine nucleotide-binding protein-like 1 (GTP-binding protein HSR1) |
| P26639 | SYTC_HUMAN | Threonine--tRNA ligase, cytoplasmic (EC 6.1.1.3) (Threonyl-tRNA synthetase) (ThrRS) |
| Q13813 | SPTN1_HUMAN | Spectrin alpha chain, non-erythrocytic 1 (Alpha-II spectrin) (Fodrin alpha chain) (Spectrin, non-erythroid alpha subunit) |
| P50914 | RL14_HUMAN | 60S ribosomal protein L14 (CAG-ISL 7) (Large ribosomal subunit protein eL14) |
| Q9BWD1 | THIC_HUMAN | Acetyl-CoA acetyltransferase, cytosolic (EC 2.3.1.9) (Acetyl-CoA transferase-like protein) (Cytosolic acetoacetyl-CoA thiolase) |
| E9PGC8 | E9PGC8_HUMAN | Microtubule-associated protein 1A |
| P61106 | RAB14_HUMAN | Ras-related protein Rab-14 |
| Q9P0L0 | VAPA_HUMAN | Vesicle-associated membrane protein-associated protein A (VAMP-A) (VAMP-associated protein A) (VAP-A) (33 kDa VAMP-associated protein) (VAP-33) |
| Q9P2U7 | VGLU1_HUMAN | Vesicular glutamate transporter 1 (VGluT1) (Brain-specific Na(+)-dependent inorganic phosphate cotransporter) (Solute carrier family 17 member 7) |
| P51160 | PDE6C_HUMAN | Cone cGMP-specific 3',5'-cyclic phosphodiesterase subunit alpha' (EC 3.1.4.35) (cGMP phosphodiesterase 6C) |
| P20020 | AT2B1_HUMAN | Plasma membrane calcium-transporting ATPase 1 (PMCA1) (EC 3.6.3.8) (Plasma membrane calcium ATPase isoform 1) (Plasma membrane calcium pump isoform 1) |
| Q9Y639 | NPTN_HUMAN | Neuroplastin (Stromal cell-derived receptor 1) (SDR-1) |
| Q06278 | AOXA_HUMAN | Aldehyde oxidase (EC 1.2.3.1) (Aldehyde oxidase 1) (Azaheterocycle hydroxylase) (EC 1.17.3.-) |
| O95433 | AHSA1_HUMAN | Activator of 90 kDa heat shock protein ATPase homolog 1 (AHA1) (p38) |
| P20336 | RAB3A_HUMAN | Ras-related protein Rab-3A |
| O43301 | HS12A_HUMAN | Heat shock 70 kDa protein 12A |
| O94906 | PRP6_HUMAN | Pre-mRNA-processing factor 6 (Androgen receptor N-terminal domain-transactivating protein 1) (ANT-1) (PRP6 homolog) (U5 snRNP-associated 102 kDa protein) (U5-102 kDa protein) |
| P04275 | VWF_HUMAN | von Willebrand factor (vWF) [Cleaved into: von Willebrand antigen 2 (von Willebrand antigen II)] |
| Q8N573 | OXR1_HUMAN | Oxidation resistance protein 1 |
| O60282 | KIF5C_HUMAN | Kinesin heavy chain isoform 5C (Kinesin heavy chain neuron-specific 2) |
| J3KNA1 | J3KNA1_HUMAN | Kinesin-like protein |
| P53677 | AP3M2_HUMAN | AP-3 complex subunit mu-2 (Adaptor-related protein complex 3 subunit mu-2) (Clathrin assembly protein assembly protein complex 3 mu-2 medium chain) (Clathrin coat assembly protein AP47 homolog 2) (Clathrin coat-associated protein AP47 homolog 2) (Golgi adaptor AP-1 47 kDa protein homolog 2) (HA1 47 kDa subunit homolog 2) (Mu3B-adaptin) (P47B) |
| P35268 | RL22_HUMAN | 60S ribosomal protein L22 (EBER-associated protein) (EAP) (Epstein-Barr virus small RNA-associated protein) (Heparin-binding protein HBp15) (Large ribosomal subunit protein eL22) |
| P46821 | MAP1B_HUMAN | Microtubule-associated protein 1B (MAP-1B) [Cleaved into: MAP1B heavy chain; MAP1 light chain LC1] |
| Q9UBC2 | EP15R_HUMAN | Epidermal growth factor receptor substrate 15-like 1 (Eps15-related protein) (Eps15R) |
| P46379 | BAG6_HUMAN | Large proline-rich protein BAG6 (BAG family molecular chaperone regulator 6) (BCL2-associated athanogene 6) (BAG-6) (HLA-B-associated transcript 3) (Protein G3) (Protein Scythe) |
| P21579 | SYT1_HUMAN | Synaptotagmin-1 (Synaptotagmin I) (SytI) (p65) |
| P05023 | AT1A1_HUMAN | Sodium/potassium-transporting ATPase subunit alpha-1 (Na(+)/K(+) ATPase alpha-1 subunit) (EC 3.6.3.9) (Sodium pump subunit alpha-1) |
| P31153 | METK2_HUMAN | S-adenosylmethionine synthase isoform type-2 (AdoMet synthase 2) (EC 2.5.1.6) (Methionine adenosyltransferase 2) (MAT 2) (Methionine adenosyltransferase II) (MAT-II) |
| Q02790 | FKBP4_HUMAN | Peptidyl-prolyl cis-trans isomerase FKBP4 (PPIase FKBP4) (EC 5.2.1.8) (51 kDa FK506-binding protein) (FKBP51) (52 kDa FK506-binding protein) (52 kDa FKBP) (FKBP-52) (59 kDa immunophilin) (p59) (FK506-binding protein 4) (FKBP-4) (FKBP59) (HSP-binding immunophilin) (HBI) (Immunophilin FKBP52) (Rotamase) [Cleaved into: Peptidyl-prolyl cis-trans isomerase FKBP4, N-terminally processed] |
| P49840 | GSK3A_HUMAN | Glycogen synthase kinase-3 alpha (GSK-3 alpha) (EC 2.7.11.26) (Serine/threonine-protein kinase GSK3A) (EC 2.7.11.1) |
| Q9BUF5 | TBB6_HUMAN | Tubulin beta-6 chain (Tubulin beta class V) |
| Q01082 | SPTB2_HUMAN | Spectrin beta chain, non-erythrocytic 1 (Beta-II spectrin) (Fodrin beta chain) (Spectrin, non-erythroid beta chain 1) |
| P51674 | GPM6A_HUMAN | Neuronal membrane glycoprotein M6-a (M6a) |
| O00519 | FAAH1_HUMAN | Fatty-acid amide hydrolase 1 (EC 3.5.1.99) (Anandamide amidohydrolase 1) (Oleamide hydrolase 1) |
| Q12904 | AIMP1_HUMAN | Aminoacyl tRNA synthase complex-interacting multifunctional protein 1 (Multisynthase complex auxiliary component p43) [Cleaved into: Endothelial monocyte-activating polypeptide 2 (EMAP-2) (Endothelial monocyte-activating polypeptide II) (EMAP-II) (Small inducible cytokine subfamily E member 1)] |
| Q9Y3D6 | FIS1_HUMAN | Mitochondrial fission 1 protein (FIS1 homolog) (hFis1) (Tetratricopeptide repeat protein 11) (TPR repeat protein 11) |
| P61764 | STXB1_HUMAN | Syntaxin-binding protein 1 (MUNC18-1) (N-Sec1) (Protein unc-18 homolog 1) (Unc18-1) (Protein unc-18 homolog A) (Unc-18A) (p67) |
| P48449 | ERG7_HUMAN | Lanosterol synthase (EC 5.4.99.7) (2,3-epoxysqualene--lanosterol cyclase) (Oxidosqualene--lanosterol cyclase) (OSC) (hOSC) |
| P07814 | SYEP_HUMAN | Bifunctional glutamate/proline--tRNA ligase (Bifunctional aminoacyl-tRNA synthetase) (Cell proliferation-inducing gene 32 protein) (Glutamatyl-prolyl-tRNA synthetase) [Includes: Glutamate--tRNA ligase (EC 6.1.1.17) (Glutamyl-tRNA synthetase) (GluRS); Proline--tRNA ligase (EC 6.1.1.15) (Prolyl-tRNA synthetase)] |
| Q8WUK0 | PTPM1_HUMAN | Phosphatidylglycerophosphatase and protein-tyrosine phosphatase 1 (EC 3.1.3.27) (PTEN-like phosphatase) (Phosphoinositide lipid phosphatase) (Protein-tyrosine phosphatase mitochondrial 1) (EC 3.1.3.16) (EC 3.1.3.48) |
| Q13595 | TRA2A_HUMAN | Transformer-2 protein homolog alpha (TRA-2 alpha) (TRA2-alpha) (Transformer-2 protein homolog A) |
| Q86X55 | CARM1_HUMAN | Histone-arginine methyltransferase CARM1 (EC 2.1.1.319) (Coactivator-associated arginine methyltransferase 1) (Protein arginine N-methyltransferase 4) |
| Q15555 | MARE2_HUMAN | Microtubule-associated protein RP/EB family member 2 (APC-binding protein EB2) (End-binding protein 2) (EB2) |
| P12036 | NFH_HUMAN | Neurofilament heavy polypeptide (NF-H) (200 kDa neurofilament protein) (Neurofilament triplet H protein) |
| P07954 | FUMH_HUMAN | Fumarate hydratase, mitochondrial (Fumarase) (EC 4.2.1.2) |
| Q13423 | NNTM_HUMAN | NAD(P) transhydrogenase, mitochondrial (EC 1.6.1.2) (Nicotinamide nucleotide transhydrogenase) (Pyridine nucleotide transhydrogenase) |
| Q15436 | SC23A_HUMAN | Protein transport protein Sec23A (SEC23-related protein A) |
| Q9NY65 | TBA8_HUMAN | Tubulin alpha-8 chain (Alpha-tubulin 8) (Tubulin alpha chain-like 2) |
| P12271 | RLBP1_HUMAN | Retinaldehyde-binding protein 1 (Cellular retinaldehyde-binding protein) |
| P00441 | SODC_HUMAN | Superoxide dismutase [Cu-Zn] (EC 1.15.1.1) (Superoxide dismutase 1) (hSod1) |
| O43186 | CRX_HUMAN | Cone-rod homeobox protein |
| P37108 | SRP14_HUMAN | Signal recognition particle 14 kDa protein (SRP14) (18 kDa Alu RNA-binding protein) |
| P63241 | IF5A1_HUMAN | Eukaryotic translation initiation factor 5A-1 (eIF-5A-1) (eIF-5A1) (Eukaryotic initiation factor 5A isoform 1) (eIF-5A) (Rev-binding factor) (eIF-4D) |
| O14558 | HSPB6_HUMAN | Heat shock protein beta-6 (HspB6) (Heat shock 20 kDa-like protein p20) |
| P60981 | DEST_HUMAN | Destrin (Actin-depolymerizing factor) (ADF) |
| O15145 | ARPC3_HUMAN | Actin-related protein 2/3 complex subunit 3 (Arp2/3 complex 21 kDa subunit) (p21-ARC) |
| Q8NF37 | PCAT1_HUMAN | Lysophosphatidylcholine acyltransferase 1 (LPC acyltransferase 1) (LPCAT-1) (LysoPC acyltransferase 1) (EC 2.3.1.23) (1-acylglycerophosphocholine O-acyltransferase) (1-alkylglycerophosphocholine O-acetyltransferase) (EC 2.3.1.67) (Acetyl-CoA:lyso-platelet-activating factor acetyltransferase) (Acetyl-CoA:lyso-PAF acetyltransferase) (Lyso-PAF acetyltransferase) (LysoPAFAT) (Acyltransferase-like 2) (Phosphonoformate immuno-associated protein 3) |
| Q9Y2X3 | NOP58_HUMAN | Nucleolar protein 58 (Nucleolar protein 5) |
| P55084 | ECHB_HUMAN | Trifunctional enzyme subunit beta, mitochondrial (TP-beta) [Includes: 3-ketoacyl-CoA thiolase (EC 2.3.1.16) (Acetyl-CoA acyltransferase) (Beta-ketothiolase)] |
| Q96F85 | CNRP1_HUMAN | CB1 cannabinoid receptor-interacting protein 1 (CRIP-1) |
| P30044 | PRDX5_HUMAN | Peroxiredoxin-5, mitochondrial (EC 1.11.1.15) (Alu corepressor 1) (Antioxidant enzyme B166) (AOEB166) (Liver tissue 2D-page spot 71B) (PLP) (Peroxiredoxin V) (Prx-V) (Peroxisomal antioxidant enzyme) (TPx type VI) (Thioredoxin peroxidase PMP20) (Thioredoxin reductase) |
| Q96P70 | IPO9_HUMAN | Importin-9 (Imp9) (Ran-binding protein 9) (RanBP9) |
| P62873 | GBB1_HUMAN | Guanine nucleotide-binding protein G(I)/G(S)/G(T) subunit beta-1 (Transducin beta chain 1) |
| P37837 | TALDO_HUMAN | Transaldolase (EC 2.2.1.2) |
| Q9HC38 | GLOD4_HUMAN | Glyoxalase domain-containing protein 4 |
| F8WE04 | F8WE04_HUMAN | Heat shock protein beta-1 |
| P04792 | HSPB1_HUMAN | Heat shock protein beta-1 (HspB1) (28 kDa heat shock protein) (Estrogen-regulated 24 kDa protein) (Heat shock 27 kDa protein) (HSP 27) (Stress-responsive protein 27) (SRP27) |
| P08100 | OPSD_HUMAN | Rhodopsin (Opsin-2) |
| P11488 | GNAT1_HUMAN | Guanine nucleotide-binding protein G(t) subunit alpha-1 (Transducin alpha-1 chain) |
| Q15121 | PEA15_HUMAN | Astrocytic phosphoprotein PEA-15 (15 kDa phosphoprotein enriched in astrocytes) (Phosphoprotein enriched in diabetes) (PED) |
| Q10713 | MPPA_HUMAN | Mitochondrial-processing peptidase subunit alpha (EC 3.4.24.64) (Alpha-MPP) (P-55) |
| O15540 | FABP7_HUMAN | Fatty acid-binding protein, brain (Brain lipid-binding protein) (BLBP) (Brain-type fatty acid-binding protein) (B-FABP) (Fatty acid-binding protein 7) (Mammary-derived growth inhibitor related) |
| P02489 | CRYAA_HUMAN | Alpha-crystallin A chain (Heat shock protein beta-4) (HspB4) [Cleaved into: Alpha-crystallin A(1-172); Alpha-crystallin A(1-168); Alpha-crystallin A(1-162)] |
| O60721 | NCKX1_HUMAN | Sodium/potassium/calcium exchanger 1 (Na(+)/K(+)/Ca(2+)-exchange protein 1) (Retinal rod Na-Ca+K exchanger) (Solute carrier family 24 member 1) |
| P78363 | ABCA4_HUMAN | Retinal-specific ATP-binding cassette transporter (ATP-binding cassette sub-family A member 4) (RIM ABC transporter) (RIM protein) (RmP) (Stargardt disease protein) |
| O00264 | PGRC1_HUMAN | Membrane-associated progesterone receptor component 1 (mPR) |
| Q9NZA1 | CLIC5_HUMAN | Chloride intracellular channel protein 5 |
| Q9BYZ2 | LDH6B_HUMAN | L-lactate dehydrogenase A-like 6B (EC 1.1.1.27) |
| P52597 | HNRPF_HUMAN | Heterogeneous nuclear ribonucleoprotein F (hnRNP F) (Nucleolin-like protein mcs94-1) [Cleaved into: Heterogeneous nuclear ribonucleoprotein F, N-terminally processed] |
| P31942 | HNRH3_HUMAN | Heterogeneous nuclear ribonucleoprotein H3 (hnRNP H3) (Heterogeneous nuclear ribonucleoprotein 2H9) (hnRNP 2H9) |
| P19404 | NDUV2_HUMAN | NADH dehydrogenase [ubiquinone] flavoprotein 2, mitochondrial (EC 1.6.5.3) (EC 1.6.99.3) (NADH-ubiquinone oxidoreductase 24 kDa subunit) |
| P16403 | H12_HUMAN | Histone H1.2 (Histone H1c) (Histone H1d) (Histone H1s-1) |
| Q9H0A0 | NAT10_HUMAN | RNA cytidine acetyltransferase (EC 2.3.1.-) (18S rRNA cytosine acetyltransferase) (N-acetyltransferase 10) |
| Q9UK41 | VPS28_HUMAN | Vacuolar protein sorting-associated protein 28 homolog (H-Vps28) (ESCRT-I complex subunit VPS28) |
| A0A1X7SBS1 | A0A1X7SBS1_HUMAN | Heterogeneous nuclear ribonucleoprotein U |
| Q09666 | AHNK_HUMAN | Neuroblast differentiation-associated protein AHNAK (Desmoyokin) |
| P14927 | QCR7_HUMAN | Cytochrome b-c1 complex subunit 7 (Complex III subunit 7) (Complex III subunit VII) (QP-C) (Ubiquinol-cytochrome c reductase complex 14 kDa protein) |
| P49189 | AL9A1_HUMAN | 4-trimethylaminobutyraldehyde dehydrogenase (TMABADH) (EC 1.2.1.47) (Aldehyde dehydrogenase E3 isozyme) (Aldehyde dehydrogenase family 9 member A1) (EC 1.2.1.3) (Gamma-aminobutyraldehyde dehydrogenase) (EC 1.2.1.19) (R-aminobutyraldehyde dehydrogenase) [Cleaved into: 4-trimethylaminobutyraldehyde dehydrogenase, N-terminally processed] |
| P31930 | QCR1_HUMAN | Cytochrome b-c1 complex subunit 1, mitochondrial (Complex III subunit 1) (Core protein I) (Ubiquinol-cytochrome-c reductase complex core protein 1) |
| P28072 | PSB6_HUMAN | Proteasome subunit beta type-6 (EC 3.4.25.1) (Macropain delta chain) (Multicatalytic endopeptidase complex delta chain) (Proteasome delta chain) (Proteasome subunit Y) |
| P69891 | HBG1_HUMAN | Hemoglobin subunit gamma-1 (Gamma-1-globin) (Hb F Agamma) (Hemoglobin gamma-1 chain) (Hemoglobin gamma-A chain) |
| A0A0A0MRJ6 | A0A0A0MRJ6_HUMAN | Protein-L-isoaspartate O-methyltransferase (EC 2.1.1.77) |
| Q08752 | PPID_HUMAN | Peptidyl-prolyl cis-trans isomerase D (PPIase D) (EC 5.2.1.8) (40 kDa peptidyl-prolyl cis-trans isomerase) (Cyclophilin-40) (CYP-40) (Cyclophilin-related protein) (Rotamase D) |
| O75947 | ATP5H_HUMAN | ATP synthase subunit d, mitochondrial (ATPase subunit d) |
| P20674 | COX5A_HUMAN | Cytochrome c oxidase subunit 5A, mitochondrial (Cytochrome c oxidase polypeptide Va) |
| Q5SSJ5 | HP1B3_HUMAN | Heterochromatin protein 1-binding protein 3 (Protein HP1-BP74) |
| J3KN42 | J3KN42_HUMAN | Calcium-binding mitochondrial carrier protein SCaMC-1 |
| Q17R60 | IMPG1_HUMAN | Interphotoreceptor matrix proteoglycan 1 (Interphotoreceptor matrix proteoglycan of 150 kDa) (IPM-150) (Sialoprotein associated with cones and rods) |
| Q12874 | SF3A3_HUMAN | Splicing factor 3A subunit 3 (SF3a60) (Spliceosome-associated protein 61) (SAP 61) |
| P84243 | H33_HUMAN | Histone H3.3 |
| P54819 | KAD2_HUMAN | Adenylate kinase 2, mitochondrial (AK 2) (EC 2.7.4.3) (ATP-AMP transphosphorylase 2) (ATP:AMP phosphotransferase) (Adenylate monophosphate kinase) [Cleaved into: Adenylate kinase 2, mitochondrial, N-terminally processed] |
| P35612 | ADDB_HUMAN | Beta-adducin (Erythrocyte adducin subunit beta) |
| P58876 | H2B1D_HUMAN | Histone H2B type 1-D (HIRA-interacting protein 2) (Histone H2B.1 B) (Histone H2B.b) (H2B/b) |
| P55809 | SCOT1_HUMAN | Succinyl-CoA:3-ketoacid coenzyme A transferase 1, mitochondrial (EC 2.8.3.5) (3-oxoacid CoA-transferase 1) (Somatic-type succinyl-CoA:3-oxoacid CoA-transferase) (SCOT-s) |
| Q04760 | LGUL_HUMAN | Lactoylglutathione lyase (EC 4.4.1.5) (Aldoketomutase) (Glyoxalase I) (Glx I) (Ketone-aldehyde mutase) (Methylglyoxalase) (S-D-lactoylglutathione methylglyoxal lyase) |
| P17066 | HSP76_HUMAN | Heat shock 70 kDa protein 6 (Heat shock 70 kDa protein B') |
| P54652 | HSP72_HUMAN | Heat shock-related 70 kDa protein 2 (Heat shock 70 kDa protein 2) |
| P10523 | ARRS_HUMAN | S-arrestin (48 kDa protein) (Retinal S-antigen) (S-AG) (Rod photoreceptor arrestin) |
| P49458 | SRP09_HUMAN | Signal recognition particle 9 kDa protein (SRP9) |
| Q8WXX5 | DNJC9_HUMAN | DnaJ homolog subfamily C member 9 (HDJC9) (DnaJ protein SB73) |
| Q96PM9 | Z385A_HUMAN | Zinc finger protein 385A (Hematopoietic zinc finger protein) (Retinal zinc finger protein) |
| B1AK87 | B1AK87_HUMAN | Capping protein (Actin filament) muscle Z-line, beta, isoform CRA_a (F-actin-capping protein subunit beta) |
| O96000 | NDUBA_HUMAN | NADH dehydrogenase [ubiquinone] 1 beta subcomplex subunit 10 (Complex I-PDSW) (CI-PDSW) (NADH-ubiquinone oxidoreductase PDSW subunit) |
| Q9P121 | NTRI_HUMAN | Neurotrimin (hNT) (IgLON family member 2) |
| Q14028 | CNGB1_HUMAN | Cyclic nucleotide-gated cation channel beta-1 (Cyclic nucleotide-gated cation channel 4) (CNG channel 4) (CNG-4) (CNG4) (Cyclic nucleotide-gated cation channel gamma) (Cyclic nucleotide-gated cation channel modulatory subunit) (Cyclic nucleotide-gated channel beta-1) (CNG channel beta-1) (Glutamic acid-rich protein) (GARP) |
| P10644 | KAP0_HUMAN | cAMP-dependent protein kinase type I-alpha regulatory subunit (Tissue-specific extinguisher 1) (TSE1) [Cleaved into: cAMP-dependent protein kinase type I-alpha regulatory subunit, N-terminally processed] |
| P09382 | LEG1_HUMAN | Galectin-1 (Gal-1) (14 kDa laminin-binding protein) (HLBP14) (14 kDa lectin) (Beta-galactoside-binding lectin L-14-I) (Galaptin) (HBL) (HPL) (Lactose-binding lectin 1) (Lectin galactoside-binding soluble 1) (Putative MAPK-activating protein PM12) (S-Lac lectin 1) |
| G8JLA2 | G8JLA2_HUMAN | Myosin light polypeptide 6 |
| Q9Y2W2 | WBP11_HUMAN | WW domain-binding protein 11 (WBP-11) (Npw38-binding protein) (NpwBP) (SH3 domain-binding protein SNP70) (Splicing factor that interacts with PQBP-1 and PP1) |
| O15230 | LAMA5_HUMAN | Laminin subunit alpha-5 (Laminin-10 subunit alpha) (Laminin-11 subunit alpha) (Laminin-15 subunit alpha) |
| P11142 | HSP7C_HUMAN | Heat shock cognate 71 kDa protein (Heat shock 70 kDa protein 8) (Lipopolysaccharide-associated protein 1) (LAP-1) (LPS-associated protein 1) |
| E7EN89 | E7EN89_HUMAN | Toll interacting protein, isoform CRA_b (Toll-interacting protein) |
| P60174 | TPIS_HUMAN | Triosephosphate isomerase (TIM) (EC 5.3.1.1) (Triose-phosphate isomerase) |
| Q7L0J3 | SV2A_HUMAN | Synaptic vesicle glycoprotein 2A |
| P32119 | PRDX2_HUMAN | Peroxiredoxin-2 (EC 1.11.1.15) (Natural killer cell-enhancing factor B) (NKEF-B) (PRP) (Thiol-specific antioxidant protein) (TSA) (Thioredoxin peroxidase 1) (Thioredoxin-dependent peroxide reductase 1) |
| J3KNF8 | J3KNF8_HUMAN | Cytochrome b5 type B (Cytochrome b5 type B (Outer mitochondrial membrane), isoform CRA_a) |
| Q06830 | PRDX1_HUMAN | Peroxiredoxin-1 (EC 1.11.1.15) (Natural killer cell-enhancing factor A) (NKEF-A) (Proliferation-associated gene protein) (PAG) (Thioredoxin peroxidase 2) (Thioredoxin-dependent peroxide reductase 2) |
| Q13162 | PRDX4_HUMAN | Peroxiredoxin-4 (EC 1.11.1.15) (Antioxidant enzyme AOE372) (AOE37-2) (Peroxiredoxin IV) (Prx-IV) (Thioredoxin peroxidase AO372) (Thioredoxin-dependent peroxide reductase A0372) |
| Q9Y2B0 | CNPY2_HUMAN | Protein canopy homolog 2 (MIR-interacting saposin-like protein) (Putative secreted protein Zsig9) (Transmembrane protein 4) |
| Q9UMX0 | UBQL1_HUMAN | Ubiquilin-1 (Protein linking IAP with cytoskeleton 1) (PLIC-1) (hPLIC-1) |
| A0A0A0MR02 | A0A0A0MR02_HUMAN | Voltage-dependent anion-selective channel protein 2 (Fragment) |
| P10412 | H14_HUMAN | Histone H1.4 (Histone H1b) (Histone H1s-4) |
| P34931 | HS71L_HUMAN | Heat shock 70 kDa protein 1-like (Heat shock 70 kDa protein 1L) (Heat shock 70 kDa protein 1-Hom) (HSP70-Hom) |
| Q92599 | SEPT8_HUMAN | Septin-8 |
| Q96AE4 | FUBP1_HUMAN | Far upstream element-binding protein 1 (FBP) (FUSE-binding protein 1) (DNA helicase V) (hDH V) |
| P0DMV9 | HS71B_HUMAN | Heat shock 70 kDa protein 1B (Heat shock 70 kDa protein 2) (HSP70-2) (HSP70.2) |
| Q5TZA2 | CROCC_HUMAN | Rootletin (Ciliary rootlet coiled-coil protein) |
| P28838 | AMPL_HUMAN | Cytosol aminopeptidase (EC 3.4.11.1) (Leucine aminopeptidase 3) (LAP-3) (Leucyl aminopeptidase) (Peptidase S) (Proline aminopeptidase) (EC 3.4.11.5) (Prolyl aminopeptidase) |
| P07197 | NFM_HUMAN | Neurofilament medium polypeptide (NF-M) (160 kDa neurofilament protein) (Neurofilament 3) (Neurofilament triplet M protein) |
| Q16352 | AINX_HUMAN | Alpha-internexin (Alpha-Inx) (66 kDa neurofilament protein) (NF-66) (Neurofilament-66) (Neurofilament 5) |
| O94760 | DDAH1_HUMAN | N(G),N(G)-dimethylarginine dimethylaminohydrolase 1 (DDAH-1) (Dimethylarginine dimethylaminohydrolase 1) (EC 3.5.3.18) (DDAHI) (Dimethylargininase-1) |
| P15104 | GLNA_HUMAN | Glutamine synthetase (GS) (EC 6.3.1.2) (Glutamate decarboxylase) (EC 4.1.1.15) (Glutamate--ammonia ligase) |
| P35611 | ADDA_HUMAN | Alpha-adducin (Erythrocyte adducin subunit alpha) |
| O75475 | PSIP1_HUMAN | PC4 and SFRS1-interacting protein (CLL-associated antigen KW-7) (Dense fine speckles 70 kDa protein) (DFS 70) (Lens epithelium-derived growth factor) (Transcriptional coactivator p75/p52) |
| P16949 | STMN1_HUMAN | Stathmin (Leukemia-associated phosphoprotein p18) (Metablastin) (Oncoprotein 18) (Op18) (Phosphoprotein p19) (pp19) (Prosolin) (Protein Pr22) (pp17) |
| Q9Y5B9 | SP16H_HUMAN | FACT complex subunit SPT16 (Chromatin-specific transcription elongation factor 140 kDa subunit) (FACT 140 kDa subunit) (FACTp140) (Facilitates chromatin transcription complex subunit SPT16) (hSPT16) |
| Q9NQ66 | PLCB1_HUMAN | 1-phosphatidylinositol 4,5-bisphosphate phosphodiesterase beta-1 (EC 3.1.4.11) (PLC-154) (Phosphoinositide phospholipase C-beta-1) (Phospholipase C-I) (PLC-I) (Phospholipase C-beta-1) (PLC-beta-1) |
| P52272 | HNRPM_HUMAN | Heterogeneous nuclear ribonucleoprotein M (hnRNP M) |
