# Supplementary material for: Proteomic analysis of the human retina reveals region-specific susceptibilities to metabolic- and oxidative stress-related diseases
Source: PLoS One. 2018 Feb 21;13(2):e0193250. doi: 10.1371/journal.pone.0193250 (PMC5821407; doi:10.1371/journal.pone.0193250)
Supplement: S5 Table — (DOCX) [file pone.0193250.s016.docx]

**Supplemental Table 5. Differentially-expressed proteins in the peripheral retina.**

| **UniProt ID** | **Entry name** | **Protein names** |
| --- | --- | --- |
| A0A024R216 | A0A024R216_HUMAN | Hepatoma-derived growth factor, related protein 3, isoform CRA_a (Hepatoma-derived growth factor-related protein 3) |
| O94973 | AP2A2_HUMAN | AP-2 complex subunit alpha-2 (100 kDa coated vesicle protein C) (Adaptor protein complex AP-2 subunit alpha-2) (Adaptor-related protein complex 2 subunit alpha-2) (Alpha-adaptin C) (Alpha2-adaptin) (Clathrin assembly protein complex 2 alpha-C large chain) (Huntingtin yeast partner J) (Huntingtin-interacting protein 9) (HIP-9) (Huntingtin-interacting protein J) (Plasma membrane adaptor HA2/AP2 adaptin alpha C subunit) |
| Q9HCD5 | NCOA5_HUMAN | Nuclear receptor coactivator 5 (NCoA-5) (Coactivator independent of AF-2) (CIA) |
| O76070 | SYUG_HUMAN | Gamma-synuclein (Breast cancer-specific gene 1 protein) (Persyn) (Synoretin) (SR) |
| Q9P2J5 | SYLC_HUMAN | Leucine--tRNA ligase, cytoplasmic (EC 6.1.1.4) (Leucyl-tRNA synthetase) (LeuRS) |
| Q13509 | TBB3_HUMAN | Tubulin beta-3 chain (Tubulin beta-4 chain) (Tubulin beta-III) |
| P04350 | TBB4A_HUMAN | Tubulin beta-4A chain (Tubulin 5 beta) (Tubulin beta-4 chain) |
| P68371 | TBB4B_HUMAN | Tubulin beta-4B chain (Tubulin beta-2 chain) (Tubulin beta-2C chain) |
| Q9BVA1 | TBB2B_HUMAN | Tubulin beta-2B chain |
| Q13885 | TBB2A_HUMAN | Tubulin beta-2A chain (Tubulin beta class IIa) |
| B7Z4B8 | B7Z4B8_HUMAN | Heterogeneous nuclear ribonucleoprotein U-like protein 1 (cDNA FLJ56481, highly similar to Heterogeneous nuclear ribonucleoprotein U-like protein 1) |
| Q16555 | DPYL2_HUMAN | Dihydropyrimidinase-related protein 2 (DRP-2) (Collapsin response mediator protein 2) (CRMP-2) (N2A3) (Unc-33-like phosphoprotein 2) (ULIP-2) |
| P60842 | IF4A1_HUMAN | Eukaryotic initiation factor 4A-I (eIF-4A-I) (eIF4A-I) (EC 3.6.4.13) (ATP-dependent RNA helicase eIF4A-1) |
| Q9H0D6 | XRN2_HUMAN | 5'-3' exoribonuclease 2 (EC 3.1.13.-) (DHM1-like protein) (DHP protein) |
| Q8IVF2 | AHNK2_HUMAN | Protein AHNAK2 |
| O95140 | MFN2_HUMAN | Mitofusin-2 (EC 3.6.5.-) (Transmembrane GTPase MFN2) |
| P29992 | GNA11_HUMAN | Guanine nucleotide-binding protein subunit alpha-11 (G alpha-11) (G-protein subunit alpha-11) (Guanine nucleotide-binding protein G(y) subunit alpha) |
| Q9BY11 | PACN1_HUMAN | Protein kinase C and casein kinase substrate in neurons protein 1 (Syndapin-1) |
| Q86Y39 | NDUAB_HUMAN | NADH dehydrogenase [ubiquinone] 1 alpha subcomplex subunit 11 (Complex I-B14.7) (CI-B14.7) (NADH-ubiquinone oxidoreductase subunit B14.7) |
| P62847 | RS24_HUMAN | 40S ribosomal protein S24 (Small ribosomal subunit protein eS24) |
| Q8IXT5 | RB12B_HUMAN | RNA-binding protein 12B (RNA-binding motif protein 12B) |
| P30038 | AL4A1_HUMAN | Delta-1-pyrroline-5-carboxylate dehydrogenase, mitochondrial (P5C dehydrogenase) (EC 1.2.1.88) (Aldehyde dehydrogenase family 4 member A1) (L-glutamate gamma-semialdehyde dehydrogenase) |
| A0A024R4E5 | A0A024R4E5_HUMAN | High density lipoprotein binding protein (Vigilin), isoform CRA_a (Vigilin) |
| D6RBK0 | D6RBK0_HUMAN | Prohibitin (Fragment) |
| Q9H2X9 | S12A5_HUMAN | Solute carrier family 12 member 5 (Electroneutral potassium-chloride cotransporter 2) (K-Cl cotransporter 2) (hKCC2) (Neuronal K-Cl cotransporter) |
| Q9UI15 | TAGL3_HUMAN | Transgelin-3 (Neuronal protein 22) (NP22) (Neuronal protein NP25) |
| P11177 | ODPB_HUMAN | Pyruvate dehydrogenase E1 component subunit beta, mitochondrial (PDHE1-B) (EC 1.2.4.1) |
| P46779 | RL28_HUMAN | 60S ribosomal protein L28 (Large ribosomal subunit protein eL28) |
| O95674 | CDS2_HUMAN | Phosphatidate cytidylyltransferase 2 (EC 2.7.7.41) (CDP-DAG synthase 2) (CDP-DG synthase 2) (CDP-diacylglycerol synthase 2) (CDS 2) (CDP-diglyceride pyrophosphorylase 2) (CDP-diglyceride synthase 2) (CTP:phosphatidate cytidylyltransferase 2) |
| O43809 | CPSF5_HUMAN | Cleavage and polyadenylation specificity factor subunit 5 (Cleavage and polyadenylation specificity factor 25 kDa subunit) (CFIm25) (CPSF 25 kDa subunit) (Nucleoside diphosphate-linked moiety X motif 21) (Nudix motif 21) (Pre-mRNA cleavage factor Im 25 kDa subunit) |
| Q96GD0 | PLPP_HUMAN | Pyridoxal phosphate phosphatase (PLP phosphatase) (EC 3.1.3.3) (EC 3.1.3.74) (Chronophin) |
| Q9NY47 | CA2D2_HUMAN | Voltage-dependent calcium channel subunit alpha-2/delta-2 (Voltage-gated calcium channel subunit alpha-2/delta-2) [Cleaved into: Voltage-dependent calcium channel subunit alpha-2-2; Voltage-dependent calcium channel subunit delta-2] |
| Q9UM22 | EPDR1_HUMAN | Mammalian ependymin-related protein 1 (MERP-1) (Upregulated in colorectal cancer gene 1 protein) |
| P36575 | ARRC_HUMAN | Arrestin-C (Cone arrestin) (C-arrestin) (cArr) (Retinal cone arrestin-3) (X-arrestin) |
| Q8TBC4 | UBA3_HUMAN | NEDD8-activating enzyme E1 catalytic subunit (EC 6.3.2.-) (NEDD8-activating enzyme E1C) (Ubiquitin-activating enzyme E1C) (Ubiquitin-like modifier-activating enzyme 3) (Ubiquitin-activating enzyme 3) |
| O95373 | IPO7_HUMAN | Importin-7 (Imp7) (Ran-binding protein 7) (RanBP7) |
| Q66K74 | MAP1S_HUMAN | Microtubule-associated protein 1S (MAP-1S) (BPY2-interacting protein 1) (Microtubule-associated protein 8) (Variable charge Y chromosome 2-interacting protein 1) (VCY2-interacting protein 1) (VCY2IP-1) [Cleaved into: MAP1S heavy chain; MAP1S light chain] |
| P16520 | GBB3_HUMAN | Guanine nucleotide-binding protein G(I)/G(S)/G(T) subunit beta-3 (Transducin beta chain 3) |
| P04216 | THY1_HUMAN | Thy-1 membrane glycoprotein (CDw90) (Thy-1 antigen) (CD antigen CD90) |
| A0A0A0MRA8 | A0A0A0MRA8_HUMAN | Band 4.1-like protein 3 |
| A0A0A0MSA4 | A0A0A0MSA4_HUMAN | Band 4.1-like protein 3 |
| P08247 | SYPH_HUMAN | Synaptophysin (Major synaptic vesicle protein p38) |
| Q14318 | FKBP8_HUMAN | Peptidyl-prolyl cis-trans isomerase FKBP8 (PPIase FKBP8) (EC 5.2.1.8) (38 kDa FK506-binding protein) (38 kDa FKBP) (FKBP-38) (hFKBP38) (FK506-binding protein 8) (FKBP-8) (FKBPR38) (Rotamase) |
| P07305 | H10_HUMAN | Histone H1.0 (Histone H1') (Histone H1(0)) [Cleaved into: Histone H1.0, N-terminally processed] |
| Q9BXK5 | B2L13_HUMAN | Bcl-2-like protein 13 (Bcl2-L-13) (Bcl-rambo) (Protein Mil1) |
| Q14008 | CKAP5_HUMAN | Cytoskeleton-associated protein 5 (Colonic and hepatic tumor overexpressed gene protein) (Ch-TOG) |
| Q13131 | AAPK1_HUMAN | 5'-AMP-activated protein kinase catalytic subunit alpha-1 (AMPK subunit alpha-1) (EC 2.7.11.1) (Acetyl-CoA carboxylase kinase) (ACACA kinase) (EC 2.7.11.27) (Hydroxymethylglutaryl-CoA reductase kinase) (HMGCR kinase) (EC 2.7.11.31) (Tau-protein kinase PRKAA1) (EC 2.7.11.26) |
| Q2M2I8 | AAK1_HUMAN | AP2-associated protein kinase 1 (EC 2.7.11.1) (Adaptor-associated kinase 1) |
| O60506 | HNRPQ_HUMAN | Heterogeneous nuclear ribonucleoprotein Q (hnRNP Q) (Glycine- and tyrosine-rich RNA-binding protein) (GRY-RBP) (NS1-associated protein 1) (Synaptotagmin-binding, cytoplasmic RNA-interacting protein) |
| P54687 | BCAT1_HUMAN | Branched-chain-amino-acid aminotransferase, cytosolic (BCAT(c)) (EC 2.6.1.42) (Protein ECA39) |
| P61158 | ARP3_HUMAN | Actin-related protein 3 (Actin-like protein 3) |
| P04181 | OAT_HUMAN | Ornithine aminotransferase, mitochondrial (EC 2.6.1.13) (Ornithine delta-aminotransferase) (Ornithine--oxo-acid aminotransferase) [Cleaved into: Ornithine aminotransferase, hepatic form; Ornithine aminotransferase, renal form] |
| O00159 | MYO1C_HUMAN | Unconventional myosin-Ic (Myosin I beta) (MMI-beta) (MMIb) |
| P36542 | ATPG_HUMAN | ATP synthase subunit gamma, mitochondrial (F-ATPase gamma subunit) |
| Q15386 | UBE3C_HUMAN | Ubiquitin-protein ligase E3C (EC 2.3.2.26) (HECT-type ubiquitin transferase E3C) (HectH2) |
| Q9ULD0 | OGDHL_HUMAN | 2-oxoglutarate dehydrogenase-like, mitochondrial (EC 1.2.4.-) (2-oxoglutarate dehydrogenase complex component E1-like) (OGDC-E1-like) (Alpha-ketoglutarate dehydrogenase-like) |
| P21796 | VDAC1_HUMAN | Voltage-dependent anion-selective channel protein 1 (VDAC-1) (hVDAC1) (Outer mitochondrial membrane protein porin 1) (Plasmalemmal porin) (Porin 31HL) (Porin 31HM) |
| J3KPX7 | J3KPX7_HUMAN | Prohibitin-2 |
| P56134 | ATPK_HUMAN | ATP synthase subunit f, mitochondrial |
| P48681 | NEST_HUMAN | Nestin |
| P62314 | SMD1_HUMAN | Small nuclear ribonucleoprotein Sm D1 (Sm-D1) (Sm-D autoantigen) (snRNP core protein D1) |
| Q5VV89 | Q5VV89_HUMAN | Microsomal glutathione S-transferase 3 |
| Q00610 | CLH1_HUMAN | Clathrin heavy chain 1 (Clathrin heavy chain on chromosome 17) (CLH-17) |
| P00403 | COX2_HUMAN | Cytochrome c oxidase subunit 2 (Cytochrome c oxidase polypeptide II) |
| Q9UJS0 | CMC2_HUMAN | Calcium-binding mitochondrial carrier protein Aralar2 (Citrin) (Mitochondrial aspartate glutamate carrier 2) (Solute carrier family 25 member 13) |
| C9JD32 | C9JD32_HUMAN | 60S ribosomal protein L23 (Fragment) |
| P08195 | 4F2_HUMAN | 4F2 cell-surface antigen heavy chain (4F2hc) (4F2 heavy chain antigen) (Lymphocyte activation antigen 4F2 large subunit) (Solute carrier family 3 member 2) (CD antigen CD98) |
| P10515 | ODP2_HUMAN | Dihydrolipoyllysine-residue acetyltransferase component of pyruvate dehydrogenase complex, mitochondrial (EC 2.3.1.12) (70 kDa mitochondrial autoantigen of primary biliary cirrhosis) (PBC) (Dihydrolipoamide acetyltransferase component of pyruvate dehydrogenase complex) (M2 antigen complex 70 kDa subunit) (Pyruvate dehydrogenase complex component E2) (PDC-E2) (PDCE2) |
| Q02978 | M2OM_HUMAN | Mitochondrial 2-oxoglutarate/malate carrier protein (OGCP) (Solute carrier family 25 member 11) |
| O75489 | NDUS3_HUMAN | NADH dehydrogenase [ubiquinone] iron-sulfur protein 3, mitochondrial (EC 1.6.5.3) (EC 1.6.99.3) (Complex I-30kD) (CI-30kD) (NADH-ubiquinone oxidoreductase 30 kDa subunit) |
| P19367 | HXK1_HUMAN | Hexokinase-1 (EC 2.7.1.1) (Brain form hexokinase) (Hexokinase type I) (HK I) |
| Q9UQ03 | COR2B_HUMAN | Coronin-2B (Coronin-like protein C) (Clipin-C) (Protein FC96) |
| Q92990 | GLMN_HUMAN | Glomulin (FK506-binding protein-associated protein) (FAP) (FKBP-associated protein) |
| Q9NZN4 | EHD2_HUMAN | EH domain-containing protein 2 (PAST homolog 2) |
| Q7Z6Z7 | HUWE1_HUMAN | E3 ubiquitin-protein ligase HUWE1 (EC 2.3.2.26) (ARF-binding protein 1) (ARF-BP1) (HECT, UBA and WWE domain-containing protein 1) (HECT-type E3 ubiquitin transferase HUWE1) (Homologous to E6AP carboxyl terminus homologous protein 9) (HectH9) (Large structure of UREB1) (LASU1) (Mcl-1 ubiquitin ligase E3) (Mule) (Upstream regulatory element-binding protein 1) (URE-B1) (URE-binding protein 1) |
| Q8TDJ6 | DMXL2_HUMAN | DmX-like protein 2 (Rabconnectin-3) |
| Q9NXG2 | THUM1_HUMAN | THUMP domain-containing protein 1 |
| Q07021 | C1QBP_HUMAN | Complement component 1 Q subcomponent-binding protein, mitochondrial (ASF/SF2-associated protein p32) (Glycoprotein gC1qBP) (C1qBP) (Hyaluronan-binding protein 1) (Mitochondrial matrix protein p32) (gC1q-R protein) (p33) |
| H9KV28 | H9KV28_HUMAN | Protein diaphanous homolog 1 |
| P05388 | RLA0_HUMAN | 60S acidic ribosomal protein P0 (60S ribosomal protein L10E) (Large ribosomal subunit protein uL10) |
| P83731 | RL24_HUMAN | 60S ribosomal protein L24 (60S ribosomal protein L30) (Large ribosomal subunit protein eL24) |
| O43681 | ASNA_HUMAN | ATPase ASNA1 (EC 3.6.-.-) (Arsenical pump-driving ATPase) (Arsenite-stimulated ATPase) (Transmembrane domain recognition complex 40 kDa ATPase subunit) (hARSA-I) (hASNA-I) |
| Q9UI12 | VATH_HUMAN | V-type proton ATPase subunit H (V-ATPase subunit H) (Nef-binding protein 1) (NBP1) (Protein VMA13 homolog) (V-ATPase 50/57 kDa subunits) (Vacuolar proton pump subunit H) (Vacuolar proton pump subunit SFD) |
| Q9NS69 | TOM22_HUMAN | Mitochondrial import receptor subunit TOM22 homolog (hTom22) (1C9-2) (Translocase of outer membrane 22 kDa subunit homolog) |
| P61247 | RS3A_HUMAN | 40S ribosomal protein S3a (Small ribosomal subunit protein eS1) (v-fos transformation effector protein) (Fte-1) |
| P49748 | ACADV_HUMAN | Very long-chain specific acyl-CoA dehydrogenase, mitochondrial (VLCAD) (EC 1.3.8.9) |
| O75390 | CISY_HUMAN | Citrate synthase, mitochondrial (EC 2.3.3.1) (Citrate (Si)-synthase) |
| Q9UBB6 | NCDN_HUMAN | Neurochondrin |
| Q7Z460 | CLAP1_HUMAN | CLIP-associating protein 1 (Cytoplasmic linker-associated protein 1) (Multiple asters homolog 1) (Protein Orbit homolog 1) (hOrbit1) |
| P08238 | HS90B_HUMAN | Heat shock protein HSP 90-beta (HSP 90) (Heat shock 84 kDa) (HSP 84) (HSP84) |
| Q92896 | GSLG1_HUMAN | Golgi apparatus protein 1 (CFR-1) (Cysteine-rich fibroblast growth factor receptor) (E-selectin ligand 1) (ESL-1) (Golgi sialoglycoprotein MG-160) |
| O43148 | MCES_HUMAN | mRNA cap guanine-N7 methyltransferase (EC 2.1.1.56) (RG7MT1) (mRNA (guanine-N(7)-)-methyltransferase) (mRNA cap methyltransferase) (hCMT1) (hMet) (hcm1p) |
| Q8TBG9 | SYNPR_HUMAN | Synaptoporin |
| Q9NSD9 | SYFB_HUMAN | Phenylalanine--tRNA ligase beta subunit (EC 6.1.1.20) (Phenylalanyl-tRNA synthetase beta subunit) (PheRS) |
| P62760 | VISL1_HUMAN | Visinin-like protein 1 (VILIP) (VLP-1) (Hippocalcin-like protein 3) (HLP3) |
| P53367 | ARFP1_HUMAN | Arfaptin-1 (ADP-ribosylation factor-interacting protein 1) |
| Q15067 | ACOX1_HUMAN | Peroxisomal acyl-coenzyme A oxidase 1 (AOX) (EC 1.3.3.6) (Palmitoyl-CoA oxidase) (Straight-chain acyl-CoA oxidase) (SCOX) |
| P54709 | AT1B3_HUMAN | Sodium/potassium-transporting ATPase subunit beta-3 (Sodium/potassium-dependent ATPase subunit beta-3) (ATPB-3) (CD antigen CD298) |
| Q13155 | AIMP2_HUMAN | Aminoacyl tRNA synthase complex-interacting multifunctional protein 2 (Multisynthase complex auxiliary component p38) (Protein JTV-1) |
| Q6IBS0 | TWF2_HUMAN | Twinfilin-2 (A6-related protein) (hA6RP) (Protein tyrosine kinase 9-like) (Twinfilin-1-like protein) |
| Q96QR8 | PURB_HUMAN | Transcriptional activator protein Pur-beta (Purine-rich element-binding protein B) |
| P23396 | RS3_HUMAN | 40S ribosomal protein S3 (EC 4.2.99.18) (Small ribosomal subunit protein uS3) |
| O75964 | ATP5L_HUMAN | ATP synthase subunit g, mitochondrial (ATPase subunit g) |
| P59998 | ARPC4_HUMAN | Actin-related protein 2/3 complex subunit 4 (Arp2/3 complex 20 kDa subunit) (p20-ARC) |
| O00154 | BACH_HUMAN | Cytosolic acyl coenzyme A thioester hydrolase (EC 3.1.2.2) (Acyl-CoA thioesterase 7) (Brain acyl-CoA hydrolase) (BACH) (CTE-IIa) (CTE-II) (Long chain acyl-CoA thioester hydrolase) |
| J3KN01 | J3KN01_HUMAN | Afadin |
| P05198 | IF2A_HUMAN | Eukaryotic translation initiation factor 2 subunit 1 (Eukaryotic translation initiation factor 2 subunit alpha) (eIF-2-alpha) (eIF-2A) (eIF-2alpha) |
| P62330 | ARF6_HUMAN | ADP-ribosylation factor 6 |
| Q9H0B6 | KLC2_HUMAN | Kinesin light chain 2 (KLC 2) |
| P61313 | RL15_HUMAN | 60S ribosomal protein L15 (Large ribosomal subunit protein eL15) |
| Q14576 | ELAV3_HUMAN | ELAV-like protein 3 (Hu-antigen C) (HuC) (Paraneoplastic cerebellar degeneration-associated antigen) (Paraneoplastic limbic encephalitis antigen 21) |
| O43617 | TPPC3_HUMAN | Trafficking protein particle complex subunit 3 (BET3 homolog) |
| O94925 | GLSK_HUMAN | Glutaminase kidney isoform, mitochondrial (GLS) (EC 3.5.1.2) (K-glutaminase) (L-glutamine amidohydrolase) |
| P30049 | ATPD_HUMAN | ATP synthase subunit delta, mitochondrial (F-ATPase delta subunit) |
| Q15813 | TBCE_HUMAN | Tubulin-specific chaperone E (Tubulin-folding cofactor E) |
| P41252 | SYIC_HUMAN | Isoleucine--tRNA ligase, cytoplasmic (EC 6.1.1.5) (Isoleucyl-tRNA synthetase) (IRS) (IleRS) |
| P61204 | ARF3_HUMAN | ADP-ribosylation factor 3 |
| O00468 | AGRIN_HUMAN | Agrin [Cleaved into: Agrin N-terminal 110 kDa subunit; Agrin C-terminal 110 kDa subunit; Agrin C-terminal 90 kDa fragment (C90); Agrin C-terminal 22 kDa fragment (C22)] |
| O75122 | CLAP2_HUMAN | CLIP-associating protein 2 (Cytoplasmic linker-associated protein 2) (Protein Orbit homolog 2) (hOrbit2) |
| Q14204 | DYHC1_HUMAN | Cytoplasmic dynein 1 heavy chain 1 (Cytoplasmic dynein heavy chain 1) (Dynein heavy chain, cytosolic) |
| O95782 | AP2A1_HUMAN | AP-2 complex subunit alpha-1 (100 kDa coated vesicle protein A) (Adaptor protein complex AP-2 subunit alpha-1) (Adaptor-related protein complex 2 subunit alpha-1) (Alpha-adaptin A) (Alpha1-adaptin) (Clathrin assembly protein complex 2 alpha-A large chain) (Plasma membrane adaptor HA2/AP2 adaptin alpha A subunit) |
| Q9UDW1 | QCR9_HUMAN | Cytochrome b-c1 complex subunit 9 (Complex III subunit 9) (Complex III subunit X) (Cytochrome c1 non-heme 7 kDa protein) (Ubiquinol-cytochrome c reductase complex 7.2 kDa protein) |
| P55010 | IF5_HUMAN | Eukaryotic translation initiation factor 5 (eIF-5) |
| Q53GS9 | SNUT2_HUMAN | U4/U6.U5 tri-snRNP-associated protein 2 (Inactive ubiquitin-specific peptidase 39) (SAD1 homolog) (U4/U6.U5 tri-snRNP-associated 65 kDa protein) (65K) |
| P28074 | PSB5_HUMAN | Proteasome subunit beta type-5 (EC 3.4.25.1) (Macropain epsilon chain) (Multicatalytic endopeptidase complex epsilon chain) (Proteasome chain 6) (Proteasome epsilon chain) (Proteasome subunit MB1) (Proteasome subunit X) |
| Q01814 | AT2B2_HUMAN | Plasma membrane calcium-transporting ATPase 2 (PMCA2) (EC 3.6.3.8) (Plasma membrane calcium ATPase isoform 2) (Plasma membrane calcium pump isoform 2) |
| Q9BSJ8 | ESYT1_HUMAN | Extended synaptotagmin-1 (E-Syt1) (Membrane-bound C2 domain-containing protein) |
| P36915 | GNL1_HUMAN | Guanine nucleotide-binding protein-like 1 (GTP-binding protein HSR1) |
| P26639 | SYTC_HUMAN | Threonine--tRNA ligase, cytoplasmic (EC 6.1.1.3) (Threonyl-tRNA synthetase) (ThrRS) |
| Q13813 | SPTN1_HUMAN | Spectrin alpha chain, non-erythrocytic 1 (Alpha-II spectrin) (Fodrin alpha chain) (Spectrin, non-erythroid alpha subunit) |
| P50914 | RL14_HUMAN | 60S ribosomal protein L14 (CAG-ISL 7) (Large ribosomal subunit protein eL14) |
| Q9BWD1 | THIC_HUMAN | Acetyl-CoA acetyltransferase, cytosolic (EC 2.3.1.9) (Acetyl-CoA transferase-like protein) (Cytosolic acetoacetyl-CoA thiolase) |
| E9PGC8 | E9PGC8_HUMAN | Microtubule-associated protein 1A |
| P61106 | RAB14_HUMAN | Ras-related protein Rab-14 |
| Q9P0L0 | VAPA_HUMAN | Vesicle-associated membrane protein-associated protein A (VAMP-A) (VAMP-associated protein A) (VAP-A) (33 kDa VAMP-associated protein) (VAP-33) |
| Q9P2U7 | VGLU1_HUMAN | Vesicular glutamate transporter 1 (VGluT1) (Brain-specific Na(+)-dependent inorganic phosphate cotransporter) (Solute carrier family 17 member 7) |
| P51160 | PDE6C_HUMAN | Cone cGMP-specific 3',5'-cyclic phosphodiesterase subunit alpha' (EC 3.1.4.35) (cGMP phosphodiesterase 6C) |
| P20020 | AT2B1_HUMAN | Plasma membrane calcium-transporting ATPase 1 (PMCA1) (EC 3.6.3.8) (Plasma membrane calcium ATPase isoform 1) (Plasma membrane calcium pump isoform 1) |
| Q9Y639 | NPTN_HUMAN | Neuroplastin (Stromal cell-derived receptor 1) (SDR-1) |
| Q06278 | AOXA_HUMAN | Aldehyde oxidase (EC 1.2.3.1) (Aldehyde oxidase 1) (Azaheterocycle hydroxylase) (EC 1.17.3.-) |
| O95433 | AHSA1_HUMAN | Activator of 90 kDa heat shock protein ATPase homolog 1 (AHA1) (p38) |
| P20336 | RAB3A_HUMAN | Ras-related protein Rab-3A |
| O43301 | HS12A_HUMAN | Heat shock 70 kDa protein 12A |
| O94906 | PRP6_HUMAN | Pre-mRNA-processing factor 6 (Androgen receptor N-terminal domain-transactivating protein 1) (ANT-1) (PRP6 homolog) (U5 snRNP-associated 102 kDa protein) (U5-102 kDa protein) |
| P04275 | VWF_HUMAN | von Willebrand factor (vWF) [Cleaved into: von Willebrand antigen 2 (von Willebrand antigen II)] |
| Q8N573 | OXR1_HUMAN | Oxidation resistance protein 1 |
| O60282 | KIF5C_HUMAN | Kinesin heavy chain isoform 5C (Kinesin heavy chain neuron-specific 2) |
| J3KNA1 | J3KNA1_HUMAN | Kinesin-like protein |
| P53677 | AP3M2_HUMAN | AP-3 complex subunit mu-2 (Adaptor-related protein complex 3 subunit mu-2) (Clathrin assembly protein assembly protein complex 3 mu-2 medium chain) (Clathrin coat assembly protein AP47 homolog 2) (Clathrin coat-associated protein AP47 homolog 2) (Golgi adaptor AP-1 47 kDa protein homolog 2) (HA1 47 kDa subunit homolog 2) (Mu3B-adaptin) (P47B) |
| P35268 | RL22_HUMAN | 60S ribosomal protein L22 (EBER-associated protein) (EAP) (Epstein-Barr virus small RNA-associated protein) (Heparin-binding protein HBp15) (Large ribosomal subunit protein eL22) |
| P46821 | MAP1B_HUMAN | Microtubule-associated protein 1B (MAP-1B) [Cleaved into: MAP1B heavy chain; MAP1 light chain LC1] |
| Q9UBC2 | EP15R_HUMAN | Epidermal growth factor receptor substrate 15-like 1 (Eps15-related protein) (Eps15R) |
| P46379 | BAG6_HUMAN | Large proline-rich protein BAG6 (BAG family molecular chaperone regulator 6) (BCL2-associated athanogene 6) (BAG-6) (HLA-B-associated transcript 3) (Protein G3) (Protein Scythe) |
| P21579 | SYT1_HUMAN | Synaptotagmin-1 (Synaptotagmin I) (SytI) (p65) |
| P05023 | AT1A1_HUMAN | Sodium/potassium-transporting ATPase subunit alpha-1 (Na(+)/K(+) ATPase alpha-1 subunit) (EC 3.6.3.9) (Sodium pump subunit alpha-1) |
| P31153 | METK2_HUMAN | S-adenosylmethionine synthase isoform type-2 (AdoMet synthase 2) (EC 2.5.1.6) (Methionine adenosyltransferase 2) (MAT 2) (Methionine adenosyltransferase II) (MAT-II) |
| Q02790 | FKBP4_HUMAN | Peptidyl-prolyl cis-trans isomerase FKBP4 (PPIase FKBP4) (EC 5.2.1.8) (51 kDa FK506-binding protein) (FKBP51) (52 kDa FK506-binding protein) (52 kDa FKBP) (FKBP-52) (59 kDa immunophilin) (p59) (FK506-binding protein 4) (FKBP-4) (FKBP59) (HSP-binding immunophilin) (HBI) (Immunophilin FKBP52) (Rotamase) [Cleaved into: Peptidyl-prolyl cis-trans isomerase FKBP4, N-terminally processed] |
| P49840 | GSK3A_HUMAN | Glycogen synthase kinase-3 alpha (GSK-3 alpha) (EC 2.7.11.26) (Serine/threonine-protein kinase GSK3A) (EC 2.7.11.1) |
| Q9BUF5 | TBB6_HUMAN | Tubulin beta-6 chain (Tubulin beta class V) |
| Q01082 | SPTB2_HUMAN | Spectrin beta chain, non-erythrocytic 1 (Beta-II spectrin) (Fodrin beta chain) (Spectrin, non-erythroid beta chain 1) |
| P51674 | GPM6A_HUMAN | Neuronal membrane glycoprotein M6-a (M6a) |
| O00519 | FAAH1_HUMAN | Fatty-acid amide hydrolase 1 (EC 3.5.1.99) (Anandamide amidohydrolase 1) (Oleamide hydrolase 1) |
| Q12904 | AIMP1_HUMAN | Aminoacyl tRNA synthase complex-interacting multifunctional protein 1 (Multisynthase complex auxiliary component p43) [Cleaved into: Endothelial monocyte-activating polypeptide 2 (EMAP-2) (Endothelial monocyte-activating polypeptide II) (EMAP-II) (Small inducible cytokine subfamily E member 1)] |
| Q9Y3D6 | FIS1_HUMAN | Mitochondrial fission 1 protein (FIS1 homolog) (hFis1) (Tetratricopeptide repeat protein 11) (TPR repeat protein 11) |
| P61764 | STXB1_HUMAN | Syntaxin-binding protein 1 (MUNC18-1) (N-Sec1) (Protein unc-18 homolog 1) (Unc18-1) (Protein unc-18 homolog A) (Unc-18A) (p67) |
| P48449 | ERG7_HUMAN | Lanosterol synthase (EC 5.4.99.7) (2,3-epoxysqualene--lanosterol cyclase) (Oxidosqualene--lanosterol cyclase) (OSC) (hOSC) |
| P07814 | SYEP_HUMAN | Bifunctional glutamate/proline--tRNA ligase (Bifunctional aminoacyl-tRNA synthetase) (Cell proliferation-inducing gene 32 protein) (Glutamatyl-prolyl-tRNA synthetase) [Includes: Glutamate--tRNA ligase (EC 6.1.1.17) (Glutamyl-tRNA synthetase) (GluRS); Proline--tRNA ligase (EC 6.1.1.15) (Prolyl-tRNA synthetase)] |
| Q8WUK0 | PTPM1_HUMAN | Phosphatidylglycerophosphatase and protein-tyrosine phosphatase 1 (EC 3.1.3.27) (PTEN-like phosphatase) (Phosphoinositide lipid phosphatase) (Protein-tyrosine phosphatase mitochondrial 1) (EC 3.1.3.16) (EC 3.1.3.48) |
| Q13595 | TRA2A_HUMAN | Transformer-2 protein homolog alpha (TRA-2 alpha) (TRA2-alpha) (Transformer-2 protein homolog A) |
| Q86X55 | CARM1_HUMAN | Histone-arginine methyltransferase CARM1 (EC 2.1.1.319) (Coactivator-associated arginine methyltransferase 1) (Protein arginine N-methyltransferase 4) |
| Q15555 | MARE2_HUMAN | Microtubule-associated protein RP/EB family member 2 (APC-binding protein EB2) (End-binding protein 2) (EB2) |
| P12036 | NFH_HUMAN | Neurofilament heavy polypeptide (NF-H) (200 kDa neurofilament protein) (Neurofilament triplet H protein) |
| P07954 | FUMH_HUMAN | Fumarate hydratase, mitochondrial (Fumarase) (EC 4.2.1.2) |
| Q13423 | NNTM_HUMAN | NAD(P) transhydrogenase, mitochondrial (EC 1.6.1.2) (Nicotinamide nucleotide transhydrogenase) (Pyridine nucleotide transhydrogenase) |
| Q15436 | SC23A_HUMAN | Protein transport protein Sec23A (SEC23-related protein A) |
| Q9NY65 | TBA8_HUMAN | Tubulin alpha-8 chain (Alpha-tubulin 8) (Tubulin alpha chain-like 2) |
| O15540 | FABP7_HUMAN | Fatty acid-binding protein, brain (Brain lipid-binding protein) (BLBP) (Brain-type fatty acid-binding protein) (B-FABP) (Fatty acid-binding protein 7) (Mammary-derived growth inhibitor related) |
| X6R2P6 | X6R2P6_HUMAN | Programmed cell death protein 5 |
| Q15121 | PEA15_HUMAN | Astrocytic phosphoprotein PEA-15 (15 kDa phosphoprotein enriched in astrocytes) (Phosphoprotein enriched in diabetes) (PED) |
| O60721 | NCKX1_HUMAN | Sodium/potassium/calcium exchanger 1 (Na(+)/K(+)/Ca(2+)-exchange protein 1) (Retinal rod Na-Ca+K exchanger) (Solute carrier family 24 member 1) |
| O00264 | PGRC1_HUMAN | Membrane-associated progesterone receptor component 1 (mPR) |
| P61457 | PHS_HUMAN | Pterin-4-alpha-carbinolamine dehydratase (PHS) (EC 4.2.1.96) (4-alpha-hydroxy-tetrahydropterin dehydratase) (Dimerization cofactor of hepatocyte nuclear factor 1-alpha) (DCoH) (Dimerization cofactor of HNF1) (Phenylalanine hydroxylase-stimulating protein) (Pterin carbinolamine dehydratase) (PCD) |
| Q13045 | FLII_HUMAN | Protein flightless-1 homolog |
| Q9Y490 | TLN1_HUMAN | Talin-1 |
| O60293 | ZC3H1_HUMAN | Zinc finger C3H1 domain-containing protein (Coiled-coil domain-containing protein 131) (Proline/serine-rich coiled-coil protein 2) |
| Q5T9B7 | Q5T9B7_HUMAN | Adenylate kinase isoenzyme 1 (AK 1) (EC 2.7.4.3) (EC 2.7.4.6) (ATP-AMP transphosphorylase 1) (ATP:AMP phosphotransferase) (Adenylate monophosphate kinase) (Myokinase) |
| P08621 | RU17_HUMAN | U1 small nuclear ribonucleoprotein 70 kDa (U1 snRNP 70 kDa) (U1-70K) (snRNP70) |
| P0C0S5 | H2AZ_HUMAN | Histone H2A.Z (H2A/z) |
| P62310 | LSM3_HUMAN | U6 snRNA-associated Sm-like protein LSm3 |
| P52597 | HNRPF_HUMAN | Heterogeneous nuclear ribonucleoprotein F (hnRNP F) (Nucleolin-like protein mcs94-1) [Cleaved into: Heterogeneous nuclear ribonucleoprotein F, N-terminally processed] |
| P07108 | ACBP_HUMAN | Acyl-CoA-binding protein (ACBP) (Diazepam-binding inhibitor) (DBI) (Endozepine) (EP) |
| P45973 | CBX5_HUMAN | Chromobox protein homolog 5 (Antigen p25) (Heterochromatin protein 1 homolog alpha) (HP1 alpha) |
| P08754 | GNAI3_HUMAN | Guanine nucleotide-binding protein G(k) subunit alpha (G(i) alpha-3) |
| P04899 | GNAI2_HUMAN | Guanine nucleotide-binding protein G(i) subunit alpha-2 (Adenylate cyclase-inhibiting G alpha protein) |
| Q16864 | VATF_HUMAN | V-type proton ATPase subunit F (V-ATPase subunit F) (V-ATPase 14 kDa subunit) (Vacuolar proton pump subunit F) |
| P10599 | THIO_HUMAN | Thioredoxin (Trx) (ATL-derived factor) (ADF) (Surface-associated sulphydryl protein) (SASP) |
| P04792 | HSPB1_HUMAN | Heat shock protein beta-1 (HspB1) (28 kDa heat shock protein) (Estrogen-regulated 24 kDa protein) (Heat shock 27 kDa protein) (HSP 27) (Stress-responsive protein 27) (SRP27) |
| F8WE04 | F8WE04_HUMAN | Heat shock protein beta-1 |
| Q15631 | TSN_HUMAN | Translin (EC 3.1.-.-) (Component 3 of promoter of RISC) (C3PO) |
| P41219 | PERI_HUMAN | Peripherin (Neurofilament 4) |
| P02489 | CRYAA_HUMAN | Alpha-crystallin A chain (Heat shock protein beta-4) (HspB4) [Cleaved into: Alpha-crystallin A(1-172); Alpha-crystallin A(1-168); Alpha-crystallin A(1-162)] |
| Q9HC38 | GLOD4_HUMAN | Glyoxalase domain-containing protein 4 |
| P16070 | CD44_HUMAN | CD44 antigen (CDw44) (Epican) (Extracellular matrix receptor III) (ECMR-III) (GP90 lymphocyte homing/adhesion receptor) (HUTCH-I) (Heparan sulfate proteoglycan) (Hermes antigen) (Hyaluronate receptor) (Phagocytic glycoprotein 1) (PGP-1) (Phagocytic glycoprotein I) (PGP-I) (CD antigen CD44) |
| P08138 | TNR16_HUMAN | Tumor necrosis factor receptor superfamily member 16 (Gp80-LNGFR) (Low affinity neurotrophin receptor p75NTR) (Low-affinity nerve growth factor receptor) (NGF receptor) (p75 ICD) (CD antigen CD271) |
| P09382 | LEG1_HUMAN | Galectin-1 (Gal-1) (14 kDa laminin-binding protein) (HLBP14) (14 kDa lectin) (Beta-galactoside-binding lectin L-14-I) (Galaptin) (HBL) (HPL) (Lactose-binding lectin 1) (Lectin galactoside-binding soluble 1) (Putative MAPK-activating protein PM12) (S-Lac lectin 1) |
| Q14028 | CNGB1_HUMAN | Cyclic nucleotide-gated cation channel beta-1 (Cyclic nucleotide-gated cation channel 4) (CNG channel 4) (CNG-4) (CNG4) (Cyclic nucleotide-gated cation channel gamma) (Cyclic nucleotide-gated cation channel modulatory subunit) (Cyclic nucleotide-gated channel beta-1) (CNG channel beta-1) (Glutamic acid-rich protein) (GARP) |
| P30043 | BLVRB_HUMAN | Flavin reductase (NADPH) (FR) (EC 1.5.1.30) (Biliverdin reductase B) (BVR-B) (EC 1.3.1.24) (Biliverdin-IX beta-reductase) (Green heme-binding protein) (GHBP) (NADPH-dependent diaphorase) (NADPH-flavin reductase) (FLR) |
| O15173 | PGRC2_HUMAN | Membrane-associated progesterone receptor component 2 (Progesterone membrane-binding protein) (Steroid receptor protein DG6) |
| Q9NZL9 | MAT2B_HUMAN | Methionine adenosyltransferase 2 subunit beta (Methionine adenosyltransferase II beta) (MAT II beta) (Putative dTDP-4-keto-6-deoxy-D-glucose 4-reductase) |
| P21291 | CSRP1_HUMAN | Cysteine and glycine-rich protein 1 (Cysteine-rich protein 1) (CRP) (CRP1) (Epididymis luminal protein 141) (HEL-141) |
| O95336 | 6PGL_HUMAN | 6-phosphogluconolactonase (6PGL) (EC 3.1.1.31) |
| Q13435 | SF3B2_HUMAN | Splicing factor 3B subunit 2 (Pre-mRNA-splicing factor SF3b 145 kDa subunit) (SF3b145) (SF3b150) (Spliceosome-associated protein 145) (SAP 145) |
| P10768 | ESTD_HUMAN | S-formylglutathione hydrolase (FGH) (EC 3.1.2.12) (Esterase D) (Methylumbelliferyl-acetate deacetylase) (EC 3.1.1.56) |
| P24666 | PPAC_HUMAN | Low molecular weight phosphotyrosine protein phosphatase (LMW-PTP) (LMW-PTPase) (EC 3.1.3.48) (Adipocyte acid phosphatase) (Low molecular weight cytosolic acid phosphatase) (EC 3.1.3.2) (Red cell acid phosphatase 1) |
| P08670 | VIME_HUMAN | Vimentin |
| P08100 | OPSD_HUMAN | Rhodopsin (Opsin-2) |
| J3KNF4 | J3KNF4_HUMAN | Copper chaperone for superoxide dismutase |
| Q9Y281 | COF2_HUMAN | Cofilin-2 (Cofilin, muscle isoform) |
| P35222 | CTNB1_HUMAN | Catenin beta-1 (Beta-catenin) |
| O94760 | DDAH1_HUMAN | N(G),N(G)-dimethylarginine dimethylaminohydrolase 1 (DDAH-1) (Dimethylarginine dimethylaminohydrolase 1) (EC 3.5.3.18) (DDAHI) (Dimethylargininase-1) |
| P28482 | MK01_HUMAN | Mitogen-activated protein kinase 1 (MAP kinase 1) (MAPK 1) (EC 2.7.11.24) (ERT1) (Extracellular signal-regulated kinase 2) (ERK-2) (MAP kinase isoform p42) (p42-MAPK) (Mitogen-activated protein kinase 2) (MAP kinase 2) (MAPK 2) |
| P16949 | STMN1_HUMAN | Stathmin (Leukemia-associated phosphoprotein p18) (Metablastin) (Oncoprotein 18) (Op18) (Phosphoprotein p19) (pp19) (Prosolin) (Protein Pr22) (pp17) |
| Q6FI13 | H2A2A_HUMAN | Histone H2A type 2-A (Histone H2A.2) (Histone H2A/o) |
| P15104 | GLNA_HUMAN | Glutamine synthetase (GS) (EC 6.3.1.2) (Glutamate decarboxylase) (EC 4.1.1.15) (Glutamate--ammonia ligase) |
| P25789 | PSA4_HUMAN | Proteasome subunit alpha type-4 (EC 3.4.25.1) (Macropain subunit C9) (Multicatalytic endopeptidase complex subunit C9) (Proteasome component C9) (Proteasome subunit L) |
| P52272 | HNRPM_HUMAN | Heterogeneous nuclear ribonucleoprotein M (hnRNP M) |
| P08134 | RHOC_HUMAN | Rho-related GTP-binding protein RhoC (Rho cDNA clone 9) (h9) |
| P30086 | PEBP1_HUMAN | Phosphatidylethanolamine-binding protein 1 (PEBP-1) (HCNPpp) (Neuropolypeptide h3) (Prostatic-binding protein) (Raf kinase inhibitor protein) (RKIP) [Cleaved into: Hippocampal cholinergic neurostimulating peptide (HCNP)] |
| Q9P121 | NTRI_HUMAN | Neurotrimin (hNT) (IgLON family member 2) |
| Q9NQR4 | NIT2_HUMAN | Omega-amidase NIT2 (EC 3.5.1.3) (Nitrilase homolog 2) |
| O43488 | ARK72_HUMAN | Aflatoxin B1 aldehyde reductase member 2 (EC 1.1.1.n11) (AFB1 aldehyde reductase 1) (AFB1-AR 1) (Aldoketoreductase 7) (Succinic semialdehyde reductase) (SSA reductase) |
| Q92599 | SEPT8_HUMAN | Septin-8 |
| O75947 | ATP5H_HUMAN | ATP synthase subunit d, mitochondrial (ATPase subunit d) |
| P60174 | TPIS_HUMAN | Triosephosphate isomerase (TIM) (EC 5.3.1.1) (Triose-phosphate isomerase) |
| Q13642 | FHL1_HUMAN | Four and a half LIM domains protein 1 (FHL-1) (Skeletal muscle LIM-protein 1) (SLIM) (SLIM-1) |
| P07197 | NFM_HUMAN | Neurofilament medium polypeptide (NF-M) (160 kDa neurofilament protein) (Neurofilament 3) (Neurofilament triplet M protein) |
| Q06830 | PRDX1_HUMAN | Peroxiredoxin-1 (EC 1.11.1.15) (Natural killer cell-enhancing factor A) (NKEF-A) (Proliferation-associated gene protein) (PAG) (Thioredoxin peroxidase 2) (Thioredoxin-dependent peroxide reductase 2) |
| P17661 | DESM_HUMAN | Desmin |
| Q16352 | AINX_HUMAN | Alpha-internexin (Alpha-Inx) (66 kDa neurofilament protein) (NF-66) (Neurofilament-66) (Neurofilament 5) |
| P60900 | PSA6_HUMAN | Proteasome subunit alpha type-6 (EC 3.4.25.1) (27 kDa prosomal protein) (PROS-27) (p27K) (Macropain iota chain) (Multicatalytic endopeptidase complex iota chain) (Proteasome iota chain) |
| P32119 | PRDX2_HUMAN | Peroxiredoxin-2 (EC 1.11.1.15) (Natural killer cell-enhancing factor B) (NKEF-B) (PRP) (Thiol-specific antioxidant protein) (TSA) (Thioredoxin peroxidase 1) (Thioredoxin-dependent peroxide reductase 1) |
| P23528 | COF1_HUMAN | Cofilin-1 (18 kDa phosphoprotein) (p18) (Cofilin, non-muscle isoform) |
| P60880 | SNP25_HUMAN | Synaptosomal-associated protein 25 (SNAP-25) (Super protein) (SUP) (Synaptosomal-associated 25 kDa protein) |
| P0DMV9 | HS71B_HUMAN | Heat shock 70 kDa protein 1B (Heat shock 70 kDa protein 2) (HSP70-2) (HSP70.2) |
| P34931 | HS71L_HUMAN | Heat shock 70 kDa protein 1-like (Heat shock 70 kDa protein 1L) (Heat shock 70 kDa protein 1-Hom) (HSP70-Hom) |
| P30101 | PDIA3_HUMAN | Protein disulfide-isomerase A3 (EC 5.3.4.1) (58 kDa glucose-regulated protein) (58 kDa microsomal protein) (p58) (Disulfide isomerase ER-60) (Endoplasmic reticulum resident protein 57) (ER protein 57) (ERp57) (Endoplasmic reticulum resident protein 60) (ER protein 60) (ERp60) |
| Q5TZA2 | CROCC_HUMAN | Rootletin (Ciliary rootlet coiled-coil protein) |
| Q3ZCW2 | LEGL_HUMAN | Galectin-related protein (Lectin galactoside-binding-like protein) |
| B1AK87 | B1AK87_HUMAN | Capping protein (Actin filament) muscle Z-line, beta, isoform CRA_a (F-actin-capping protein subunit beta) |
| P62873 | GBB1_HUMAN | Guanine nucleotide-binding protein G(I)/G(S)/G(T) subunit beta-1 (Transducin beta chain 1) |
| P11488 | GNAT1_HUMAN | Guanine nucleotide-binding protein G(t) subunit alpha-1 (Transducin alpha-1 chain) |
| Q96AQ6 | PBIP1_HUMAN | Pre-B-cell leukemia transcription factor-interacting protein 1 (Hematopoietic PBX-interacting protein) |
| P14866 | HNRPL_HUMAN | Heterogeneous nuclear ribonucleoprotein L (hnRNP L) |
| P55809 | SCOT1_HUMAN | Succinyl-CoA:3-ketoacid coenzyme A transferase 1, mitochondrial (EC 2.8.3.5) (3-oxoacid CoA-transferase 1) (Somatic-type succinyl-CoA:3-oxoacid CoA-transferase) (SCOT-s) |
| P26599 | PTBP1_HUMAN | Polypyrimidine tract-binding protein 1 (PTB) (57 kDa RNA-binding protein PPTB-1) (Heterogeneous nuclear ribonucleoprotein I) (hnRNP I) |
| P43487 | RANG_HUMAN | Ran-specific GTPase-activating protein (Ran-binding protein 1) (RanBP1) |
| A0A0A0MRJ6 | A0A0A0MRJ6_HUMAN | Protein-L-isoaspartate O-methyltransferase (EC 2.1.1.77) |
| P05062 | ALDOB_HUMAN | Fructose-bisphosphate aldolase B (EC 4.1.2.13) (Liver-type aldolase) |
| P31939 | PUR9_HUMAN | Bifunctional purine biosynthesis protein PURH [Cleaved into: Bifunctional purine biosynthesis protein PURH, N-terminally processed] [Includes: Phosphoribosylaminoimidazolecarboxamide formyltransferase (EC 2.1.2.3) (5-aminoimidazole-4-carboxamide ribonucleotide formyltransferase) (AICAR transformylase); IMP cyclohydrolase (EC 3.5.4.10) (ATIC) (IMP synthase) (Inosinicase)] |
| Q12874 | SF3A3_HUMAN | Splicing factor 3A subunit 3 (SF3a60) (Spliceosome-associated protein 61) (SAP 61) |
| Q08752 | PPID_HUMAN | Peptidyl-prolyl cis-trans isomerase D (PPIase D) (EC 5.2.1.8) (40 kDa peptidyl-prolyl cis-trans isomerase) (Cyclophilin-40) (CYP-40) (Cyclophilin-related protein) (Rotamase D) |
| Q17R60 | IMPG1_HUMAN | Interphotoreceptor matrix proteoglycan 1 (Interphotoreceptor matrix proteoglycan of 150 kDa) (IPM-150) (Sialoprotein associated with cones and rods) |
| P46108 | CRK_HUMAN | Adapter molecule crk (Proto-oncogene c-Crk) (p38) |
| P60981 | DEST_HUMAN | Destrin (Actin-depolymerizing factor) (ADF) |
| P35244 | RFA3_HUMAN | Replication protein A 14 kDa subunit (RP-A p14) (Replication factor A protein 3) (RF-A protein 3) |
| P20962 | PTMS_HUMAN | Parathymosin |
| P45974 | UBP5_HUMAN | Ubiquitin carboxyl-terminal hydrolase 5 (EC 3.4.19.12) (Deubiquitinating enzyme 5) (Isopeptidase T) (Ubiquitin thioesterase 5) (Ubiquitin-specific-processing protease 5) |
| Q96F85 | CNRP1_HUMAN | CB1 cannabinoid receptor-interacting protein 1 (CRIP-1) |
| P69905 | HBA_HUMAN | Hemoglobin subunit alpha (Alpha-globin) (Hemoglobin alpha chain) |
| P13861 | KAP2_HUMAN | cAMP-dependent protein kinase type II-alpha regulatory subunit |
| P16403 | H12_HUMAN | Histone H1.2 (Histone H1c) (Histone H1d) (Histone H1s-1) |
| P54819 | KAD2_HUMAN | Adenylate kinase 2, mitochondrial (AK 2) (EC 2.7.4.3) (ATP-AMP transphosphorylase 2) (ATP:AMP phosphotransferase) (Adenylate monophosphate kinase) [Cleaved into: Adenylate kinase 2, mitochondrial, N-terminally processed] |
| O15212 | PFD6_HUMAN | Prefoldin subunit 6 (Protein Ke2) |
| P49458 | SRP09_HUMAN | Signal recognition particle 9 kDa protein (SRP9) |
| P09429 | HMGB1_HUMAN | High mobility group protein B1 (High mobility group protein 1) (HMG-1) |
| O94905 | ERLN2_HUMAN | Erlin-2 (Endoplasmic reticulum lipid raft-associated protein 2) (Stomatin-prohibitin-flotillin-HflC/K domain-containing protein 2) (SPFH domain-containing protein 2) |
| P69891 | HBG1_HUMAN | Hemoglobin subunit gamma-1 (Gamma-1-globin) (Hb F Agamma) (Hemoglobin gamma-1 chain) (Hemoglobin gamma-A chain) |
| F8WF69 | F8WF69_HUMAN | Clathrin light chain |
| P09496 | CLCA_HUMAN | Clathrin light chain A (Lca) |
| P06753 | TPM3_HUMAN | Tropomyosin alpha-3 chain (Gamma-tropomyosin) (Tropomyosin-3) (Tropomyosin-5) (hTM5) |
| P31930 | QCR1_HUMAN | Cytochrome b-c1 complex subunit 1, mitochondrial (Complex III subunit 1) (Core protein I) (Ubiquinol-cytochrome-c reductase complex core protein 1) |
| P02042 | HBD_HUMAN | Hemoglobin subunit delta (Delta-globin) (Hemoglobin delta chain) |
| Q9H6Z4 | RANB3_HUMAN | Ran-binding protein 3 (RanBP3) |
| P68871 | HBB_HUMAN | Hemoglobin subunit beta (Beta-globin) (Hemoglobin beta chain) [Cleaved into: LVV-hemorphin-7; Spinorphin] |
| P62937 | PPIA_HUMAN | Peptidyl-prolyl cis-trans isomerase A (PPIase A) (EC 5.2.1.8) (Cyclophilin A) (Cyclosporin A-binding protein) (Rotamase A) [Cleaved into: Peptidyl-prolyl cis-trans isomerase A, N-terminally processed] |
| P01042 | KNG1_HUMAN | Kininogen-1 (Alpha-2-thiol proteinase inhibitor) (Fitzgerald factor) (High molecular weight kininogen) (HMWK) (Williams-Fitzgerald-Flaujeac factor) [Cleaved into: Kininogen-1 heavy chain; T-kinin (Ile-Ser-Bradykinin); Bradykinin (Kallidin I); Lysyl-bradykinin (Kallidin II); Kininogen-1 light chain; Low molecular weight growth-promoting factor] |
| P14854 | CX6B1_HUMAN | Cytochrome c oxidase subunit 6B1 (Cytochrome c oxidase subunit VIb isoform 1) (COX VIb-1) |
| Q96AE4 | FUBP1_HUMAN | Far upstream element-binding protein 1 (FBP) (FUSE-binding protein 1) (DNA helicase V) (hDH V) |
| P10412 | H14_HUMAN | Histone H1.4 (Histone H1b) (Histone H1s-4) |
| P09972 | ALDOC_HUMAN | Fructose-bisphosphate aldolase C (EC 4.1.2.13) (Brain-type aldolase) |
| A0A0A0MR02 | A0A0A0MR02_HUMAN | Voltage-dependent anion-selective channel protein 2 (Fragment) |
| P04075 | ALDOA_HUMAN | Fructose-bisphosphate aldolase A (EC 4.1.2.13) (Lung cancer antigen NY-LU-1) (Muscle-type aldolase) |
| P30040 | ERP29_HUMAN | Endoplasmic reticulum resident protein 29 (ERp29) (Endoplasmic reticulum resident protein 28) (ERp28) (Endoplasmic reticulum resident protein 31) (ERp31) |
| Q03252 | LMNB2_HUMAN | Lamin-B2 |
| Q9Y3I0 | RTCB_HUMAN | tRNA-splicing ligase RtcB homolog (EC 6.5.1.3) |
| C9JSK0 | C9JSK0_HUMAN | Protein unc-119 homolog A (Fragment) |
| A6NIH7 | U119B_HUMAN | Protein unc-119 homolog B |
| Q9H479 | FN3K_HUMAN | Fructosamine-3-kinase (EC 2.7.1.-) |
| J3KN42 | J3KN42_HUMAN | Calcium-binding mitochondrial carrier protein SCaMC-1 |
| P42166 | LAP2A_HUMAN | Lamina-associated polypeptide 2, isoform alpha (Thymopoietin isoform alpha) (TP alpha) (Thymopoietin-related peptide isoform alpha) (TPRP isoform alpha) [Cleaved into: Thymopoietin (TP) (Splenin); Thymopentin (TP5)] |
| P62195 | PRS8_HUMAN | 26S proteasome regulatory subunit 8 (26S proteasome AAA-ATPase subunit RPT6) (Proteasome 26S subunit ATPase 5) (Proteasome subunit p45) (Thyroid hormone receptor-interacting protein 1) (TRIP1) (p45/SUG) |
| P43686 | PRS6B_HUMAN | 26S proteasome regulatory subunit 6B (26S proteasome AAA-ATPase subunit RPT3) (MB67-interacting protein) (MIP224) (Proteasome 26S subunit ATPase 4) (Tat-binding protein 7) (TBP-7) |
| Q9BRA2 | TXD17_HUMAN | Thioredoxin domain-containing protein 17 (14 kDa thioredoxin-related protein) (TRP14) (Protein 42-9-9) (Thioredoxin-like protein 5) |
| P02511 | CRYAB_HUMAN | Alpha-crystallin B chain (Alpha(B)-crystallin) (Heat shock protein beta-5) (HspB5) (Renal carcinoma antigen NY-REN-27) (Rosenthal fiber component) |
| P31040 | SDHA_HUMAN | Succinate dehydrogenase [ubiquinone] flavoprotein subunit, mitochondrial (EC 1.3.5.1) (Flavoprotein subunit of complex II) (Fp) |
| Q09666 | AHNK_HUMAN | Neuroblast differentiation-associated protein AHNAK (Desmoyokin) |
| P23297 | S10A1_HUMAN | Protein S100-A1 (S-100 protein alpha chain) (S-100 protein subunit alpha) (S100 calcium-binding protein A1) |
| A6NFX8 | A6NFX8_HUMAN | ADP-sugar pyrophosphatase |
| G8JLA2 | G8JLA2_HUMAN | Myosin light polypeptide 6 |
| P25788 | PSA3_HUMAN | Proteasome subunit alpha type-3 (EC 3.4.25.1) (Macropain subunit C8) (Multicatalytic endopeptidase complex subunit C8) (Proteasome component C8) |
| P61224 | RAP1B_HUMAN | Ras-related protein Rap-1b (GTP-binding protein smg p21B) |
| P10523 | ARRS_HUMAN | S-arrestin (48 kDa protein) (Retinal S-antigen) (S-AG) (Rod photoreceptor arrestin) |
| P37837 | TALDO_HUMAN | Transaldolase (EC 2.2.1.2) |
| Q01995 | TAGL_HUMAN | Transgelin (22 kDa actin-binding protein) (Protein WS3-10) (Smooth muscle protein 22-alpha) (SM22-alpha) |
| J3KNF8 | J3KNF8_HUMAN | Cytochrome b5 type B (Cytochrome b5 type B (Outer mitochondrial membrane), isoform CRA_a) |
| P48735 | IDHP_HUMAN | Isocitrate dehydrogenase [NADP], mitochondrial (IDH) (EC 1.1.1.42) (ICD-M) (IDP) (NADP(+)-specific ICDH) (Oxalosuccinate decarboxylase) |
| Q13162 | PRDX4_HUMAN | Peroxiredoxin-4 (EC 1.11.1.15) (Antioxidant enzyme AOE372) (AOE37-2) (Peroxiredoxin IV) (Prx-IV) (Thioredoxin peroxidase AO372) (Thioredoxin-dependent peroxide reductase A0372) |
| O75347 | TBCA_HUMAN | Tubulin-specific chaperone A (TCP1-chaperonin cofactor A) (Tubulin-folding cofactor A) (CFA) |
| P50213 | IDH3A_HUMAN | Isocitrate dehydrogenase [NAD] subunit alpha, mitochondrial (EC 1.1.1.41) (Isocitric dehydrogenase subunit alpha) (NAD(+)-specific ICDH subunit alpha) |
| P51858 | HDGF_HUMAN | Hepatoma-derived growth factor (HDGF) (High mobility group protein 1-like 2) (HMG-1L2) |
| P30084 | ECHM_HUMAN | Enoyl-CoA hydratase, mitochondrial (EC 4.2.1.17) (Enoyl-CoA hydratase 1) (Short-chain enoyl-CoA hydratase) (SCEH) |
| A0A0A6YY92 | A0A0A6YY92_HUMAN | Adenylosuccinate lyase (ASL) (EC 4.3.2.2) (Adenylosuccinase) |
| Q99497 | PARK7_HUMAN | Protein DJ-1 (DJ-1) (Oncogene DJ1) (Parkinson disease protein 7) (Parkinsonism-associated deglycase) (Protein deglycase DJ-1) (EC 3.1.2.-) (EC 3.5.1.124) |
| Q8IUE6 | H2A2B_HUMAN | Histone H2A type 2-B |
| P16104 | H2AX_HUMAN | Histone H2AX (H2a/x) (Histone H2A.X) |
| Q04760 | LGUL_HUMAN | Lactoylglutathione lyase (EC 4.4.1.5) (Aldoketomutase) (Glyoxalase I) (Glx I) (Ketone-aldehyde mutase) (Methylglyoxalase) (S-D-lactoylglutathione methylglyoxal lyase) |
| P83916 | CBX1_HUMAN | Chromobox protein homolog 1 (HP1Hsbeta) (Heterochromatin protein 1 homolog beta) (HP1 beta) (Heterochromatin protein p25) (M31) (Modifier 1 protein) (p25beta) |
| P06748 | NPM_HUMAN | Nucleophosmin (NPM) (Nucleolar phosphoprotein B23) (Nucleolar protein NO38) (Numatrin) |
| Q8TF21 | ANR24_HUMAN | Ankyrin repeat domain-containing protein 24 |
| O75438 | NDUB1_HUMAN | NADH dehydrogenase [ubiquinone] 1 beta subcomplex subunit 1 (Complex I-MNLL) (CI-MNLL) (NADH-ubiquinone oxidoreductase MNLL subunit) [Cleaved into: NADH dehydrogenase [ubiquinone] 1 beta subcomplex subunit 1, N-terminally processed] |
| P17066 | HSP76_HUMAN | Heat shock 70 kDa protein 6 (Heat shock 70 kDa protein B') |
| P54652 | HSP72_HUMAN | Heat shock-related 70 kDa protein 2 (Heat shock 70 kDa protein 2) |
| P27338 | AOFB_HUMAN | Amine oxidase [flavin-containing] B (EC 1.4.3.4) (Monoamine oxidase type B) (MAO-B) |
| Q2TAY7 | SMU1_HUMAN | WD40 repeat-containing protein SMU1 (Smu-1 suppressor of mec-8 and unc-52 protein homolog) [Cleaved into: WD40 repeat-containing protein SMU1, N-terminally processed] |
| Q16718 | NDUA5_HUMAN | NADH dehydrogenase [ubiquinone] 1 alpha subcomplex subunit 5 (Complex I subunit B13) (Complex I-13kD-B) (CI-13kD-B) (NADH-ubiquinone oxidoreductase 13 kDa-B subunit) |
| P20674 | COX5A_HUMAN | Cytochrome c oxidase subunit 5A, mitochondrial (Cytochrome c oxidase polypeptide Va) |
| Q5SSJ5 | HP1B3_HUMAN | Heterochromatin protein 1-binding protein 3 (Protein HP1-BP74) |
| P49189 | AL9A1_HUMAN | 4-trimethylaminobutyraldehyde dehydrogenase (TMABADH) (EC 1.2.1.47) (Aldehyde dehydrogenase E3 isozyme) (Aldehyde dehydrogenase family 9 member A1) (EC 1.2.1.3) (Gamma-aminobutyraldehyde dehydrogenase) (EC 1.2.1.19) (R-aminobutyraldehyde dehydrogenase) [Cleaved into: 4-trimethylaminobutyraldehyde dehydrogenase, N-terminally processed] |
| P28838 | AMPL_HUMAN | Cytosol aminopeptidase (EC 3.4.11.1) (Leucine aminopeptidase 3) (LAP-3) (Leucyl aminopeptidase) (Peptidase S) (Proline aminopeptidase) (EC 3.4.11.5) (Prolyl aminopeptidase) |
| Q9BZV3 | IMPG2_HUMAN | Interphotoreceptor matrix proteoglycan 2 (Interphotoreceptor matrix proteoglycan of 200 kDa) (IPM 200) (Sialoprotein associated with cones and rods proteoglycan) (Spacrcan) |
| P58876 | H2B1D_HUMAN | Histone H2B type 1-D (HIRA-interacting protein 2) (Histone H2B.1 B) (Histone H2B.b) (H2B/b) |
| Q17RW2 | COOA1_HUMAN | Collagen alpha-1(XXIV) chain |
| P84243 | H33_HUMAN | Histone H3.3 |
| P12271 | RLBP1_HUMAN | Retinaldehyde-binding protein 1 (Cellular retinaldehyde-binding protein) |
| P00441 | SODC_HUMAN | Superoxide dismutase [Cu-Zn] (EC 1.15.1.1) (Superoxide dismutase 1) (hSod1) |
| O14558 | HSPB6_HUMAN | Heat shock protein beta-6 (HspB6) (Heat shock 20 kDa-like protein p20) |
| C9JWQ4 | C9JWQ4_HUMAN | Mitogen-activated protein kinase 8 (Fragment) |
| P98179 | RBM3_HUMAN | RNA-binding protein 3 (RNA-binding motif protein 3) (RNPL) |
| P07737 | PROF1_HUMAN | Profilin-1 (Epididymis tissue protein Li 184a) (Profilin I) |
| O60493 | SNX3_HUMAN | Sorting nexin-3 (Protein SDP3) |
| Q9UI30 | TR112_HUMAN | Multifunctional methyltransferase subunit TRM112-like protein (tRNA methyltransferase 112 homolog) |
| Q99496 | RING2_HUMAN | E3 ubiquitin-protein ligase RING2 (EC 2.3.2.27) (Huntingtin-interacting protein 2-interacting protein 3) (HIP2-interacting protein 3) (Protein DinG) (RING finger protein 1B) (RING1b) (RING finger protein 2) (RING finger protein BAP-1) (RING-type E3 ubiquitin transferase RING2) |
| P14174 | MIF_HUMAN | Macrophage migration inhibitory factor (MIF) (EC 5.3.2.1) (Glycosylation-inhibiting factor) (GIF) (L-dopachrome isomerase) (L-dopachrome tautomerase) (EC 5.3.3.12) (Phenylpyruvate tautomerase) |
| P51991 | ROA3_HUMAN | Heterogeneous nuclear ribonucleoprotein A3 (hnRNP A3) |
| Q92973 | TNPO1_HUMAN | Transportin-1 (Importin beta-2) (Karyopherin beta-2) (M9 region interaction protein) (MIP) |
| Q15287 | RNPS1_HUMAN | RNA-binding protein with serine-rich domain 1 (SR-related protein LDC2) |
| O43186 | CRX_HUMAN | Cone-rod homeobox protein |
| P63241 | IF5A1_HUMAN | Eukaryotic translation initiation factor 5A-1 (eIF-5A-1) (eIF-5A1) (Eukaryotic initiation factor 5A isoform 1) (eIF-5A) (Rev-binding factor) (eIF-4D) |
| P09110 | THIK_HUMAN | 3-ketoacyl-CoA thiolase, peroxisomal (EC 2.3.1.16) (Acetyl-CoA acyltransferase) (Beta-ketothiolase) (Peroxisomal 3-oxoacyl-CoA thiolase) |
| Q8WWY3 | PRP31_HUMAN | U4/U6 small nuclear ribonucleoprotein Prp31 (Pre-mRNA-processing factor 31) (Serologically defined breast cancer antigen NY-BR-99) (U4/U6 snRNP 61 kDa protein) (Protein 61K) (hPrp31) |
| P26583 | HMGB2_HUMAN | High mobility group protein B2 (High mobility group protein 2) (HMG-2) |
| P50416 | CPT1A_HUMAN | Carnitine O-palmitoyltransferase 1, liver isoform (CPT1-L) (EC 2.3.1.21) (Carnitine O-palmitoyltransferase I, liver isoform) (CPT I) (CPTI-L) (Carnitine palmitoyltransferase 1A) |
| O43347 | MSI1H_HUMAN | RNA-binding protein Musashi homolog 1 (Musashi-1) |
| P49755 | TMEDA_HUMAN | Transmembrane emp24 domain-containing protein 10 (21 kDa transmembrane-trafficking protein) (S31III125) (S31I125) (Tmp-21-I) (Transmembrane protein Tmp21) (p23) (p24 family protein delta-1) (p24delta1) (p24delta) |
| Q9HBL7 | PLRKT_HUMAN | Plasminogen receptor (KT) (Plg-R(KT)) |
| Q14103 | HNRPD_HUMAN | Heterogeneous nuclear ribonucleoprotein D0 (hnRNP D0) (AU-rich element RNA-binding protein 1) |
| P55084 | ECHB_HUMAN | Trifunctional enzyme subunit beta, mitochondrial (TP-beta) [Includes: 3-ketoacyl-CoA thiolase (EC 2.3.1.16) (Acetyl-CoA acyltransferase) (Beta-ketothiolase)] |
