# Supplementary material for: Proteomic analysis of the human retina reveals region-specific susceptibilities to metabolic- and oxidative stress-related diseases
Source: PLoS One. 2018 Feb 21;13(2):e0193250. doi: 10.1371/journal.pone.0193250 (PMC5821407; doi:10.1371/journal.pone.0193250)
Supplement: S6 Table — (DOCX) [file pone.0193250.s017.docx]

**Supplemental Table 6. Pathways identified in the foveomacular retina.**

| **Pathways** | [**Reference**](http://pantherdb.org/tools/compareToRefList.jsp?sortOrder=2&sortList=Homo%20sapiens) | [**Dataset**](http://pantherdb.org/tools/compareToRefList.jsp?sortOrder=2&sortList=Client%20Text%20Box%20Input&sortField=num) | [**Expected**](http://pantherdb.org/tools/compareToRefList.jsp?sortOrder=2&sortList=Client%20Text%20Box%20Input&sortField=exp) | **Fold**  **Enrichment** | [**+/-**](http://pantherdb.org/tools/compareToRefList.jsp?sortOrder=1&sortList=Client%20Text%20Box%20Input&sortField=rep) | [**P value**](http://pantherdb.org/tools/compareToRefList.jsp?sortOrder=1&sortList=Client%20Text%20Box%20Input&sortField=pval) | **-log(P-value)** |
| --- | --- | --- | --- | --- | --- | --- | --- |
| Cytoskeletal regulation by Rho GTPase | [82](http://pantherdb.org/tools/gxIdsList.do?acc=P00016&reflist=1) | [14](http://pantherdb.org/tools/gxIdsList.do?acc=P00016&list=Client%20Text%20Box%20Input&organism=Homo%20sapiens) | 0.68 | 20.49 | + | 1.81E-14 | 13.74232143 |
| Huntington disease | [142](http://pantherdb.org/tools/gxIdsList.do?acc=P00029&reflist=1) | [15](http://pantherdb.org/tools/gxIdsList.do?acc=P00029&list=Client%20Text%20Box%20Input&organism=Homo%20sapiens) | 1.18 | 12.68 | + | 1.86E-12 | 11.73048706 |
| Integrin signaling pathway | [194](http://pantherdb.org/tools/gxIdsList.do?acc=P00034&reflist=1) | [10](http://pantherdb.org/tools/gxIdsList.do?acc=P00034&list=Client%20Text%20Box%20Input&organism=Homo%20sapiens) | 1.62 | 6.19 | + | 6.48E-06 | 5.188424994 |
| [Ras Pathway](javascript:openDiagramWindow('/pathway/pathwayDiagram.jsp?color=1&catsInfo=true&catAccession=P04393');) | [74](http://pantherdb.org/tools/gxIdsList.do?acc=P04393&reflist=1) | [4](http://pantherdb.org/tools/gxIdsList.do?acc=P04393&list=Client%20Text%20Box%20Input&organism=Homo%20sapiens) | 0.62 | 6.49 | + | 3.61E-03 | 2.442492798 |
| Inflammation mediated by chemokine  and cytokine signaling pathway | [261](http://pantherdb.org/tools/gxIdsList.do?acc=P00031&reflist=1) | [7](http://pantherdb.org/tools/gxIdsList.do?acc=P00031&list=Client%20Text%20Box%20Input&organism=Homo%20sapiens) | 2.17 | 3.22 | + | 6.64E-03 | 2.177831921 |
| Anandamide degradation | [1](http://pantherdb.org/tools/gxIdsList.do?acc=P05728&reflist=1) | [1](http://pantherdb.org/tools/gxIdsList.do?acc=P05728&list=Client%20Text%20Box%20Input&organism=Homo%20sapiens) | 0.01 | > 100 | + | 8.30E-03 | 2.080921908 |
| Nicotinic acetylcholine receptor  signaling pathway | [101](http://pantherdb.org/tools/gxIdsList.do?acc=P00044&reflist=1) | [4](http://pantherdb.org/tools/gxIdsList.do?acc=P00044&list=Client%20Text%20Box%20Input&organism=Homo%20sapiens) | 0.84 | 4.75 | + | 1.05E-02 | 1.978810701 |
| Axon guidance mediated by  semaphorins | [23](http://pantherdb.org/tools/gxIdsList.do?acc=P00007&reflist=1) | [2](http://pantherdb.org/tools/gxIdsList.do?acc=P00007&list=Client%20Text%20Box%20Input&organism=Homo%20sapiens) | 0.19 | 10.44 | + | 1.61E-02 | 1.793174124 |
| Alzheimer disease-presenilin pathway | [122](http://pantherdb.org/tools/gxIdsList.do?acc=P00004&reflist=1) | [4](http://pantherdb.org/tools/gxIdsList.do?acc=P00004&list=Client%20Text%20Box%20Input&organism=Homo%20sapiens) | 1.02 | 3.93 | + | 1.97E-02 | 1.705533774 |
| [Synaptic vesicle trafficking](javascript:openDiagramWindow('/pathway/pathwayDiagram.jsp?color=1&catsInfo=true&catAccession=P05734');) | [29](http://pantherdb.org/tools/gxIdsList.do?acc=P05734&reflist=1) | [2](http://pantherdb.org/tools/gxIdsList.do?acc=P05734&list=Client%20Text%20Box%20Input&organism=Homo%20sapiens) | 0.24 | 8.28 | + | 2.48E-02 | 1.605548319 |
| Angiotensin II-stimulated signaling  through G proteins and beta-arrestin | [38](http://pantherdb.org/tools/gxIdsList.do?acc=P05911&reflist=1) | [2](http://pantherdb.org/tools/gxIdsList.do?acc=P05911&list=Client%20Text%20Box%20Input&organism=Homo%20sapiens) | 0.32 | 6.32 | + | 4.06E-02 | 1.391473966 |
| Succinate to proprionate conversion | [5](http://pantherdb.org/tools/gxIdsList.do?acc=P02777&reflist=1) | [1](http://pantherdb.org/tools/gxIdsList.do?acc=P02777&list=Client%20Text%20Box%20Input&organism=Homo%20sapiens) | 0.04 | 24 | + | 4.08E-02 | 1.389339837 |
| Methylmalonyl pathway | [5](http://pantherdb.org/tools/gxIdsList.do?acc=P02755&reflist=1) | [1](http://pantherdb.org/tools/gxIdsList.do?acc=P02755&list=Client%20Text%20Box%20Input&organism=Homo%20sapiens) | 0.04 | 24 | + | 4.08E-02 | 1.389339837 |
| Cadherin signaling pathway | [158](http://pantherdb.org/tools/gxIdsList.do?acc=P00012&reflist=1) | [4](http://pantherdb.org/tools/gxIdsList.do?acc=P00012&list=Client%20Text%20Box%20Input&organism=Homo%20sapiens) | 1.32 | 3.04 | + | 4.41E-02 | 1.355561411 |
| Heterotrimeric G-protein signaling  pathway-Gi alpha and Gs  alpha mediated pathway | [163](http://pantherdb.org/tools/gxIdsList.do?acc=P00026&reflist=1) | [4](http://pantherdb.org/tools/gxIdsList.do?acc=P00026&list=Client%20Text%20Box%20Input&organism=Homo%20sapiens) | 1.36 | 2.95 | + | 4.84E-02 | 1.315154638 |
