# Supplementary material for: Proteomic analysis of the human retina reveals region-specific susceptibilities to metabolic- and oxidative stress-related diseases
Source: PLoS One. 2018 Feb 21;13(2):e0193250. doi: 10.1371/journal.pone.0193250 (PMC5821407; doi:10.1371/journal.pone.0193250)
Supplement: S7 Table — (DOCX) [file pone.0193250.s018.docx]

**Supplemental Table 7. Pathway identified in the juxta-macular retina.**

| **Pathways** | **Reference** | **Dataset** | **Expected** | **Fold Enrichment** | **+/-** | **P value** | **-log(P-value)** |
| --- | --- | --- | --- | --- | --- | --- | --- |
| Huntington disease | 142 | 8 | 0.68 | 11.83 | + | 4.68E-07 | 6.329754147 |
| Cytoskeletal regulation by  Rho GTPase | 82 | 6 | 0.39 | 15.37 | + | 3.08E-06 | 5.511449283 |
| Parkinson disease | 100 | 6 | 0.48 | 12.6 | + | 9.47E-06 | 5.023650021 |
| Apoptosis signaling pathway | 119 | 5 | 0.57 | 8.82 | + | 2.81E-04 | 3.55129368 |
| Heterotrimeric G-protein signaling pathway-rod outer segment phototransduction | 36 | 3 | 0.17 | 17.5 | + | 7.19E-04 | 3.14327111 |
| Anandamide degradation | 1 | 1 | 0 | > 100 | + | 4.75E-03 | 2.32330639 |
| Synaptic vesicle trafficking | 29 | 2 | 0.14 | 14.48 | + | 8.63E-03 | 2.063989204 |
| Glutamine glutamate conversion | 4 | 1 | 0.02 | 52.51 | + | 1.89E-02 | 1.723538196 |
| Pentose phosphate pathway | 8 | 1 | 0.04 | 26.25 | + | 3.74E-02 | 1.427128398 |
| VEGF signaling pathway | 69 | 2 | 0.33 | 6.09 | + | 4.32E-02 | 1.364516253 |
