# Supplementary material for: Proteomic analysis of the human retina reveals region-specific susceptibilities to metabolic- and oxidative stress-related diseases
Source: PLoS One. 2018 Feb 21;13(2):e0193250. doi: 10.1371/journal.pone.0193250 (PMC5821407; doi:10.1371/journal.pone.0193250)
Supplement: S8 Table — (DOCX) [file pone.0193250.s019.docx]

**Supplemental Table 8. Pathways identified in the peripheral retina.**

| **Pathway** | **Reference** | **Dataset** | **Expected** | **Fold Enrichment** | **+/-** | **P value** | **-log(P-value)** |
| --- | --- | --- | --- | --- | --- | --- | --- |
| Cytoskeletal regulation by Rho GTPase | 82 | 8 | 0.55 | 14.64 | + | 1.02E-07 | 6.991399828 |
| Huntington disease | 142 | 8 | 0.95 | 8.45 | + | 5.91E-06 | 5.228412519 |
| Parkinson disease | 100 | 5 | 0.67 | 7.5 | + | 5.99E-04 | 3.222573178 |
| Apoptosis signaling pathway | 119 | 5 | 0.79 | 6.3 | + | 1.29E-03 | 2.88941029 |
| Heterotrimeric G-protein signaling  pathway-rod outer segment phototransduction | 36 | 3 | 0.24 | 12.5 | + | 1.89E-03 | 2.723538196 |
| Fructose galactose metabolism | 12 | 2 | 0.08 | 25 | + | 3.01E-03 | 2.521433504 |
| Anandamide degradation | 1 | 1 | 0.01 | > 100 | + | 6.64E-03 | 2.177831921 |
| Glycolysis | 20 | 2 | 0.13 | 15 | + | 8.09E-03 | 2.092051478 |
| VEGF signaling pathway | 69 | 3 | 0.46 | 6.52 | + | 1.14E-02 | 1.943095149 |
| Synaptic vesicle trafficking | 29 | 2 | 0.19 | 10.35 | + | 1.64E-02 | 1.785156152 |
| DNA replication | 30 | 2 | 0.2 | 10 | + | 1.74E-02 | 1.759450752 |
| Glutamine glutamate conversion | 4 | 1 | 0.03 | 37.5 | + | 2.63E-02 | 1.580044252 |
| Angiogenesis | 174 | 4 | 1.16 | 3.45 | + | 2.98E-02 | 1.525783736 |
| Succinate to proprionate conversion | 5 | 1 | 0.03 | 30 | + | 3.28E-02 | 1.484126156 |
